# Genome-wide Functional Analysis of *Plasmodium* Protein Phosphatases Reveals Key Regulators of Parasite Development and Differentiation

**DOI:** 10.1016/j.chom.2014.05.020

**Published:** 2014-07-09

**Authors:** David S. Guttery, Benoit Poulin, Abhinay Ramaprasad, Richard J. Wall, David J.P. Ferguson, Declan Brady, Eva-Maria Patzewitz, Sarah Whipple, Ursula Straschil, Megan H. Wright, Alyaa M.A.H. Mohamed, Anand Radhakrishnan, Stefan T. Arold, Edward W. Tate, Anthony A. Holder, Bill Wickstead, Arnab Pain, Rita Tewari

**Affiliations:** 1 Centre for Genetics and Genomics, School of Life Sciences, Queens Medical Centre, University of Nottingham, Nottingham NG2 7UH, UK; 2 Computational Bioscience Research Center (CBRC), Biological and Environmental Sciences and Engineering (BESE) Division, King Abdullah University of Science and Technology, Thuwal 23955-6900, Kingdom of Saudi Arabia; 3 Nuffield Department of Clinical Laboratory Science, University of Oxford, John Radcliffe Hospital, Oxford OX3 9DU, UK; 4 Division of Cell and Molecular Biology, Imperial College London, Exhibition Road, London SW7 2AZ, UK; 5 Department of Chemistry, Imperial College London, Exhibition Road, London SW7 2AZ, UK; 6 Division of Parasitology, MRC National Institute for Medical Research, Mill Hill, London NW7 1AA, UK

## Abstract

Reversible protein phosphorylation regulated by kinases and phosphatases controls many cellular processes. Although essential functions for the malaria parasite kinome have been reported, the roles of most protein phosphatases (PPs) during *Plasmodium* development are unknown. We report a functional analysis of the *Plasmodium berghei* protein phosphatome, which exhibits high conservation with the *P. falciparum* phosphatome and comprises 30 predicted PPs with differential and distinct expression patterns during various stages of the life cycle. Gene disruption analysis of *P. berghei* PPs reveals that half of the genes are likely essential for asexual blood stage development, whereas six are required for sexual development/sporogony in mosquitoes. Phenotypic screening coupled with transcriptome sequencing unveiled morphological changes and altered gene expression in deletion mutants of two *N-*myristoylated PPs. These findings provide systematic functional analyses of PPs in *Plasmodium*, identify how phosphatases regulate parasite development and differentiation, and can inform the identification of drug targets for malaria.

## Highlights

• Phylogenetic analysis identifies 30 *Plasmodium berghei* protein phosphatases (PPs)
• Functional analysis reveals role for six PPs in sexual development/sporogony
• Two *N-*myristoylated PPs play key roles in sex allocation and parasite transmission
• RNA-Seq highlights significantly altered gene clusters in the *N-*myristoylated PP mutants

## Graphical Abstract

**Figure:**
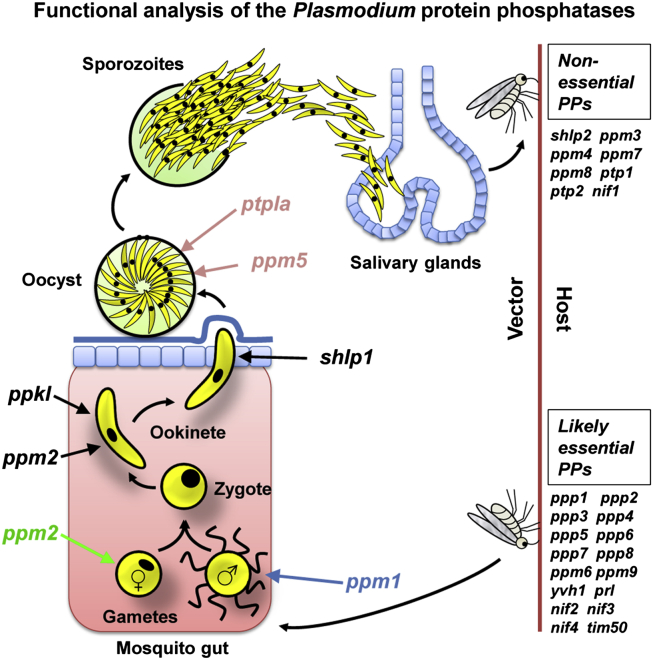

## Teaser

Protein phosphorylation regulated by kinases and phosphatases plays important roles in the *Plasmodium* life cycle. By performing a genome-wide functional screen, Guttery et al. examine protein phosphatases in *Plasmodium berghei* and identify phosphatases likely essential for asexual development in host blood as well as those required for sexual/sporogony development in mosquitos.

## Introduction

Malaria, caused by infection with the apicomplexan parasite *Plasmodium*, is transmitted via the female *Anopheles* mosquito and in 2012 resulted in approximately 207 million clinical infections and over 600,000 deaths (WHO, 2013). The *Plasmodium* life cycle progresses through several morphologically distinct developmental stages, including asexual proliferation in hepatocytes, followed by clinically overt intraerythrocytic multiplication in the vertebrate host. Ingestion of developmentally arrested gametocytes initiates sexual development of the parasite in the mosquito, with eventual migration to the salivary glands and transmission during feeding (Bannister and Sherman, 2009). During each stage the parasite utilizes a number of signal transduction mechanisms, including reversible protein phosphorylation catalyzed by protein kinases (PKs) and phosphatases (PPs). This mechanism of signaling is a conserved, ubiquitous regulatory process for many eukaryotic and prokaryotic cellular pathways (Cohen, 2000). However, while PKs are well recognized as important therapeutic targets (Doerig et al., 2010), PPs are only now emerging as targets for clinical intervention (Moorhead et al., 2007).

Sequence analysis of the *Plasmodium falciparum* parasite has revealed approximately 85 putative PK and 27 putative PP catalytic subunits encoded in its genome (the *Plasmodium* protein phosphatome being one of the smallest of the eukaryotic phyla) (Ward et al., 2004; Wilkes and Doerig, 2008). Recent functional analyses of the entire kinome in both the human *P. falciparum* and rodent *Plasmodium berghei* models have shown asexual stage essentiality for over half of their kinases, with a further 14 PKs having a specific function during sexual development (Solyakov et al., 2011; Tewari et al., 2010).

Although it was recently recognized as a putative target for therapeutic intervention, there is lack of systematic functional analyses of the complementary *Plasmodium* phosphatome (previously classified into four major groups: phosphoprotein phosphatases [PPPs], metallo-dependent protein phosphatases [PPMs], protein tyrosine phosphatases [PTPs], and NLI-interacting factor-like phosphatases [NIFs], as well as a number of smaller classes) (Kutuzov and Andreeva, 2008; Moorhead et al., 2009; Wilkes and Doerig, 2008). Two nonconventional PPs, one containing an N-terminal β-propeller formed by kelch-like motifs (PPKL) and the other a *Shewanella-*like PP (SHLP1), are required during ookinete-to-oocyst transition and subsequent transmission in the in vivo rodent malaria model *P. berghei* (Guttery et al., 2012; Patzewitz et al., 2013). More recently, a second SHLP family member (SHLP2) was implicated in dephosphorylation of the host protein Band 3 during merozoite invasion of erythrocytes (Fernandez-Pol et al., 2013). However, the functional role of no other PP in any of the four major groups is known (Moorhead et al., 2009; Wilkes and Doerig, 2008).

Here, we use *P. berghei* to systematically analyze the entire *Plasmodium* protein phosphatome and, where possible, assign functions for each PP throughout the life cycle in vivo, including during mosquito transmission. Furthermore, we elucidate the expression and subcellular localization of each PP by use of endogenously tagged C-terminal GFP fusion proteins. We show that the *P. berghei* protein phosphatome is highly conserved with that of *P. falciparum*, and that expression of each PP is highly variable throughout its life cycle. In our genome-wide deletion study, we assigned functions to 14 PPs, 6 of which are essential for sexual development and differentiation during gamete-to-ookinete/oocyst transition in vivo. In-depth functional analysis of two unique *N-*myristolated PP mutants identified key roles in sex allocation, zygote differentiation, and sporogony. Further global analysis of transcript levels revealed changes in membrane structure, invasion, and cell-cycle and sporogony gene families, substantiating the phenotypic analysis. This systematic functional analysis provides a genome-wide identification of key signaling networks in both asexual blood stages and mosquito transmission. Overall, it is an important study complementary to that of the kinome toward understanding the complex signaling networks via reversible phosphorylation in *Plasmodium*.

## Results and Discussion

### The *Plasmodium* Phosphatome Is Diverse and Largely Conserved Between Species

**Figure 1:**
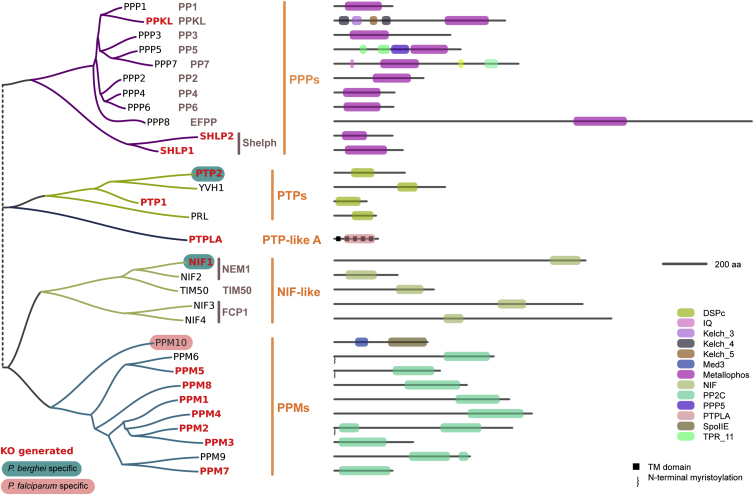
The *Plasmodium* Phosphatome Schematic phylogenetic tree and domain architectures for the PPs of *P. berghei* ANKA and *P. falciparum* 3D7 showing family and subfamily classification. Proteins encoded in only one species are highlighted. Deletion mutants obtained are shown in bold red text. Domain architecture for *P. falciparum* protein is shown unless no ortholog exists (PTP2, NIF1). See also Figure S1.

To define the phosphatome for human and rodent malaria, PPs encoded in the genomes of *P. berghei* and *P. falciparum* were identified by similarity to hidden Markov models of known PP catalytic domains. PFam domains were used to define protein sets with similarity to PPP, PTP, PPM, NIF-like, and PTP-like A families. As previously observed (Wilkes and Doerig, 2008), there are no predicted PPs with good similarity to the low-molecular weight phosphatase (LMWP) or CDC25 families. There are also no good matches to models of SSU72 RNA polymerase II CTD PP or Eyes Absent (EYA) PP. Other PFam domains specific to PP catalytic domains are subclasses of the above families. The 5 identified *Plasmodium* PP families were compared to 4,969 PP-like proteins from 44 diverse eukaryotes (Wickstead et al., 2010), to classify them and eliminate PP-like proteins with confirmed nonprotein PP functions. These data are summarized in Figure 1.

We found that the *P. berghei* and *P. falciparum* phosphatomes consist of 30 and 29 PPs, respectively, encompassing 28 direct orthologs across the 5 PP families described above. Our *P. falciparum* phosphatome includes the 27 previously identified PPs (Wilkes and Doerig, 2008), an additional PPP-type protein (PPP8; PF3D7_1018200), and a PTP-like A homolog (PTPLA; PF3D7_1331600) also annotated as 3-hydroxyacyl-CoA dehydratase (see Table S1, available online, for full list of accession numbers). 3D structural homology modeling supported the presence of a bona fide PP catalytic domain for all included PPs, except for PTPLA and YVH1, for which no suitable template structures are available. However, PfYVH1 has been experimentally proven to be a PP (Kumar et al., 2004). Based on available experimental template structures with substrate, phosphate, and/or metal ions bound, computational structural analysis has further supported the presence and correct position of phosphate and metal-coordinating residues in all PPPs, NIFs, and PPMs, with the exception of PPM8, which shows metal coordinating residues but lacks an obvious phosphate-binding arginine or lysine.

As found with the kinome (Tewari et al., 2010), the phosphatome is highly conserved with only three proteins without direct orthology between *P. falciparum* and *P. berghei* (Figure 1). On the basis of catalytic domain phylogeny and domain architecture, the *Plasmodium* PPP-type PPs can be further classified into subfamilies, with PPP1–PPP7 corresponding to the animal PP1–PP7 types. *Plasmodium* PPPs also include the BSU-like PP PPKL, an EF-hand-containing PP (EFPP), and the two SHLPs, none of which are present in the host (Moorhead et al., 2009).

*Plasmodium* PPMs and PTPs generally do not fall into well-conserved subclasses. The most broadly conserved is PPM8, which is part of a group containing human and yeast pyruvate dehydrogenase PP (Figure S1). The divergent PPM10 contains a domain type also found in the bacterial membrane-associated PP, SpoIIE, and yeast mitochondrial PTC7. This protein is encoded in the *P. falciparum* but not the *P. berghei* genome. The plasmodial PPMs include three proteins containing predicted sites for N-terminal myristoylation (PPM2, PPM5, and PPM6). PPM5 and PPM6 are similar to a group of plant PPMs of unknown function, whereas PPM2 is similar to human PP1G and yeast PTC2 and PTC3. Several of the similar PPMs in other species also contain sites for possible myristoylation, although to our knowledge none has been experimentally validated.

### Expression and Subcellular Localization of the *Plasmodium* PPs Is Highly Diverse

**Figure 2:**
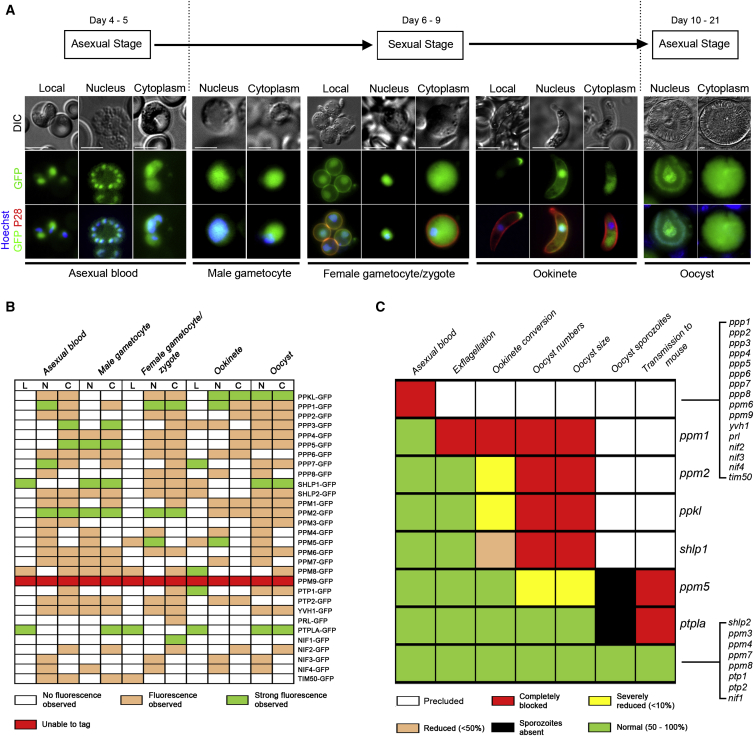
PP Expression and Phenotypic Analysis of 14 PP Mutants (A) Localization of representative PP-GFP classified into three categories: present only in nucleus (Nucleus), diffuse staining (Cytoplasm), or localized to specific cellular domain (Local). Scale bar, 5 μm. Green, GFP; blue, Hoechst; red, Cy3 P28 staining. (B) PP-GFP expression in five key developmental stages. N, nucleus; C, cytoplasm; L, local/heterogeneous. (C) Representation of phenotypic analysis. See also Figures S2 and S3, Table S1, Table S2, and Table S5.

Very little is known about the expression profiles of *Plasmodium* PPs. Therefore, we used single homologous recombination to endogenously tag each of the *P. berghei* PPs with the green fluorescent protein (GFP) at their C terminus and assessed protein expression and localization at five key stages of the parasite life cycle (Figure 2A). A total of 152 individual transfection attempts were performed, resulting in successful tagging of 29 PPs as determined by western blotting and fluorescence microscopy (Figure S2B; Table S1). Despite 18 attempts, we were unable to endogenously tag PPM9, suggesting that modification of this gene is detrimental for asexual blood stage development.

Overall, expression of each PP was highly variable, with localization falling into three major classes—localized/heterogeneous, nuclear, and cytoplasmic (Figures 2A and 2B), with a number falling into more than one class (nucleocytoplasmic). However, only two PPs were found to be localized to a single region throughout the entire life cycle: PPM4 (the nucleus) and NIF2 (the cytoplasm). Furthermore, a number were expressed only at specific stages. For example, PTP1 was found to be completely absent in male gametocytes but present at all other stages, whereas YVH1 was absent in ookinetes (Figure 2B).

Of the PPs localized to a precise region of the parasite body, PTP1 was found to be localized to the apical tip of mature ookinetes, suggestive of a role in apical polarity, whereas PPM5 was found to show both nuclear and membrane localization in zygotes but was mostly absent from the cytoplasm (Figure 2B).

Previous studies have localized a small number of *P. falciparum* PPs during asexual stages. PfPP2C (PPM2 in our study) has been shown to be highly expressed in rings, trophozoites, and schizonts (Mamoun et al., 1998), whereas PfYVH1 has been found to be active and undergo nucleocytoplasmic shuffling during periods of high transcriptional activity (Kumar et al., 2004), both of which are consistent with our study. Furthermore, PfYVH1 has been shown to interact with the cell-cycle-regulatory protein pescadillo (PES) (Kumar et al., 2004). However, PfPRL was localized to the ER and non-ER punctuate structures in *P. falciparum* asexual stages (Pendyala et al., 2008), whereas we found it exclusively in the zygotic cytoplasm, suggesting species-specific differences in PP localization and, perhaps, activity.

### Gene Disruption Analysis Suggests Nearly Half of the *P. berghei* PPs Are Redundant during Erythrocytic Stages

To identify PPs that are functional during *P. berghei* asexual blood stage development, we attempted to delete systematically each of the 30 *P. berghei PP*s using double homologous recombination (Figure S3A). A total of 178 individual gene deletion attempts were performed (at least 4 per gene; Table S1), resulting in successful deletion of 14 *PP*s (47%) as determined by detailed genotype analysis (Figures 2C and S3B; Table S1). Independent clones for each mutant were produced at least twice from independent experiments. Overall, we did not observe a reduced growth phenotype in mice for any of the mutants, suggesting redundancy for each during asexual blood stage development. As a result, we screened each mutant to see whether they are essential during sexual stage development and transmission in *Anopheles stephensi* mosquitoes.

### Phenotypic Analysis Suggests Six *P. berghei* PPs Are Required for Parasite Sexual Development/Sporogony and Differentiation in the Mosquito

To assess their role during sexual stage development, we initially screened the ability of the mutants to form viable ookinetes in vitro. A number of PKs are known to have an essential role at this stage, with CDPK4, MAP2, and SRPK being essential for microgamete production; NEK2, NEK4, PK7, and GAK being essential regulators of zygote development; and CDPK1 and CDPK3 required for ookinete gliding motility (Sebastian et al., 2012; Siden-Kiamos et al., 2006; Tewari et al., 2010).

For ten mutants, ookinete conversion showed no overt differences from wild-type parasites (Figure 2C; Table S2), suggesting no role for these PPs up to this stage. However, four mutants were strongly affected in their ability to produce viable ookinetes in vitro. Two of these, Δ*ppkl* and Δ*shlp1*, have been described previously and were shown to be essential for ookinete differentiation and oocyst development, respectively (Guttery et al., 2012; Patzewitz et al., 2013). One mutant, Δ*ppm1*, formed morphologically normal gametocytes (data not shown) and produced macrogametes that emerged from their host cells expressing the activation marker P28, but did not produce any ookinetes (Figure 2C; Table S2). Therefore, we assessed whether microgamete formation was affected in this mutant and found that exflagellation was completely blocked (Figure 2C; Table S2). Another mutant, Δ*ppm2*, showed grossly reduced macrogamete numbers (<30% of wild-type controls) and subsequent ookinete conversion (<5% of the population), with the majority of the population being retorts, suggesting this PP is essential for ookinete differentiation similar to PPKL (Figure 2C; Table S2). This was surprising, as the *P. falciparum* ortholog of PPM2 (PfPP2C) is refractory to deletion (Mamoun and Goldberg, 2001). One other mutant, Δ*ppm5*, produced viable ookinetes similar to wild-type controls, but was markedly reduced in its ability to produce fully formed oocysts (reduced in number and size with no sporozoite development) 14 days postinfection. Finally, Δ*ptpla* parasites produced equivalent numbers of oocysts compared to wild-type; however, no sporozoites were produced. The remaining eight mutants showed no observable defects throughout sexual development, sporogony, and transmission to mice (as assessed by blood smears 4–10 days postinfection) (Figure 2C; Table S2), suggesting functional redundancy for these PPs during these stages of the life cycle. However, the role of any PP during liver-stage development cannot be discounted, as we did not directly assess this stage of the life cycle in this study. In this study, all eight mutants gave rise to blood stage infection with a 4–5 day prepatent period in mosquito biteback experiments, suggesting little effect during liver stage development.

Systematic functional analysis of the *P. berghei* kinome revealed a number of PKs to be essential for parasite development in the mosquito (Tewari et al., 2010). In contrast to these kinases, which have diverged through numerous variations of the catalytic subunits, the PPs contain a comparatively small number of highly conserved catalytic subunits with supposedly nondiscriminatory substrate specificity in vitro. However, more recent studies have given the PPs far greater specificity due to their assembly with hundreds of regulatory subunits, giving them the ability to recognize environmental cues and coordinate highly complex and specific chains of events. Therefore, the refractory nature of the PPPs in this study is not surprising. They are known to form multimeric holoenzyme complexes with a wide variety of regulatory subunits that bestow substrate selectivity and direct subcellular localization of the catalytic subunit, which are highly regulated and specific (Virshup and Shenolikar, 2009). Similarly, the inability to delete three *FCP*s (NIF2, NIF3, and NIF4) is not surprising, as the common substrate of FCPs is the C-terminal domain of RNA polymerase II (Yeo et al., 2003). Finally, *yvh1* is essential in fungi (Sakumoto et al., 2001); therefore it is not surprising to find it refractory to deletion here. In contrast, the PPMs are generally thought to exist as monomers whose catalytic domains are highly conserved, and as we were able to successfully delete seven of nine (78%) of the *PPM*s, this suggests a mechanism of functional compensation by other PPMs.

We have shown here that, as expected, a number of PPs are essential for parasite sexual development/sporogony at similar stages to a number of PKs (Tewari et al., 2010). Whether they have similar substrates (or indeed target each other) is yet to be determined.

### Two Unique Plasmodial PPs Are Differentially Localized, Show Phosphatase Activity, and Are Phosphorylated and Myristoylated

Protein phosphorylation and *N*-myristoylation are important posttranslational mechanisms regulating protein function. PPM2 and PPM5 are suggested to be regulated by phosphorylation (Treeck et al., 2011), were recently identified as *N*-myristoylated in *P. falciparum* (Wright et al., 2014), and are shown here to have essential functions during sexual development. Therefore, we examined their transcription and expression profile, and *N*-myristoylation and phosphorylation status.

**Figure 3:**
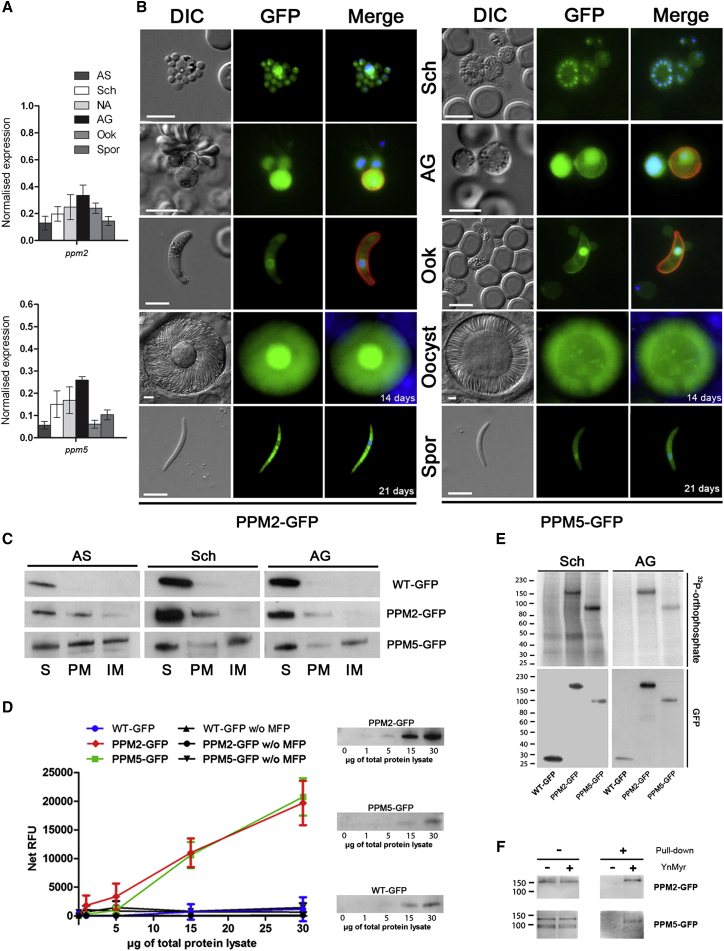
PPM2 and PPM5 Expression, Phosphatase Activity and Phosphorylation, and *N*-Myristoylation Status (A) Wild-type RNA expression of *ppm2* (upper panel) and *ppm5* (lower panel). Error bar ± SEM, n = 3. AS, asexual blood stages; Sch, schizonts; NA, nonactivated gametocytes; AG, activated gametocytes; Ook, ookinetes; Spor, sporozoites. (B) Expression of PPM2-GFP (left) and PPM5-GFP (right). Merge is the composite of Hoechst to detect the nuclei, GFP and Cy3 P28 for sexual stages. Scale bar, 5 μm. (C) Anti-GFP western blot of soluble (S), peripheral membrane (PM), and integral membrane (IM) fractions from parasite lysates. (D) (Left) Phosphatase activity in parasite lysate immunoprecipitates. Error bar ± SEM, n = 3. (Right) Anti-GFP western blot from corresponding lysates. (E) In vivo phosphorylation. (Upper panel) [^32^P]-phosphorylation of immunoprecipitated GFP-proteins from parasite lysates. (Lower panel) Corresponding western blot. Protein markers are to the left. (F) Parasite lysates labeled with YnMyr (+) and controls without labeling (−) were run directly (− pull-down) or following affinity purification (+) and detected with anti-GFP antibody. Protein markers are shown to the left. See also Figure S4 and Table S6.

qRT-PCR and C-terminal GFP tagging together demonstrated that both PPs are present throughout the life cycle, confirming previous studies (Hall et al., 2005; Le Roch et al., 2003) (Figures 3A and 3B). Subcellular fractionation of blood stage parasites confirmed a cytoplasmic and peripheral membrane localization for PPM2-GFP, whereas PPM5-GFP was present in all three fractions analyzed (Figure 3C), consistent with its diffuse localization in microscopic analyses (Figure 3B). PP activity assays using 3-O-methylfluorescein phosphate (MFP) as a substrate (Patzewitz et al., 2013) confirmed PPM2 and PPM5 to be active PPs (Figure 3D). Computational homology modeling of PPM2 and PPM5 confirms the presence of metal coordinating residues and showed the presence of long Asn-rich loops within the PP domain (Figure S4). Such Asn-rich low-complexity regions are often seen in *Plasmodium* proteins, and their function remains obscure. Although most *P. berghei* PPs do not have extensive Asn-rich regions, they occur in some other PPs, for example PPP8. The loops in PPM2 and PPM5 are atypical in that they are >100 residues and are located within the catalytic domain. For PPM2 and PPM5, the loop regions are located at different positions in the catalytic domain. However, PPM2 and PPM5 have one loop in common that protrudes close to the active site (Figure S4), and this loop is likely to affect interactions with substrates, and might be involved in substrate selection.

Analysis of PPM2-GFP and PPM5-GFP using ^32^P-orthophosphate labeling in vivo (Guttery et al., 2012) confirmed them to be phosphorylated in schizonts (as in *P. falciparum* [Treeck et al., 2011]) and activated gametocytes (Figure 3E), further implicating phosphorylation as a key regulator of PP activity (Mochida and Hunt, 2012). PPM2 and PPM5 proteins from schizonts were also labeled by the myristic acid analog tetradec-13-ynoic acid (YnMyr) (Heal et al., 2012) (Figure 3F), confirming previous *P. falciparum* myristome studies and highlighting these PPs as substrates of *N-*myristoyl transferase, which is currently being investigated as a potential drug target in malaria (Wright et al., 2014).

A myristoyl group is only a weak membrane anchor, allowing membrane association in a reversible and regulated manner, in agreement with the presence of PPM2 and PPM5 in soluble and membrane-associated fractions. PPM5 has a cluster of positive charges close to the N terminus (underlined residues in Figure S4F), and in other proteins, such as Src family kinases, these clusters are known to stabilize membrane anchoring of the protein. PPM2 lacks such a cluster, or other hydrophobic groups, and therefore membrane anchoring of PPM2 might be promoted through additional factors.

### PPM2 Is Essential for Gametocyte Sex Allocation and Ookinete Differentiation, whereas PPM5 Modulates Oocyst Development

**Figure 4:**
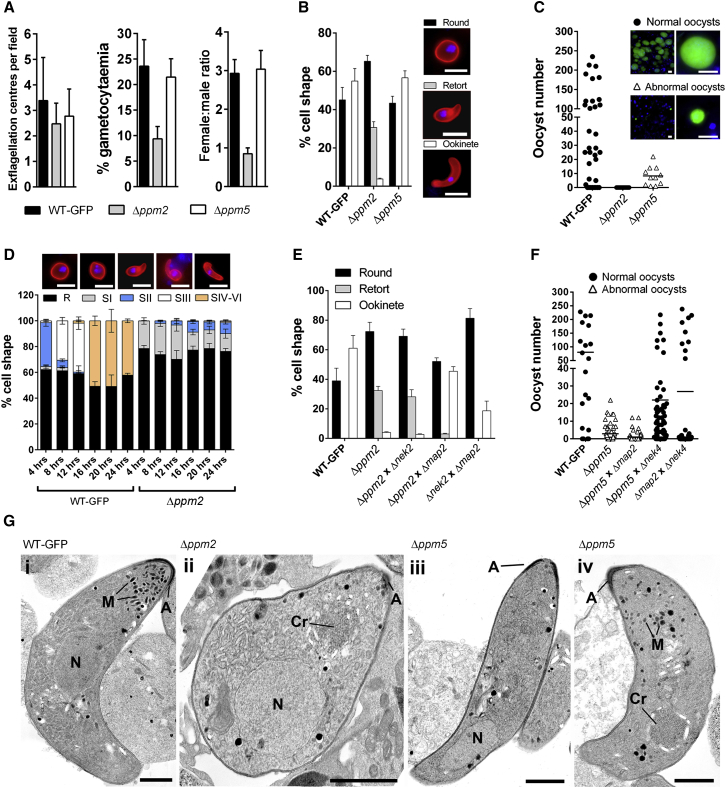
Phenotypic and Ultrastructure Analysis of Δ*ppm2* and Δ*ppm5* (A) Exflagellation (left), gametocytaemia (middle), and gametocyte sex allocation (right) of WT-GFP, Δ*ppm2*, and Δ*ppm5* parasites. Error bar ± SD; n = 3. (B) Ookinete conversion in WT-GFP, Δ*ppm2*, and Δ*ppm5* parasites. Error bar ± SD; n = 3. (C) Average number of oocysts per mosquito gut. Scale bar, arithmetic mean; n = 60. Infection prevalence was 81% for wild-type, 0% for Δ*ppm2*, and 88% for Δ*ppm5*. Scale bar, 50 μm. (D) Ookinete differentiation. Morphologies used for scoring are given above the graph and are previously described (Janse et al., 1985). Error bar ± SD, n = 3. Scale bar, 5 μm. (E) Ookinete conversion after genetic crossing. Error bar ± SD; n = 3. (F) Genetic complementation. Scale bar, arithmetic mean; n = 60. (G) (Gi) TEM of a longitudinal section through a wild-type crescent shaped ookinete. (Gii) Section through a Δ*ppm2* retort showing the bulbous shape of the parasite but with normal structures in the cytoplasm. Note the very few micronemes. (Giii) Example of a Δ*ppm5* ookinete showing the crescent shape but no micronemes. (Giv) Δ*ppm5* ookinete containing few micronemes. For all panels, A, apical membrane complex; N, nucleus; Cr, crystalline body; M, micronemes. Scale bar, 1 μm. See also Figure S5.

As PPM2 and PPM5 were identified as key regulators of sexual/sporogonic development, we performed an in-depth analysis to examine at what stage the proteins are essential. Although exflagellation in Δ*ppm2* was comparable to wild-type controls and Δ*ppm5* parasites (Figure 4A), gametocytaemia and subsequent female gamete production were severely reduced (Figure 4A). This reduced female gamete count was reflected in an altered female:male gametocyte ratio of approximately 1:1 in Δ*ppm2* parasites, compared to 3:1 observed in wild-type controls (Figure 4A). Sex allocation in *Plasmodium* is inherently female biased (∼3.6 female gametocytes to 1 male; Robert et al., 1996) in order to balance the greater number of male gametes per gametocyte, hence maximizing fertilization and transmission success. Furthermore, male gametocytes have been shown to have higher longevity in the blood meal (Reece et al., 2003) and are able to recognize genetically identical kin (Reece et al., 2008), both of which may affect sex ratios. Therefore, the reduction of macrogametes in vitro, altered sex allocation, and decreased infectivity in Δ*ppm2* parasites could suggest that the protein is vital for the response of *Plasmodium* parasites to environmental factors, or that longevity of the mutant female gametocytes is affected, resulting in lower numbers. Alternatively, PPM2 could play a role in recognition of genetically identical kin, with its absence resulting in a switch to a 1:1 ratio.

Zygote (ookinete) maturation in vitro was severely affected in Δ*ppm2* lines compared to wild-type, with the majority of the mutant population stalled by stage II of ookinete development, and oocyst development was completely ablated (Figures 4B and 4C). In contrast, Δ*ppm5* ookinetes developed normally and were motile (Figures 4B and S5A). However, oocyst development was severely affected, with reduced size (<40%) and numbers (<5%) compared to wild-type controls (Figures 4C and S5B) despite the fact that Δ*ppm5* ookinetes contained a normal DNA content (4N) similar to wild-type (Figure S5C). Analysis of Δ*ppm2* parasites revealed a reduced but highly variable DNA content (Figure S5C), suggesting meiotic DNA replication was initiated but aborted prior to completion, as observed previously in Δ*misfit* parasites (Bushell et al., 2009) and to some extent in *nek4* mutant parasites (Reininger et al., 2005). No sporozoites were present 14 days postinfection, and no transmission via biteback was observed (Table S2), confirming that this PP is essential during early oocyst development. Detailed analysis of ookinete differentiation showed that most of the Δ*ppm2* population did not progress to stage I, a few were arrested at stage II (Janse et al., 1985), and after 24 hr only a fraction of the population (<0.5%) had developed to stage VI, compared to >40% of wild-type controls (Figure 4D). This suggests multiple stages of essentiality for PPM2, i.e., sex allocation in developing gametocytes, and regulation of maturation, differentiation, and morphological development from stage II to III of ookinete development. The importance of this protein is strengthened by the fact that PfPP2C dephosphorylates translation elongation factor 1β (EF-1β) (Mamoun and Goldberg, 2001), with mutants of EF-1β showing severe growth defects and sensitivities to elongation inhibitors in yeast (Carr-Schmid et al., 1999).

### Δ*ppm2* and Δ*ppm5* Are Defective along the Female and Male Lineages, Respectively

In genetic crosses with lines deficient in either male (Δ*map2*) or female (Δ*nek2/*Δ*nek4*) gametes (Reininger et al., 2005, 2009; Tewari et al., 2005), Δ*map2* (but not Δ*nek2* gametes) formed mature ookinetes when crossed with Δ*ppm2* mutants (Figure 4E), indicating that PPM2 is necessary for female gamete formation, as seen for PPKL (Guttery et al., 2012). Crossing Δ*ppm5* with Δ*nek4* lines rescued the phenotype, with mature sporozoite-containing oocysts 14 days postinfection (Figure 4F), indicating the requirement for a functional *ppm5* gene in the male line. The essential requirement for a functional male lineage during oocyst development is similar to properties of the *misfit* gene, as is its stage of essentiality (Bushell et al., 2009). Whether or not MISFIT is a target of PPM5 requires further study.

### Ultrastructure Analysis Shows Defects in Ookinete Formation and Differentiation in Δ*ppm2* Mutants, whereas Δ*ppm5* Ookinetes Showed Partial Defects in Microneme Formation

We analyzed Δ*ppm2* gametocytes and stage-arrested ookinetes, and Δ*ppm5* ookinetes, using transmission electron microscopy (TEM). The Δ*ppm2* gametocyte samples contained fewer macrogametes, but the gametocyte (male and female) morphology was similar to that of wild-type (Figure S5D). In contrast to crescent-shaped wild-type ookinetes (Figure 4Gi), the vast majority of Δ*ppm2* ookinetes were arrested at the early retort stage II, with a bulbous shaped body and cytoskeletal abnormalities (Figure 4Gii). In the few ookinetes that did mature, the apical membrane complex appeared normal, and late-stage features such as the crystalline body were present, but there were few or no micronemes (Figure 4Gii), as seen in SHLP1 mutants (Patzewitz et al., 2013). Δ*ppm5* ookinetes were crescent shaped with the apical membrane complex seen in wild-type parasites (Figures 4Giii and 4Giv), but there was marked variability in the number of micronemes, ranging from normal to none (Figures 4Giii and 4Giv). This defect could explain the formation of only a small number of abnormal oocysts that are unable to produce sporozoites.

### Global Transcript Analysis of Δ*ppm2 and* Δ*ppm5* Reveals Dysregulation of Genes Involved in Reversible Phosphorylation, Membrane Structure, Motility, and Invasion

**Figure 5:**
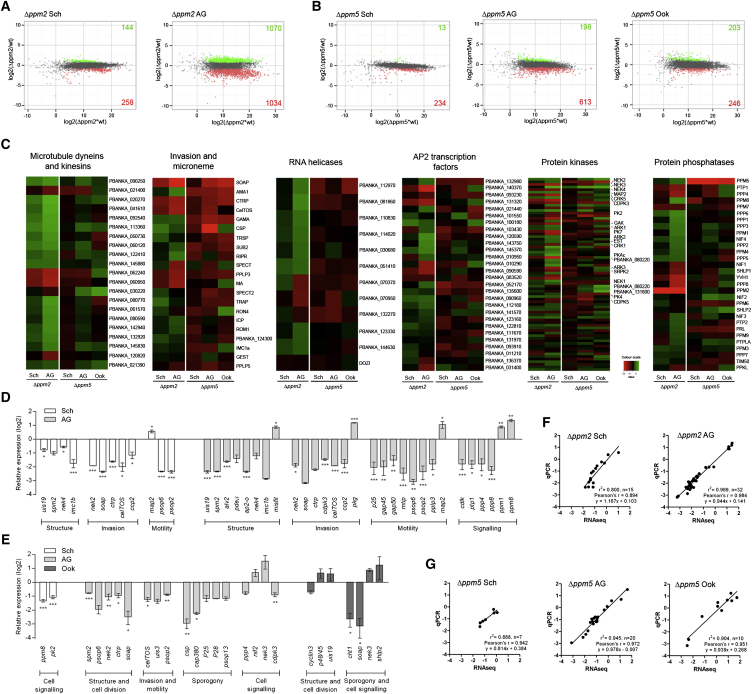
Global Transcriptional Analysis of Δ*ppm2* and Δ*ppm5* by RNA-Seq (A and B) Ratio-intensity scatterplots of normalized FPKM values for each stage of mutant development. Log_2_ fold change between wild-type and mutant (y axis) and the average FPKM value (x axis). (C) Log_2_ fold change in Δ*ppm2* and Δ*ppm5* at different life cycle stages. Functional groups were inferred from annotations available in GeneDB (http://www.genedb.org/Homepage). Genes are arranged in order of significance (in relation to regulation) in each sample and in the total data set. Full gene list and heatmap order are shown in Table S4. (D and E) qRT-PCR of a variety of genes (based on data from RNA-Seq) in (D) Δ*ppm2* and (E) Δ*ppm5* parasites compared to wild-type controls. Error bar ± SEM, n = 3 biological replicates. Schizonts, Sch; activated gametocytes, AG; ookinetes, Ook. Student’s t test, ^∗^p < 0.1, ^∗∗^p < 0.05, ^∗∗∗^p < 0.001. (F and G) qRT-PCR validation of the RNA-Seq data using Log_2_ values. See also Figure S6, Table S3, Table S4, and Table S6.

Transcription was analyzed by strand-specific RNA-sequencing (RNA-Seq) across relevant life cycle stages, and identified changes in key genes involved in zygote/ookinete development and transmission when the Δ*ppm2* and Δ*ppm5* lines were compared to wild-type (Figures 5A and 5B; Table S3 and Table S4).

We analyzed transcription in two Δ*ppm2* life stages, with particular emphasis on genes related to zygote/ookinete structure, motility, and invasion. Several gene clusters significantly affected in Δ*ppm2* schizonts and activated gametocytes were identified (Figure 5C; Table S4), which may be responsible for the pleiotropic nature of the Δ*ppm2* phenotype. Changes included downregulation of genes important for membrane structure (*kinesins*, *dyneins* [Fowler et al., 2001], *imc1b* [Tremp et al., 2008]), cell-cycle regulation (*pdeδ* [Moon et al., 2009] and *nek4* [Reininger et al., 2005]), ookinete motility (*gap45* and *gap50* [Baum et al., 2006]), and invasion (*soap*, *ctrp*, and *cdpk3* [Angrisano et al., 2012]) (Figures 5C and 5D; Table S3 and Table S4). Members the AP2 transcription factor family implicated in gametocyte and zygote formation (including *ap2-o*) (Painter et al., 2011; Yuda et al., 2009) were also differentially regulated (Figures 5C and 5D; Table S3 and Table S4). We also observed a general increase of RNA helicases in activated gametocytes. Furthermore, the few mature ookinetes that did form had reduced *gap50* and *soap* expression, potentially limiting motility, invasion, and oocyst formation (Figure 5D; Table S3). Global Gene Ontology (GO) analysis revealed enrichment of multiple categories such as “microtubule cytoskeleton” and “microtubule-based movement” in Δ*ppm2* lines (Figures 5C, 5D, and S6A; Table S3 and Table S4).

We analyzed transcription in three developmental stages (schizonts, activated gametocytes, and ookinetes) of Δ*ppm5* parasites compared to wild-type. Although the Δ*ppm5* phenotype was first observed in oocysts (Figure 4C), genetic crossing indicated that the mutation affects the male gametocyte (Figure 4F). This is supported by the RNA-Seq analysis, since several gene clusters related to the Δ*ppm5* phenotype were differentially expressed (Figure 5C; Table S4). Genes associated with microneme development (*soap* and *ctrp*), oocyst development and sporogony (*cap380* [Srinivasan et al., 2008] and *csp* [Ménard et al., 1997]) were downregulated in activated Δ*ppm5* gametocytes (Figures 5C and 5E; Table S3). GO analysis revealed enrichment of “microneme,” “cytoplasm,” and “apical part of cell” in all stages of Δ*ppm5* analyzed (Figure S6A), corresponding well with the phenotype.

In activated Δ*ppm2* gametocytes, 39 PKs, including ones essential for zygote development (*nek2*, *nek4*, *pk7*, and *gak*) and ookinete motility (*cdpk3*) (Tewari et al., 2010), were differentially expressed, whereas 17 PPs, including those associated with ookinete development such as *shlp1*, were differentially expressed (Figures 5C and 5D; Table S4), suggesting PPM2 has a signaling role in female development. In contrast, only a few PKs (such as *nek2* and *nek3*) and PPs (*ppm6* and *ppm8*) were differentially regulated in activated Δ*ppm5* gametocytes.

For both mutants, the RNA-Seq data were validated using qRT-PCR for selected genes (Figures 5D and 5E) and showed a good correlation (Figures 5F and 5G). It should be noted that transcriptional changes in the mutants probably reflect phenotypic changes in the cell state at a given life cycle stage, and cannot be used to infer a direct function of the PP, owing to the likely complexity of the signaling pathways.

To provide additional support for the differential gene expression patterns observed in Δ*ppm2* and Δ*ppm5* PP mutants, we compared protein-protein interaction partners of PPM2 and PPM5 derived from growth perturbation data sets of *P*. *falciparum* (Hu et al., 2010) and the differentially expressed genes seen in Δ*ppm2* and Δ*ppm5* across the studied life stages (Table S3). We provide evidence for significant numbers of differentially expressed genes in Δ*ppm2* and Δ*ppm5* mutants directly interacting with PPM2 and PPM5, respectively (Figure S6C). Approximately 30% (top 50% network, p value = 0.0002) of the differentially expressed genes in Δ*ppm2* schizonts and activated gametocytes were also found to directly interact with PPM2 (Figures S6C and S6D; Table S3). Several ookinete motility proteins and PKs (CDPK3 and PK7) were all found to interact directly with PPM2 (Figure S6).

In Δ*ppm5*, approximately 28% (top 50% network, p value = 0.002) of the differentially expressed genes in the three developmental stages of Δ*ppm5* were found to directly interact with PPM5 (Figures S6B–S6D; Table S3). For example, the data suggest that CDPK3, CDPK5, and SHLP2 interact directly with PPM5. Finally, it is important to note that while our network analysis indicates to an extent that the transcriptional changes we observe are related to the knocked out PP, the precise nature of these interactions remains to be elucidated experimentally.

### Conclusions

**Figure 6:**
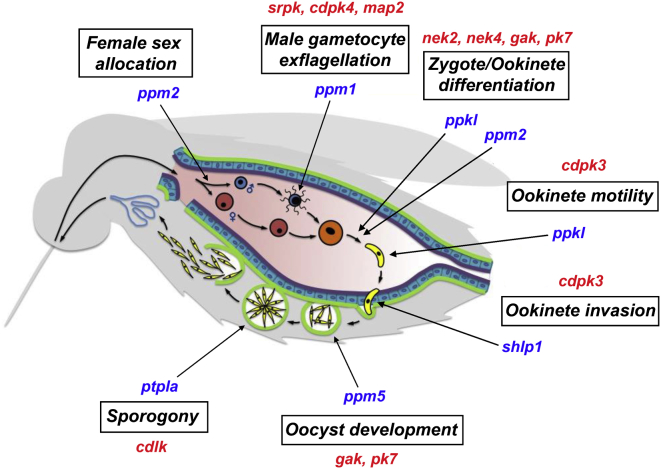
Summary of PP Function throughout the *P. berghei* Life Cycle PPs with essential functions in the mosquito are highlighted (blue). Protein kinases essential at similar stages (Tewari et al., 2010) are highlighted in red. See also Table S2.

This systematic functional analysis of the *P. berghei* phosphatome shows that PPs have many essential functions during the *Plasmodium* life cycle (particularly during sexual and sporogonic development), as found with the plasmodial kinome (Tewari et al., 2010) and summarized in Figure 6. In contrast to the PKs, which have diverged through evolution of the catalytic subunits, PPs comprise a small number of highly conserved catalytic subunit families. Specificity is conferred in part by association with many regulatory subunits, allowing PPs to respond to environmental cues and coordinate complex and specific chains of events (Virshup and Shenolikar, 2009). It is well known that signal transduction pathways are not simply “switched on” by kinases and “switched off” by PPs; rather, it is often a dynamic balance between the two, with multiple kinases and PPs contributing to certain stage-specific pathways. A number of PPs were shown here to be essential at similar stages of the life cycle to certain kinases (e.g., MAP2 and PPM1; PK7 and PPM5; see summary, Figure 6), with transcriptome sequencing further demonstrating that reversible protein phosphorylation is likely to be a complex and highly coordinated process that regulates sexual and sporogonic development. Whether these kinases and PPs truly act in a complementary manner and whether or not changes in transcription profiles are well downstream of the point at which these enzymes act will be determined in future studies.

As nearly half of the PP genes are nonessential for *P. berghei* blood stages, a large part of the phosphatome can be deprioritised as targets for drugs directed against this life cycle stage. Most drug discovery studies in mammalian systems have focused on PTPs with varying success (De Munter et al., 2013), although there are studies (Moorhead et al., 2007) proposing small molecule inhibitors of PPs to target diabetes, obesity, and other human diseases (De Munter et al., 2013; Zhang et al., 2013). There is also great interest in understanding the role of PPs as regulators of the cell cycle and mitosis, and hence as targets for cancer therapy (Barr et al., 2011; Mochida and Hunt, 2012). Overall, protein PPs with unique roles in disease may be promising targets for therapeutic intervention, and, following this study, various multifunctional approaches can be used in the future to identify the potential of unique malarial PPs as part of a drug discovery program.

## Experimental Procedures

### Ethics Statement

All animal work has passed an ethical review process and was approved by the United Kingdom Home Office. The project license number is 40/3344.

### Animals

Six- to eight-week-old female Tuck-Ordinary (TO) outbred mice (Harlan) were used for all experiments.

### Comparative Bioinformatics

PPs from a variety of families were identified in the predicted proteomes of *P. berghei*, *P. falciparum*, and 44 other diverse eukaryotes using HMMER3. For classification, full-length proteins were clustered and large-scale maximum-likelihood phylogenies built from alignments of PP domains (MAFFT6.24) with statistical support from the approximate Likelihood Ratio Test as implemented by PhyML3.0. Possible myristoylation was predicted using *N*-Myristoyltransferase (NMT) Myristoylator (Bologna et al., 2004).

### C-Terminal GFP Fusion and Gene Deletion

To C-terminally tag PPs with GFP, a single homologous recombination strategy at the endogenous locus was used (Guttery et al., 2012). For systematic gene deletion analyses, a traditional double homologous recombination approach was used (Tewari et al., 2010). Successful integration of target sequences was confirmed using genotype analysis. For each mutant, at least two clones were produced from independent transfections (see Table S5 for primer sequences).

### Phenotypic Analysis

Phenotypic analyses were performed as previously described (Guttery et al., 2012; Tewari et al., 2010). Asexual proliferation and gametocytogenesis were analyzed using blood smears. Gametocyte activation, zygote formation, and ookinete conversion rates were monitored using in vitro cultures and the surface antigen P28. For mosquito transmission, triplicate sets of 20–60 *Anopheles stephensi* were used.

### PPM2 and PPM5 Phosphatase Assay, Subcellular Fractionation and In Vivo Phosphorylation

Blood aliquots from parasite-infected mice were processed for protein purification and PP assay. Soluble (hypotonic lysis), peripheral membrane (carbonate soluble), and integral membrane (carbonate insoluble) fractions were analyzed by western blotting for subcellular fractionation. For in vivo phosphorylation, purified activated gametocytes and purified schizonts were metabolically labeled with 3–5 MBq ^32^P-orthophosphate for 30 min and processed for phosphorylation assay (Patzewitz et al., 2013).

### Metabolic Labeling and Purification of *N*-Myristoylated Proteins

In vivo myristoylation of PPM2 and PPM5 was assessed using the alkyne-tagged myristic-acid analog YnMyr and bio-orthogonal ligation to label all proteins that had incorporated the YnMyr probe with a biotinylated capture reagent ligated to the alkyne tag, which then allowed the selective affinity pull-down of tagged proteins with a streptavidin-conjugated resin (Wright et al., 2014).

### Transcriptome Sequencing, qRT-PCR, and Interaction with Phosphatase Network Analysis

qRT-PCR was performed from 250 ng of total RNA using *hsp70* and *seryl-tRNA synthetase* as references (see Table S6 for primer sequences). Strand-specific mRNA sequencing was performed from two to four biological replicates of total RNA using TruSeq Stranded mRNA Sample Prep Kit LT (Illumina). Libraries were sequenced in Illumina Hiseq with paired-end 100 bp read chemistry. Strand-specific RNA-Seq paired-end reads were mapped onto the *P. berghei* ANKA genome (PlasmoDB-9.2) using TopHat version 2.0.8 (Trapnell et al., 2009), then quantified and compared across different samples using Cuffdiff version 2.1 (Trapnell et al., 2013). Differentially expressed genes from Δ*ppm2* and Δ*ppm5* were overlapped with interaction partners for both PP networks derived previously in *P. falciparum* (Hu et al., 2010).

### Statistical Analyses

Statistical analyses were performed using GraphPad Prism (GraphPad Software). For relative gene expression, a Student’s t test was used.

A complete description of the materials and methods used in this study is provided in the Supplemental Experimental Procedures.

## Author Contributions

R.T. and A.A.H. conceived of and designed the study. R.T., D.S.G., B.P., D.B., R.J.W., S.W., E.-M.P., and U.S. performed the functional analysis. B.W. performed the phylogenetic analyses. D.J.P.F. performed the TEM experiments. A. Ramaprasad, A.M.A.H.M., and A.P. provided the RNA-Seq and protein interactome data analysis. A. Radhakrishnan and S.T.A. performed the computational structural analyses. M.H.W. and E.W.T. performed the myristoylation assays. D.S.G., B.P., D.J.P.F., A. Ramaprasad, A.P., A.M.A.H.M., B.W., R.J.W., A.A.H., and R.T. analyzed the data. D.S.G., A.A.H., and R.T. wrote the manuscript, and all others corrected and contributed to it.

## Accession Numbers

Transgenic parasites lines generated in the study are deposited to the Rodent malaria database at http://www.pberghei.eu/.

## Supplemental Information

Document S1. Six Figures and Supplemental Experimental Procedures

Table S1. Proposed Protein Phosphatase Gene Names, C-Terminal GFP Fusion of Endogenous PPs, and Summary of Deletion Attempts Total number of C-terminal GFP fusion transfection and gene knockout attempts and how many led to successful tagging or successful deletion of the target as confirmed by genotypic analysis. We were unable to successfully tag PPM9. p value gives an estimate for the likelihood of error for “possibly essential” genes; it is calculated from the average technical failure rate (0.547 per attempt for redundant genes in this study) to the power of n, where n is the total number of deletion attempts for the gene. Related to Figure 2.

Table S2. Phenotypic Analysis of Deletion Mutants Raw data from phenotyping analysis of 14 mutants at different development stages. All data are given as a percentage of wild-type controls studied in parallel. SD, standard deviation. n, number of replicate experiments. Related to Figure 2.

Table S3. Significantly Altered Gene Expression in Ä*ppm2* and Ä*ppm5* Mutants Compared to Wild-Type and Putative Interactions in Phosphatase Networks Raw differential expression, log2 fold change values, and putative interactions in phosphatase networks of significantly altered genes in Ä*ppm2* and Ä*ppm5* mutants. Gene names were obtained from GeneDB. Related to Figure 5.

Table S4. Heatmap Clusters and log2 Ratios of Gene Expression Differential expression (log2 fold change; Table S3) values used to produce the heatmaps in Figure 5C of protein phosphatases, protein kinases, RNA helicases, AP2 transcription factors, host invasion- and microneme-related proteins, microtubule-/axoneme-related dyneins and kinesins, and enzymes involved in glycolysis (BIR proteins; not included in Figure 5C), in Ä*ppm2* and Ä*ppm5* at schizont, activated gametocyte, and ookinete life stages. Cells highlighted in green were upregulated; cells highlighted in red were downregulated. Sch, schizonts; AG, activated gametocytes; Ook, ookinetes. Related to Figure 5.

Table S5. Primers Used for Generation of C-Terminal GFP Fusion, Gene Deletion Constructs, and Genotype Analysis Generic sequences for KpnI and ApaI restriction sites, used for GFP fusion cloning purposes. ol492 sequence is given in Guttery et al. (2012). Generic sequences for ApaI/HindIII and EcoRI and XbaI restriction sites, used for gene deletion cloning purposes. ol248 and ol539 sequences are given in Tewari et al. (2010). Related to Figure 2.

Table S6. Primers Used for qRT-PCR Sequences shown are written 5′–3′. Related to Figure 3.

Document S2. Article plus Supplemental Information
