# Supplementary material for: Genome-wide Functional Analysis of Plasmodium Protein Phosphatases Reveals Key Regulators of Parasite Development and Differentiation
Source: Cell Host Microbe. 2014 Jul 9;16(1):128–40. doi: 10.1016/j.chom.2014.05.020 (PMC4094981; doi:10.1016/j.chom.2014.05.020)
Supplement: Document S1. Six Figures and Supplemental Experimental Procedures [file mmc1.pdf]

Cell Host & Microbe, Volume *16*

Supplemental Information

Genome-wide Functional Analysis of *Plasmodium* Protein Phosphatases Reveals Key Regulators of Parasite Development and Differentiation

David S. Guttery, Benoit Poulin, Abhinay Ramaprasad, Richard J. Wall, David J.P. Ferguson, Declan Brady, Eva-Maria Patzewitz, Sarah Whipple, Ursula Straschil, Megan H. Wright, Alyaa M.A.H. Mohamed, Anand Radhakrishnan, Stefan T. Arold, Edward W. Tate, Anthony A. Holder, Bill Wickstead, Arnab Pain, and Rita Tewari

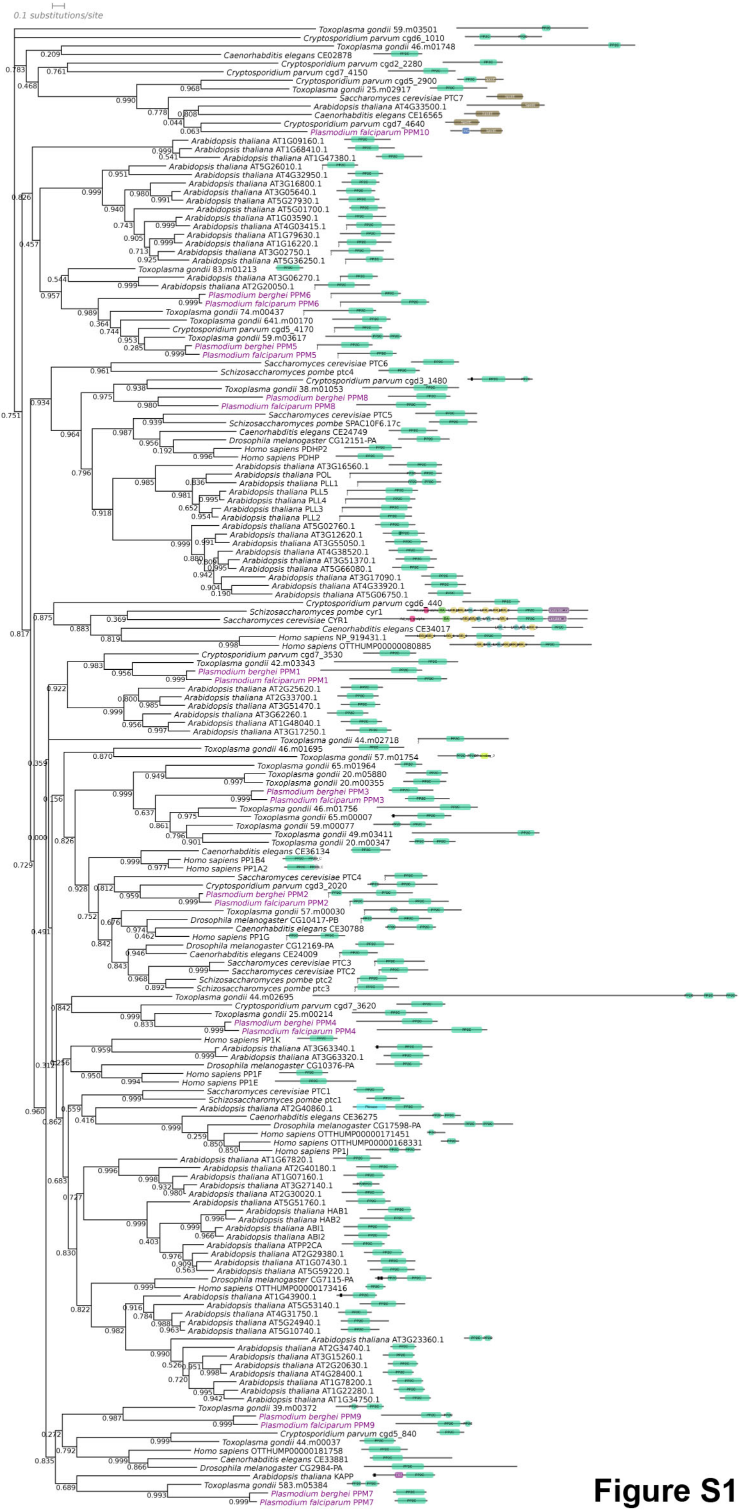
0.1 substitutions/site
Toxoplasma gondii 59.m03501
Cryptosporidium parvum cgd6_1010
Toxoplasma gondii 46.m01748
Caenorhabditis elegans CE02878
Cryptosporidium parvum cgd2_2280
Cryptosporidium parvum cgd7_4150
Cryptosporidium parvum cgd5_2900
Toxoplasma gondii 25.m02917
Saccharomyces cerevisiae PTC7
Arabidopsis thaliana AT4G33500.1
Caenorhabditis elegans CE16565
Cryptosporidium parvum cgd7_4640
Plasmodium falciparum PPM10
Arabidopsis thaliana AT1G09160.1
Arabidopsis thaliana AT1G68410.1
Arabidopsis thaliana AT1G47380.1
Arabidopsis thaliana AT5G26010.1
Arabidopsis thaliana AT4G32950.1
Arabidopsis thaliana AT3G16800.1
Arabidopsis thaliana AT3G05640.1
Arabidopsis thaliana AT5G27930.1
Arabidopsis thaliana AT5G01700.1
Arabidopsis thaliana AT1G03590.1
Arabidopsis thaliana AT4G03415.1
Arabidopsis thaliana AT1G79630.1
Arabidopsis thaliana AT1G16220.1
Arabidopsis thaliana AT3G02750.1
Arabidopsis thaliana AT5G36250.1
Toxoplasma gondii 83.m01213
Arabidopsis thaliana AT3G06270.1
Arabidopsis thaliana AT2G20050.1
Plasmodium berghei PPM6
Plasmodium falciparum PPM6
Toxoplasma gondii 74.m00437
Toxoplasma gondii 641.m00170
Cryptosporidium parvum cgd5_4170
Toxoplasma gondii 59.m03617
Plasmodium berghei PPM5
Plasmodium falciparum PPM5
Saccharomyces cerevisiae PTC6
Schizosaccharomyces pombe ptc4
Cryptosporidium parvum cgd3_1480
Toxoplasma gondii 38.m01053
Plasmodium berghei PPM8
Plasmodium falciparum PPM8
Saccharomyces cerevisiae PTC5
Schizosaccharomyces pombe SPAC10F6.17c
Caenorhabditis elegans CE24749
Drosophila melanogaster CG12151-PA
Homo sapiens PDHP2
Homo sapiens PDHP
Arabidopsis thaliana AT3G16560.1
Arabidopsis thaliana POL
Arabidopsis thaliana PLL1
Arabidopsis thaliana PLL5
Arabidopsis thaliana PLL4
Arabidopsis thaliana PLL3
Arabidopsis thaliana PLL2
Arabidopsis thaliana AT5G02760.1
Arabidopsis thaliana AT3G12620.1
Arabidopsis thaliana AT3G55050.1
Arabidopsis thaliana AT4G38520.1
Arabidopsis thaliana AT3G51370.1
Arabidopsis thaliana AT5G66080.1
Arabidopsis thaliana AT3G17090.1
Arabidopsis thaliana AT4G33920.1
Arabidopsis thaliana AT5G06750.1
Cryptosporidium parvum cgd6_440
Schizosaccharomyces pombe cyr1
Saccharomyces cerevisiae CYR1
Caenorhabditis elegans CE34017
Homo sapiens NP_919431.1
Homo sapiens OTTHUMP00000080885
Cryptosporidium parvum cgd7_3530
Toxoplasma gondii 42.m03343
Plasmodium berghei PPM1
Plasmodium falciparum PPM1
Arabidopsis thaliana AT2G25620.1
Arabidopsis thaliana AT2G33700.1
Arabidopsis thaliana AT3G51470.1
Arabidopsis thaliana AT3G62260.1
Arabidopsis thaliana AT1G48040.1
Arabidopsis thaliana AT3G17250.1
Toxoplasma gondii 44.m02718
Toxoplasma gondii 46.m01695
Toxoplasma gondii 57.m01754
Toxoplasma gondii 65.m01964
Toxoplasma gondii 20.m05880
Toxoplasma gondii 20.m00355
Plasmodium berghei PPM3
Plasmodium falciparum PPM3
Toxoplasma gondii 46.m01756
Toxoplasma gondii 65.m00007
Toxoplasma gondii 59.m00077
Toxoplasma gondii 49.m03411
Toxoplasma gondii 20.m00347
Caenorhabditis elegans CE36134
Homo sapiens PP1B4
Homo sapiens PP1A2
Saccharomyces cerevisiae PTC4
Cryptosporidium parvum cgd3_2020
Plasmodium berghei PPM2
Plasmodium falciparum PPM2
Toxoplasma gondii 57.m00030
Drosophila melanogaster CG10417-PB
Caenorhabditis elegans CE30788
Homo sapiens PP1G
Drosophila melanogaster CG12169-PA
Caenorhabditis elegans CE24009
Saccharomyces cerevisiae PTC3
Saccharomyces cerevisiae PTC2
Schizosaccharomyces pombe ptc2
Schizosaccharomyces pombe ptc3
Toxoplasma gondii 44.m02695
Cryptosporidium parvum cgd7_3620
Toxoplasma gondii 25.m00214
Plasmodium berghei PPM4
Plasmodium falciparum PPM4
Homo sapiens PP1K
Arabidopsis thaliana AT3G63340.1
Arabidopsis thaliana AT3G63320.1
Drosophila melanogaster CG10376-PA
Homo sapiens PP1F
Homo sapiens PP1E
Saccharomyces cerevisiae PTC1
Schizosaccharomyces pombe ptc1
Arabidopsis thaliana AT2G40860.1
Caenorhabditis elegans CE36275
Drosophila melanogaster CG17598-PA
Homo sapiens OTTHUMP00000171451
Homo sapiens OTTHUMP00000168331
Homo sapiens PP1J
Arabidopsis thaliana AT1G67820.1
Arabidopsis thaliana AT2G40180.1
Arabidopsis thaliana AT1G07160.1
Arabidopsis thaliana AT3G27140.1
Arabidopsis thaliana AT2G30020.1
Arabidopsis thaliana AT5G51760.1
Arabidopsis thaliana HAB1
Arabidopsis thaliana HAB2
Arabidopsis thaliana ABI1
Arabidopsis thaliana ABI2
Arabidopsis thaliana ATPP2CA
Arabidopsis thaliana AT2G29380.1
Arabidopsis thaliana AT1G07430.1
Arabidopsis thaliana AT5G59220.1
Drosophila melanogaster CG7115-PA
Homo sapiens OTTHUMP00000173416
Arabidopsis thaliana AT1G43900.1
Arabidopsis thaliana AT5G53140.1
Arabidopsis thaliana AT4G31750.1
Arabidopsis thaliana AT5G24940.1
Arabidopsis thaliana AT5G10740.1
Arabidopsis thaliana AT3G23360.1
Arabidopsis thaliana AT2G34740.1
Arabidopsis thaliana AT3G15260.1
Arabidopsis thaliana AT2G20630.1
Arabidopsis thaliana AT4G28400.1
Arabidopsis thaliana AT1G78200.1
Arabidopsis thaliana AT1G22280.1
Arabidopsis thaliana AT1G34750.1
Toxoplasma gondii 39.m00372
Plasmodium berghei PPM9
Plasmodium falciparum PPM9
Cryptosporidium parvum cgd5_840
Toxoplasma gondii 44.m00037
Homo sapiens OTTHUMP00000181758
Caenorhabditis elegans CE33881
Drosophila melanogaster CG2984-PA
Arabidopsis thaliana KAPP
Toxoplasma gondii 583.m05384
Plasmodium berghei PPM7
Plasmodium falciparum PPM7

Figure S1

Figure S1, related to Figure 1: Phylogenetic analysis of the PPMs

A maximum likelihood tree inferred from 288 alignable residues is shown with topology support from the minimum of parametric χ^2-based or Shimodaira-Hasegawa-like tests. All PPM phosphatases identified in the predicted proteomes of *Plasmodium berghei*, *Plasmodium falciparum* and also *Arabidopsis thaliana*, *Caenorhabditis elegans*, *Cryptosporidium parvum*, *Drosophila melanogaster*, *Homo sapiens*, *Saccharomyces cerevisiae*, *Schizosaccharomyces pombe* and *Toxoplasma gondii*, were trimmed to the conserved phosphatase region, aligned and highly similar sequences were removed. Tree and topology support were inferred using PhyML-aLRT (http://www.atgc-montpellier.fr/phyml/alrt/) with the WAG substitution matrix and a gamma-distributed variation in substitution rate approximated to 4 discrete categories (shape parameter estimated from the data). Protein domain architectures were predicted as described in Supplemental Experimental Procedures.

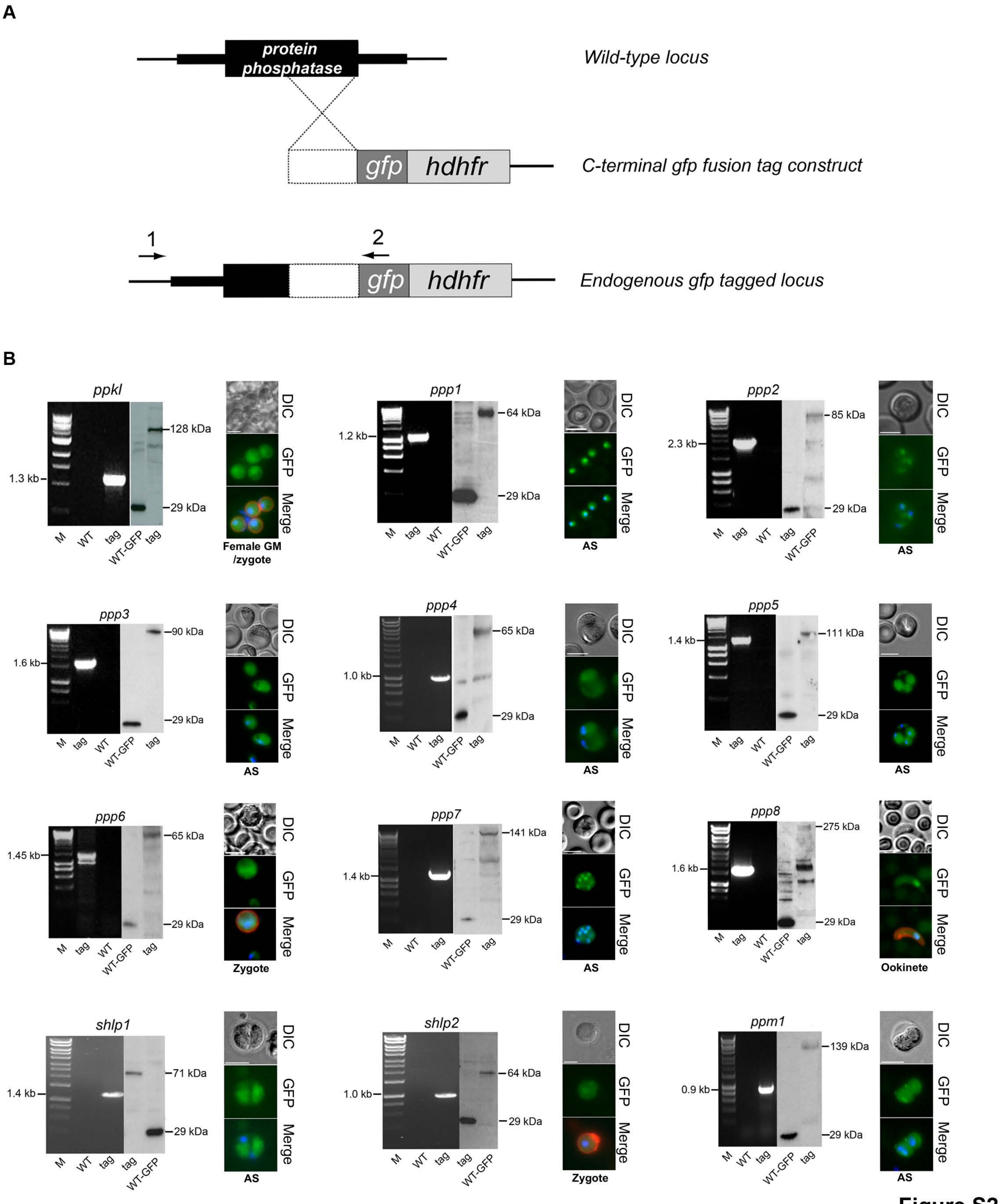
A
protein phosphatase
Wild-type locus
gfp
hdhfr
C-terminal gfp fusion tag construct
1
2
Endogenous gfp tagged locus
B
ppkl
1.3 kb
128 kDa
29 kDa
M
WT
tag
WT-GFP
tag
DIC
GFP
Merge
Female GM /zygote
ppp1
1.2 kb
64 kDa
29 kDa
AS
ppp2
2.3 kb
85 kDa
29 kDa
AS
ppp3
1.6 kb
90 kDa
29 kDa
AS
ppp4
1.0 kb
65 kDa
29 kDa
AS
ppp5
1.4 kb
111 kDa
29 kDa
AS
ppp6
1.45 kb
65 kDa
29 kDa
Zygote
ppp7
1.4 kb
141 kDa
29 kDa
AS
ppp8
1.6 kb
275 kDa
29 kDa
Ookinete
shlp1
1.4 kb
71 kDa
29 kDa
AS
shlp2
1.0 kb
64 kDa
29 kDa
Zygote
ppm1
0.9 kb
139 kDa
29 kDa
AS
Figure S2

B (cont.)

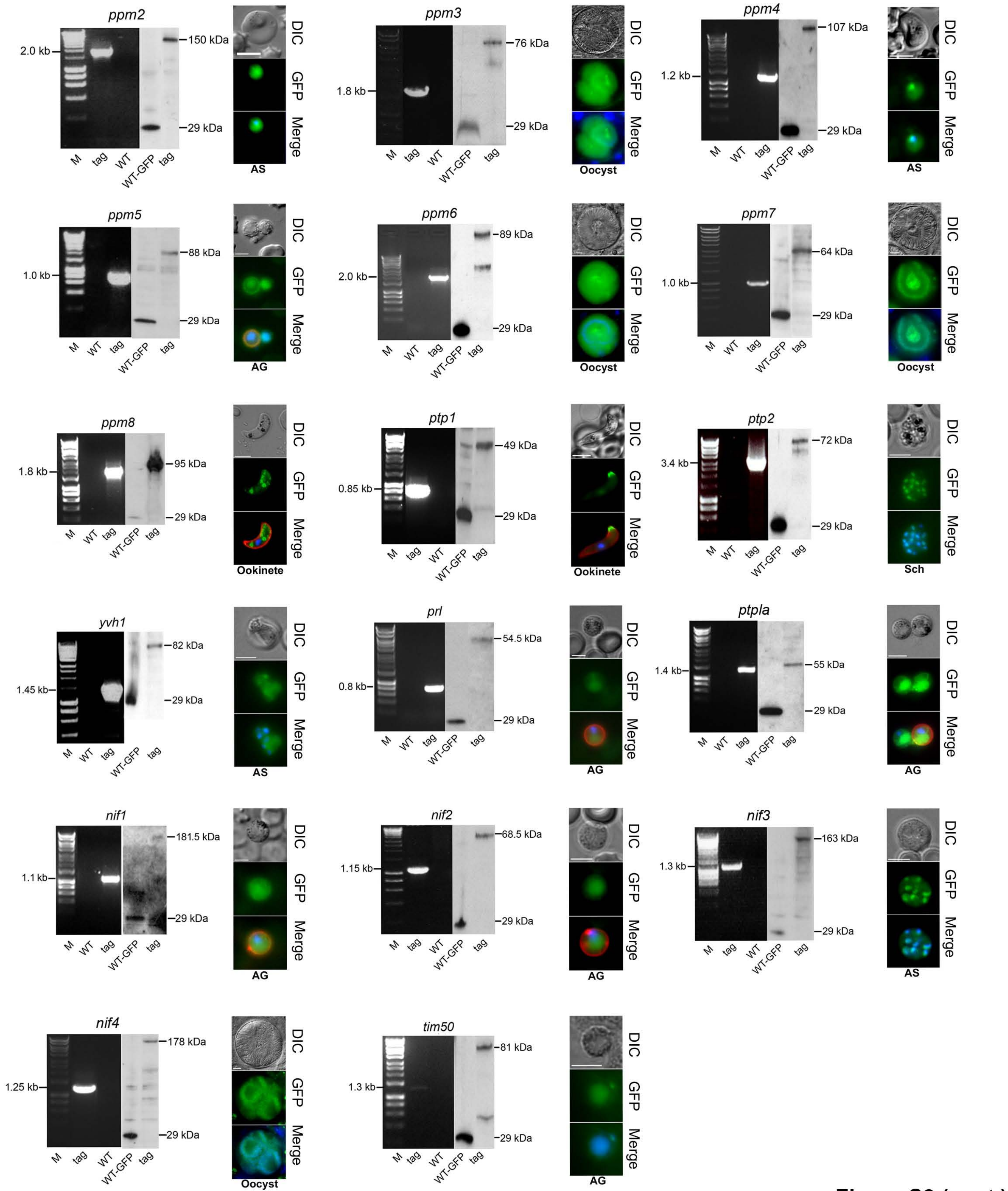

Figure S2 (cont.)

Figure S2, related to Figure 2: *gfp* tagging of the PPs and representative expression

(A) Schematic representation for 3'-tagging of each endogenous *P. berghei* PP gene with *gfp* via single homologous recombination. Primers 1+2 used for diagnostic PCR are indicated. (B) For each PP are shown: (left) diagnostic integration PCR showing band at the expected size, confirming successful integration of the tagging construct; (middle) Western blot analysis using an anti-GFP antibody against control GFP (WT-GFP) and transgenic (tag) parasite protein showing bands of 29 kDa for GFP and of the expected size for the corresponding PP-GFP; (right) images showing expression of the GFP tagged PP in one of the representative stages of the life cycle: (asexual blood stages (AS), schizonts (Sch), activated gametocytes (AG), ookinete (Ook) and oocyst). A Cy3-conjugated antibody recognising P28 on the surface of activated female gametocytes, zygotes, and ookinetes was used for the sexual stages, and the cells were displayed by differential interference contrast (DIC). Merge is the composite image of Hoechst dye to detect the nuclei, GFP, and P28 for the sexual stages. Bar = 5 µm.

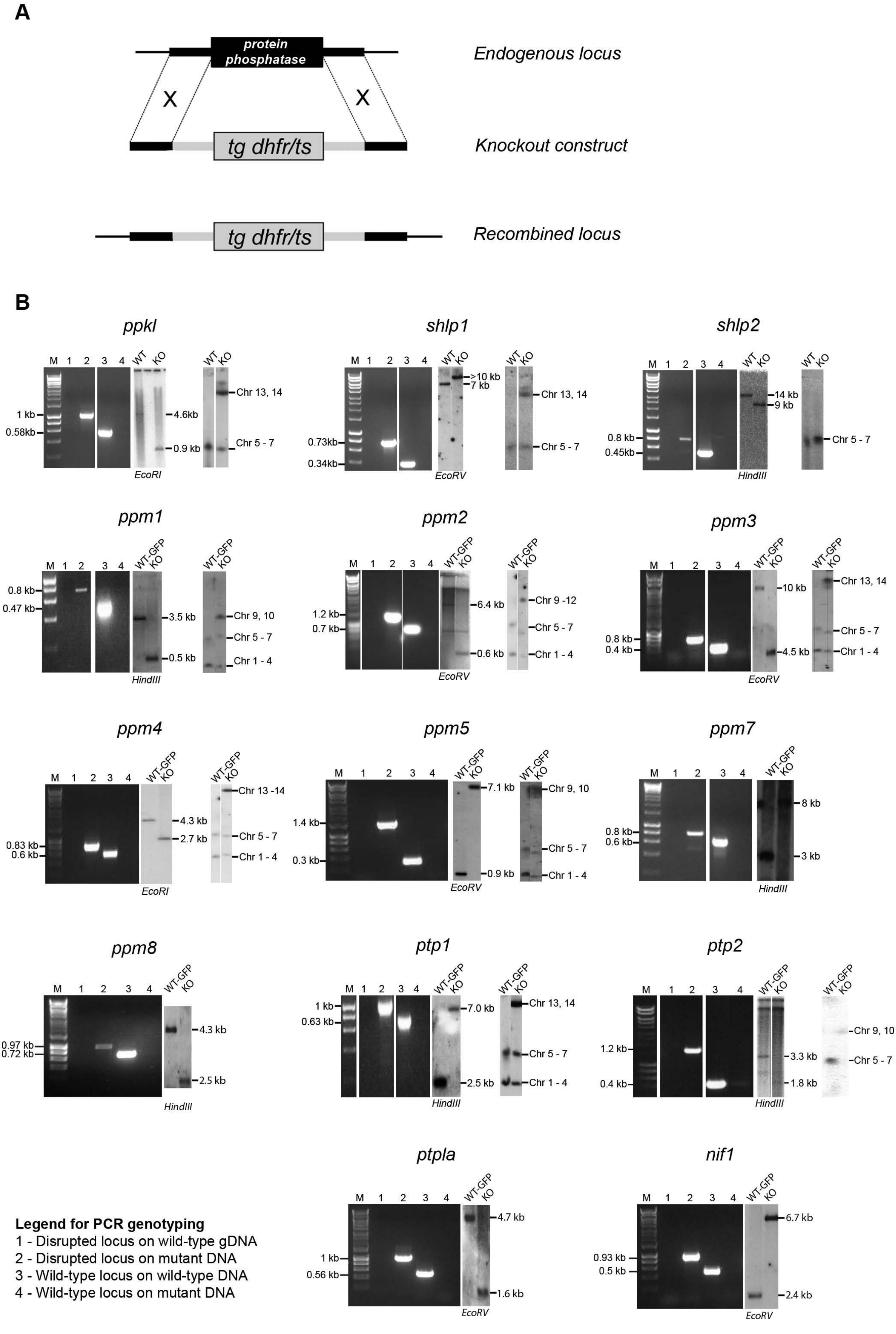
A
protein phosphatase
X
X
Endogenous locus
tg dhfr/ts
Knockout construct
tg dhfr/ts
Recombined locus
B
ppkl
M 1 2 3 4 WT KO WT KO
1 kb
0.58kb
4.6kb
0.9 kb
Chr 13, 14
Chr 5 - 7
EcoRI
shlp1
M 1 2 3 4 WT KO WT KO
>10 kb
7 kb
0.73kb
0.34kb
Chr 13, 14
Chr 5 - 7
EcoRV
shlp2
M 1 2 3 4 WT KO WT KO
14 kb
9 kb
0.8 kb
0.45kb
Chr 5 - 7
HindIII
ppm1
M 1 2 3 4 WT-GFP KO WT-GFP KO
0.8 kb
0.47 kb
3.5 kb
0.5 kb
Chr 9, 10
Chr 5 - 7
Chr 1 - 4
HindIII
ppm2
M 1 2 3 4 WT-GFP KO WT-GFP KO
1.2 kb
0.7 kb
6.4 kb
0.6 kb
Chr 9 -12
Chr 5 - 7
Chr 1 - 4
EcoRV
ppm3
M 1 2 3 4 WT-GFP KO WT-GFP KO
0.8 kb
0.4 kb
10 kb
4.5 kb
Chr 13, 14
Chr 5 - 7
Chr 1 - 4
EcoRV
ppm4
M 1 2 3 4 WT-GFP KO WT-GFP KO
0.83 kb
0.6 kb
4.3 kb
2.7 kb
Chr 13 -14
Chr 5 - 7
Chr 1 - 4
EcoRI
ppm5
M 1 2 3 4 WT-GFP KO WT-GFP KO
1.4 kb
0.3 kb
7.1 kb
0.9 kb
Chr 9, 10
Chr 5 - 7
Chr 1 - 4
EcoRV
ppm7
M 1 2 3 4 WT-GFP KO
0.8 kb
0.6 kb
8 kb
3 kb
HindIII
ppm8
M 1 2 3 4 WT-GFP KO
0.97 kb
0.72 kb
4.3 kb
2.5 kb
HindIII
ptp1
M 1 2 3 4 WT-GFP KO WT-GFP KO
1 kb
0.63 kb
7.0 kb
2.5 kb
Chr 13, 14
Chr 5 - 7
Chr 1 - 4
HindIII
ptp2
M 1 2 3 4 WT-GFP KO WT-GFP KO
1.2 kb
0.4 kb
3.3 kb
1.8 kb
Chr 9, 10
Chr 5 - 7
HindIII
ptpla
M 1 2 3 4 WT-GFP KO
1 kb
0.56 kb
4.7 kb
1.6 kb
EcoRV
nif1
M 1 2 3 4 WT-GFP KO
0.93 kb
0.5 kb
6.7 kb
2.4 kb
EcoRV
Legend for PCR genotyping
1 - Disrupted locus on wild-type gDNA
2 - Disrupted locus on mutant DNA
3 - Wild-type locus on wild-type DNA
4 - Wild-type locus on mutant DNA

Figure S3

Figure S3, related to Figure 2: Generation of PP mutants and genotypic analyses

(A) Schematic representation of the endogenous protein phosphatase gene locus, the knockout construct and the recombined protein phosphatase gene locus following double cross-over recombination. The knockout construct contains a *Toxoplasma gondii* dihydrofolate reductase/thymidylate synthase (*tgdhfr/ts*) cassette with a *Pbdhfr* 3'UTR for selection of transgenic parasites with pyrimethamine. (B) Diagnostic PCR, Southern blot and pulse-field gel electrophoresis for each of the phosphatase knock out lines generated. (left) Integration PCR (lanes 1 and 2) showing the presence of a band at the indicated size using integration specific primers on gDNA of the deletion mutant (lane 2) indicating correct integration of the knockout construct, and knock out PCR (lanes 3 and 4) showing the absence of the wild-type specific band amplified by knock out specific primers demonstrating the loss of the wild-type locus in the deletion mutant (lane 4). WT gDNA was used as control. (middle) Southern blot analysis of GFP parasites gDNA (WT-GFP) and phosphatase mutant (KO) gDNA showing the bands recognised by the probe for the endogenous locus and for the recombined locus. The enzyme used for the digestion is indicated at the bottom of the blot. (right) Pulse-field gel electrophoresis (PFGE) analysis of WT-GFP (WT-GFP) and phosphatase mutant (KO) gDNA. Chromosomes on which the probe hybridises to the endogenous *dhfr* locus and the disrupted locus are indicated. Only one representative clone for each of the phosphatase deletion mutants (out of at least 2 independent clones obtained for each phosphatase deletion mutant) is presented in the phenotypic analyses.

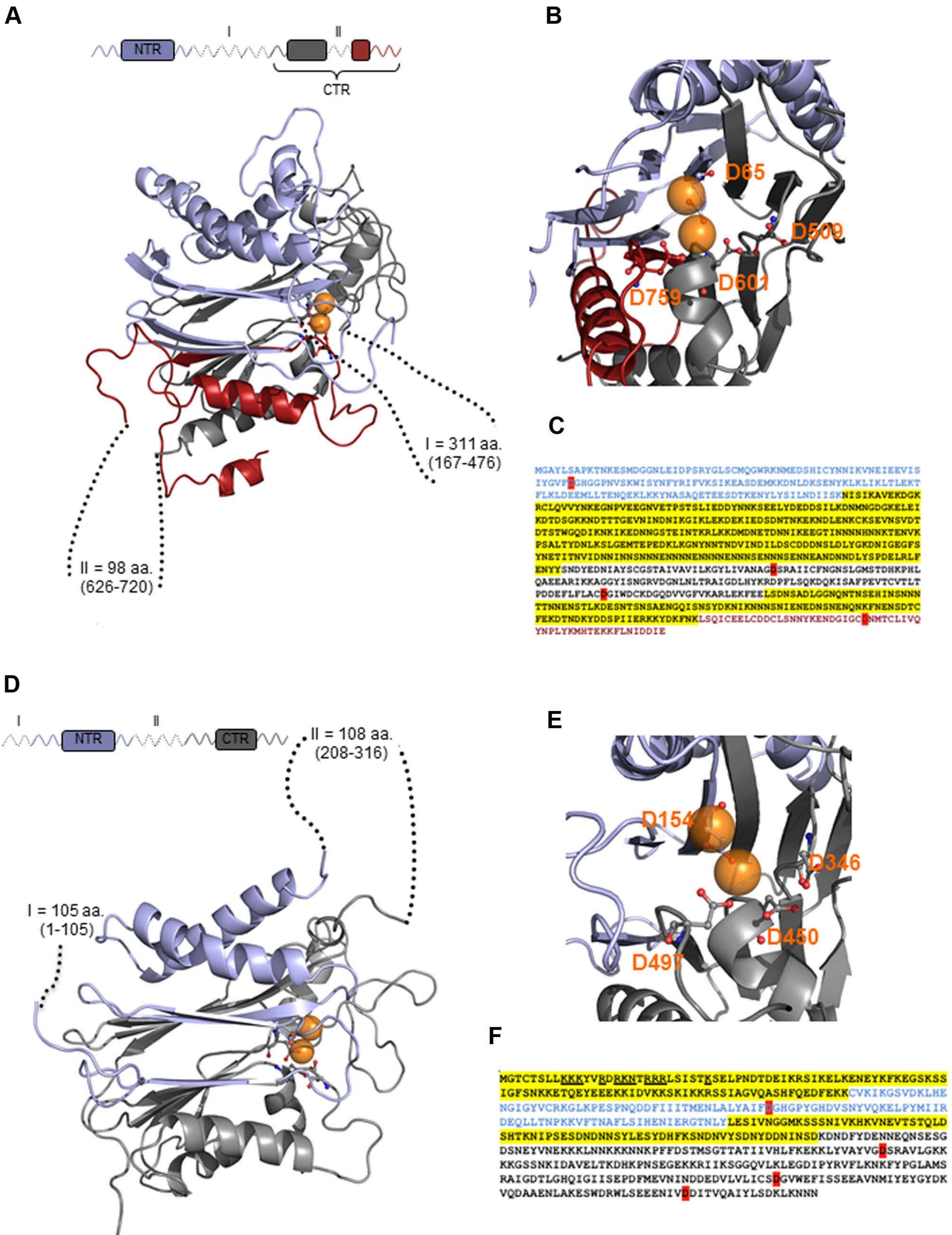
A
NTR
I
II
CTR
I = 311 aa.
(167-476)
II = 98 aa.
(626-720)
B
D65
D509
D601
D759
C
MGAYLSAPKTNKESMDGGNLEIDPSRYGLSCMQGWRKNMEDSHICYNNIKVNEIEEVIS
IYGVFDGHGGPNVSKWISYNFYRIFVKSIKEASDEMKKDNLDKSENYKLKLIKLTLEKT
FLKLDEEMLLTENQEKLKKYNASAQETEESDTKENYLYSILNDIISKNISIKAVEKDGK
RCLQVVYNKEGNPVEEGNVETPSTSLIEDDYNNKSEELYDEDDSILKDNMNGDGKELEI
KDTDSGKKNDTTTGEVNINDNIKGIKLEKDEKIEDSDNTNKEKNDLENKCKSEVNSVDT
DTSTWGQDIKNKIKEDNNGSTEINTKRLKKDMDNETDNNIKEEGTINNNNHNNKTENVK
PSALTYDNLKSLGEMTEPEDKLKGNYNNTNDVINDILDSCDDDNSLDLYGKDNIGEGFS
YNETITNVIDNNINNSNNNENNNNENNNNENNNSENNNSENNEANDNNDLYSPDELRLF
ENYYSNDYEDNIAYSCGSTAIVAVILKGYLIVANAGDSRAIICFNGNSLGMSTDHKPHL
QAEEARIKKAGGYISNGRVDGNLNLTRAIGDLHYKRDPFLSQKDQKISAFPEVTCVTLT
PDDEFLFLACDGIWDCKDGQDVVGFVKARLEKFEELSDNSADLGGNQNTNSEHINSNNN
TTNNENSTLKDESNTSNSAENGQISNSYDKNIKNNNSNIENEDNSNENQNKFNENSDTC
FEKDTNDKYDDSPIIERKKYDKFNKLSQICEELCDDCLSNNYKENDGIGCDNMTCLIVQ
YNPLYKMHTEKKFLNIDDIE
D
I
NTR
II
CTR
II = 108 aa.
(208-316)
I = 105 aa.
(1-105)
E
D154
D346
D450
D497
F
MGTCTSLLKKKYVRDRKNTRRRLSISTKSELPNDTDEIKRSIKELKENEYKFKEGSKSS
IGFSNKKETQEYEEEKKIDVKKSKIKKRSSIAGVQASHFQEDFEKKCVKIKGSVDKLHE
NGIGYVCRKGLKPESPNQDDFIIITMENLALYAIFDGHGPYGHDVSNYVQKELPYMIIR
DEQLLTNPKKVFTNAFLSIHENIERGTNLYLESIVNGGMKSSSNIVKHKVNEVTSTQLD
SHTKNIPSESDNDNNSYLESYDHFKSNDNVYSDNYDDNINSDKDNDFYDENNEQNSESG
DSNEYVNEKKKLNNKKKNNKPFFDSTMSGTTATIIVHLFKEKKLYVAYVGDSRAVLGKK
KKGSSNKIDAVELTKDHKPNSEGEKKRIIKSGGQVLKLEGDIPYRVFLKNKFYPGLAMS
RAIGDTLGHQIGIISEPDFMEVNINDDEDVLVLICSDGVWEFISSEEAVNMIYEYGYDK
VQDAAENLAKESWDRWLSEEENIVDDITVQAIYLSDKLKNNN

Figure S4

Figure S4, related to Figure 3: Structural analysis of the PPMs

Computational homology models for (A-C) PPM2 and (D-F) PPM5. (A) PPM2 is shown in a schematic representation (top) colour-matched to the molecular model (below). The extensive loop regions are indicated by dotted lines, with the loop length shown. Metal ions are shown as orange spheres. (B) Zoom into the active site, showing the aspartic acid residues that coordinate the metal ions. (C) The active site is composed of residues far apart in sequence, belonging to structural modules intercepted by long unstructured loops. The N-rich loops of PPM2 are highlighted in yellow background, the N-terminal phosphatase domain (NTR) in blue text and the C-terminal domain (CTR) is shown in black and red text. Loop I separates the NTR from the CTR and loop II bisects the CTR. The metal coordinating residues are highlighted in red background. (D-F) show PPM5 using the same representations.

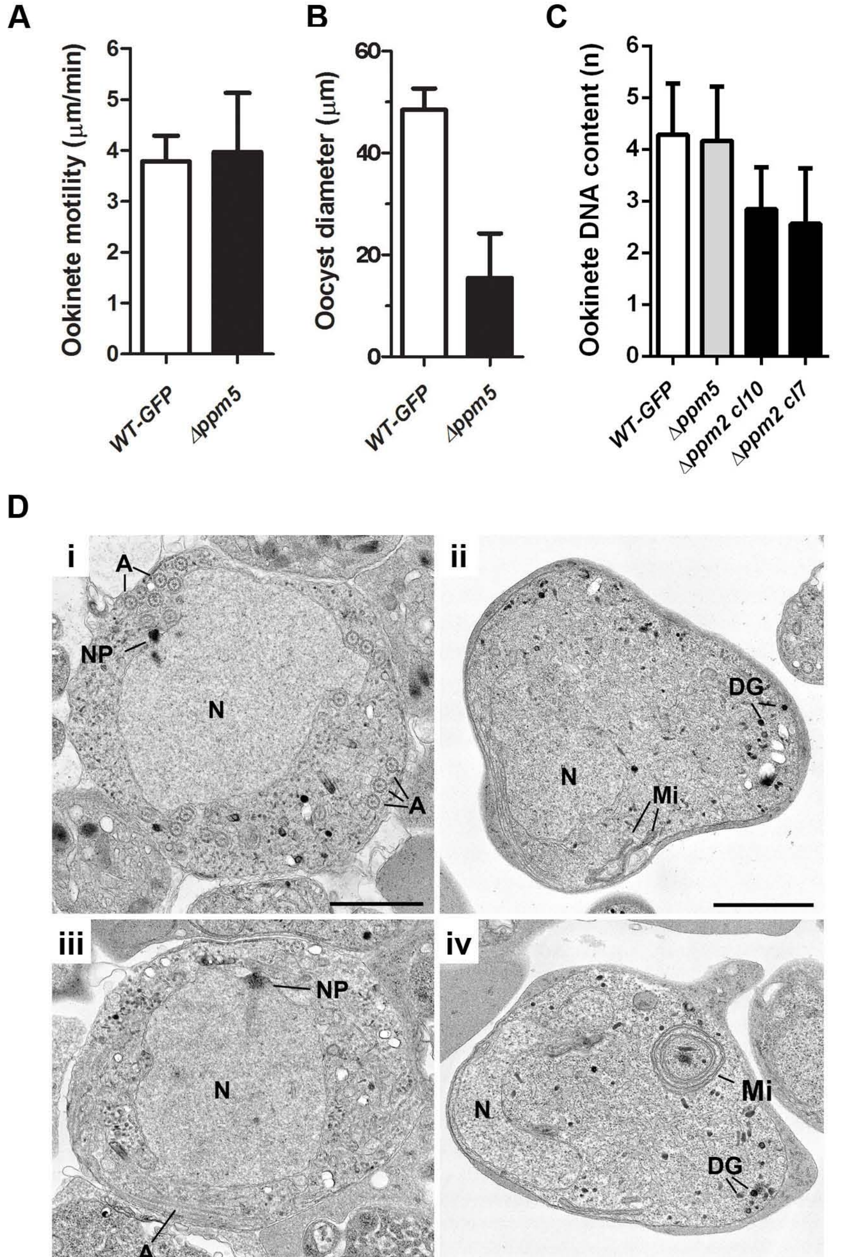
A
Ookinete motility (μm/min)
6
5
4
3
2
1
0
WT-GFP
Δppm5
B
Oocyst diameter (μm)
60
40
20
0
WT-GFP
Δppm5
C
Ookinete DNA content (n)
6
5
4
3
2
1
0
WT-GFP
Δppm5
Δppm2 cl10
Δppm2 cl7
D
i
A
NP
N
A
ii
DG
N
Mi
iii
NP
N
A
iv
N
Mi
DG

Figure S5

Figure S5, related to Figure 4: Phenotypic analysis of Δ*ppm5* and ultrastructure of Δ*ppm2* activated gametocytes

(A) Motility of Δ*ppm5* ookinetes. Velocity (µm/min) of individual WT-GFP or Δ*ppm5* ookinetes from 24 hr cultures was measured over 10 min (Error bar ±SD; n = 10). (B) Size of Δ*ppm5* oocysts. Diameter of WT-GFP or Δ*ppm5* oocysts day 14 post-infection was measured using AxioVision software (error bar ±SD; n = 45). (C) Fluorometric DNA content (n) analysis of WT-GFP and Δ*ppm5* ookinetes, and two independent clones of Δ*ppm2* (cl7 and cl10) after Hoechst nuclear staining. Nuclear fluorescence intensity of WT-GFP or mutant parasites from 24 hr cultures was measured using ImageJ software. Values are expressed relative to the average fluorescence intensity of haploid ring-stage parasites from the same slide and corrected for background fluorescence (Error bar ±SD; n = 15 for WT-GFP and Δ*ppm5* ookinetes, and n = 38 for each clone of Δ*ppm2*) (D) Electron micrographs of the activated gametocytes of wild type (i and ii) and Δ*ppm2* (iii and iv) parasites. Bar = 1 µm. (i) Section through a developing wild type microgametocyte showing the central nucleus (N) with a nuclear pole (NP) and a number of axonemes (A) within the cytoplasm. (ii) Section through a wild-type macrogametocyte showing the more peripherally located nucleus (N), with flattened mitochondria (Mi) and a few dense bodies (DG) in the cytoplasm. (iii) Section through a developing Δ*ppm2* microgametocyte showing the central nucleus (N) with a nuclear pole (NP) plus a number of axonemes (A) forming within the cytoplasm as seen in the wild type. (iv) Section through a Δ*ppm2* macrogametocyte showing the more peripherally located nucleus (N), mitochondrion (Mi) and dense bodies (DG) as seen in wild-type.

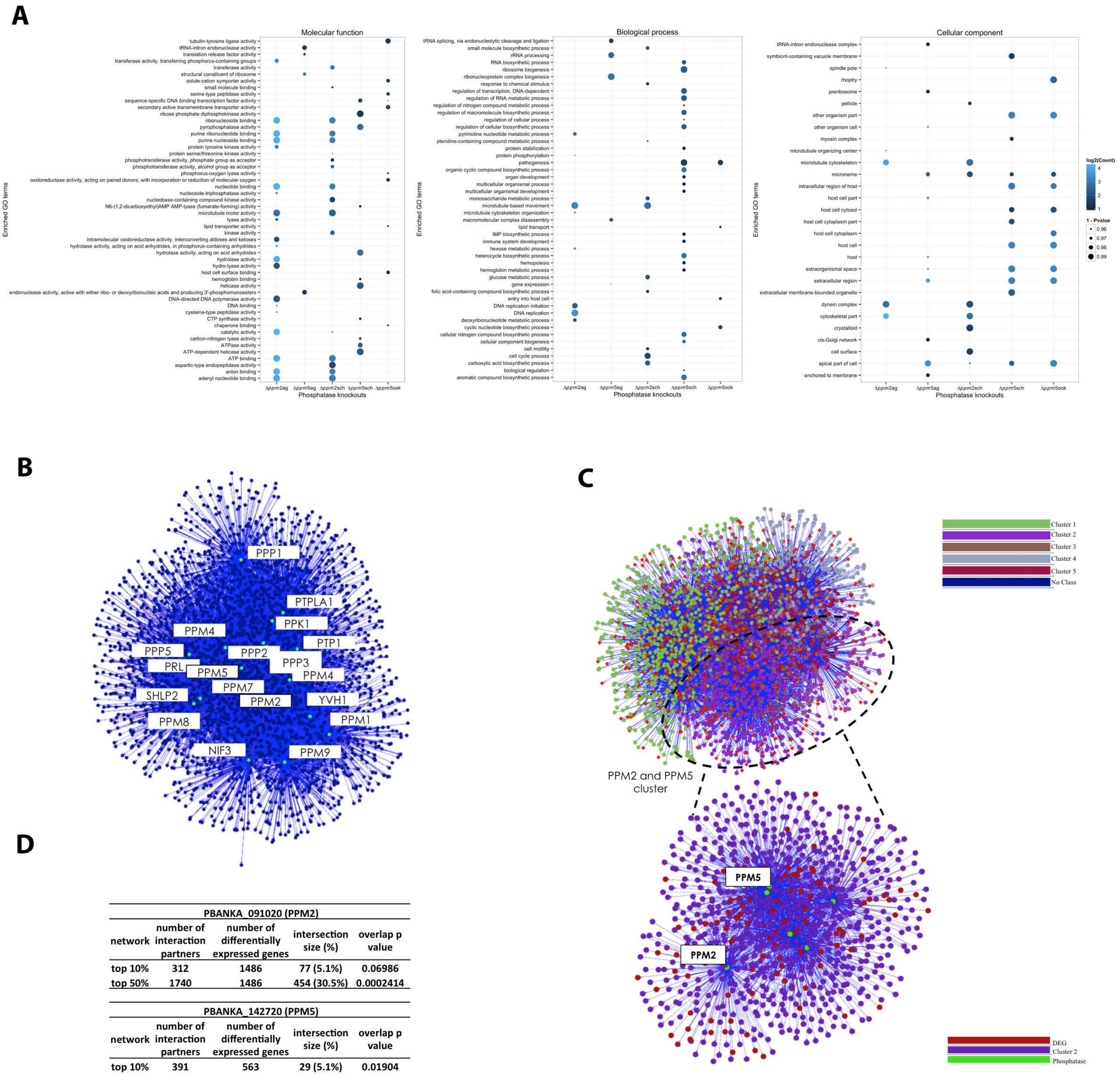

D

PBANKA_091020 (PPM2)				
network	number of interaction partners	number of differentially expressed genes	intersection size (%)	overlap p value
top 10%	312	1486	77 (5.1%)	0.06986
top 50%	1740	1486	454 (30.5%)	0.0002414

PBANKA_142720 (PPM5)				
network	number of interaction partners	number of differentially expressed genes	intersection size (%)	overlap p value
top 10%	391	563	29 (5.1%)	0.01904
top 50%	1734	563	160 (28.4%)	0.00225

Figure S6

Figure S6, related to Figure 5: Gene ontology analysis and phosphatase interaction subnetworks

(A) Gene ontology (GO) enrichment analysis of significantly differentially expressed genes in Δ*ppm2* and Δ*ppm5* in schizonts, activated gametocytes and ookinetes (only in Δ*ppm5*). GO terms obtained from the GeneDB database. Size of the bubble indicates the level of significance (1-*p* value) of the enriched GO term and colour density indicates the number of differentially expressed genes ($\log_2$ of gene count) associated with that GO term. (B) Interaction subnetwork of *P. berghei* phosphatome derived from growth perturbation transcriptome of *P. falciparum.* (C) top: 5 MCL (Markov-Cluster Algorithm http://micans.org/mcl/) clusters of the phosphatases subnetwork (refer to methods for details) bottom: zoomed view of cluster 2 where PPM2 and PPM5 co-cluster. Differentially expressed genes (coloured in red) in the Δ*ppm2 and* Δ*ppm5* PP mutants were significantly overlapping with PPM2 and PPM5 interaction partners with *p-values* 0.00024 and 0.0022, respectively. (D) Number of interaction partners for PPM2 and PPM5 in the top 10% and top 50% phosphatases subnetworks, and the significance of overlap with differentially expressed genes.

SUPPLEMENTAL TABLE LEGENDS

Table S1, related to Figure 2: Proposed protein phosphatase gene names, C-terminal GFP fusion of endogenous PPs and summary of deletion attempts

Total number of C-terminal GFP fusion transfection and gene knock-out attempts and how many led to successful tagging or successful deletion of the target as confirmed by genotypic analysis. We were unable to successfully tag PPM9. *P* value gives an estimate for the likelihood of error for "possibly essential" genes; it is calculated from the average technical failure rate (0.547 per attempt for redundant genes in this study) to the power of *n*, where *n* is the total number of deletion attempts for the gene.

Table S2, related to Figure 2: Phenotypic analysis of deletion mutants

Raw data from phenotyping analysis of 14 mutants at different development stages. All data are given as a percentage of wild-type controls studied in parallel. SD = standard deviation. *n* = number of replicate experiments.

Table S3, related to Figure 5: Significantly altered gene expression in Δ*ppm2* and Δ*ppm5* mutants compared to wild-type and putative interactions in phosphatase networks

Raw differential expression, $\log_2$ fold change values and putative interactions in phosphatise networks of significantly altered genes in Δ*ppm2* and Δ*ppm5* mutants. Gene names were obtained from GeneDB.

Table S4, related to Figure 5: Heatmap clusters and $\log_2$ ratios of gene expression

Differential expression ($\log_2$ fold change; Table S3) values used to produce the heatmaps in Figure 5C of protein phosphatases, protein kinases, RNA helicases, AP2 transcription factors, host invasion- and microneme- related proteins, microtubules/axonemes related dyneins and kinesins and enzymes involved in glycolysis (BIR proteins; not included in

Figure 5C), in Δ*ppm2* and Δ*ppm5* at schizont, activated gametocyte and ookinete life stages. Cells highlighted in green were upregulated and red were downregulated. Sch = schizonts; AG = activated gametocytes; Ook = ookinetes.

Table S5, related to Figure 2: Primers used for generation of C-terminal GFP fusion, gene deletion constructs and genotype analysis

Generic sequences for *Kpn*I and *Apa*I restriction sites; used for GFP fusion cloning purposes. ol492 sequence is given in (Guttery et al., 2012). Generic sequences for *Apa*I/*HindIII* and *EcoR*I and *Xba*I restriction sites; used for gene deletion cloning purposes. ol248 and ol539 sequences are given in (Tewari et al., 2010).

Table S6, related to Figure 3: Primers used for qRT-PCR

Sequences shown are written 5' – 3'.

SUPPLEMENTAL EXPERIMENTAL PROCEDURES

Ethics statement

All animal work has passed an ethical review process and was approved by the United Kingdom Home Office. Work was carried out in accordance with the United Kingdom 'Animals (Scientific Procedures) Act 1986' and in compliance with 'European Directive 86/609/EEC' for the protection of animals used for experimental purposes. The project licence number is 40/3344.

Animals

Six to eight week old female Tuck-Ordinary (TO) outbred mice (Harlan) were used for all experiments.

Bioinformatic analysis

PPs from a variety of families were identified in the predicted proteomes of *P. berghei*, *P. falciparum* and 44 other diverse eukaryotes for which complete genome sequence is available (see (Wickstead et al., 2010) for full list). HMMER3 (Eddy, 2009) was used to search these proteomes for matches to the PFam profiles: DSPc (PF00782.15), LMWPc (PF01451.16), Metallophos (PF00149.23), NIF (PF03031.13), PP2C (PF00481.16), PTPLA (PF04387.9) and Y-phosphatase domains (PF03162.8, PF13350.1 and PF00102.22), all with a threshold of e-value ≤ 10^{-5}. For classification, full-length proteins were clustered using a neighbour-joining approach based on all-versus-all BLASTp scores (Wickstead and Gull, 2007) and large-scale maximum-likelihood phylogenies were built from trimmed alignments of phosphatase domains (MAFFT6.24 (Katoh et al., 2002)) with statistical support for nodes from the approximate Likelihood Ratio Test (Anisimova et al., 2010) as implemented by PhyML3.0 (Guindon and Gascuel, 2003). Protein domain architectures were predicted from the models in Pfam26 with e-value ≤ 0.001. Possible myristoylation was predicted using *N*-Myristoyltransferase (NMT) Myristoylator (Bologna et al., 2004).

Generation and genotyping of transgenic parasites

For C-terminal tagging of each PP with GFP by single homologous recombination, a targeting vector was generated using the p277 cassette containing the human *dhfr* sequence (Guttery et al., 2012) (Figure S2). For gene deletion by double homologous recombination, a targeting vector was generated using the pBS-DHFR cassette (Tewari et al., 2010) (Figure S3). Both vectors confer resistance to pyrimethamine. Linear targeted sequences were released by restriction digest and transfected via electroporation (Janse et al., 2006) into either the *P. berghei ANKA* 2.34 strain or the *P. berghei ANKA* 507 cl1 line (Janse et al., 2006). For initial genotyping of parasite pools PCR and Western blotting were used for GFP-tagged lines and PCR alone for deletion mutants. After dilution cloning of mutants, deletion of the targeted region of the genome was confirmed by PCR, Southern

blotting and pulse-field electrophoresis (PFGE). See Table S5 for all oligonucleotides used for cloning in this study.

Phenotypic screening of mutants and GFP localisation

Phenotypic screening of mutants and localisation of GFP-tagged lines was performed as previously described (Guttery et al., 2012; Tewari et al., 2010). Briefly, asexual proliferation and gametocytogenesis and gametocyte sex ratios were analysed using Giemsa-stained blood smears. Exflagellation was examined on day 4–5 post infection. 10 µl of gametocyte-infected blood were obtained from the tail with a heparinised pipette tip and mixed immediately with 40 µl of ookinete culture medium (RPMI 1640 containing 25 mM HEPES, 20% foetal bovine serum, 10 mM sodium bicarbonate, and 50 mM xanthurenic acid at pH 7.6). The mixture was placed under a Vaseline-coated cover slip and 15 min later exflagellation centres were counted by phase contrast microscopy in 12–15 fields. Ookinete formation was assessed the next day; 10 µl of infected tail blood were obtained as above, mixed immediately with 40 µl ookinete culture medium, and incubated for 2 hr at 20 °C to allow completion of gametogenesis and fertilisation. Each culture was then diluted with 0.45 ml of ookinete medium and incubated at 20 °C for a further 21–24 hr to allow ookinete differentiation. Cultures were pelleted for 2 min at 5000 rpm and then incubated with 10 µl of ookinete medium containing Hoechst 33342 DNA dye and Cy3-conjugated mouse monoclonal antibody 13.1 (Tewari et al., 2005) recognising the P28 protein on the surface of ookinetes and any undifferentiated macrogametes or zygotes. P28-positive cells were counted with a Zeiss AxioImager M2 microscope (Carl Zeiss, Inc) fitted with an AxioCam ICc1 digital camera. Ookinete conversion was expressed as the percentage of P28 positive parasites that had differentiated into ookinetes. To measure nuclear DNA content of ookinetes by direct immunofluorescence, images of parasites stained as above were analysed using ImageJ software (National Institutes of Health) as previously described (Tewari et al., 2005). Motility assays were performed as described previously (Patzewitz et

al., 2013). Briefly, ookinete cultures were mixed with an equal volume of Matrigel on ice, mounted onto a slide, covered and sealed with nail varnish and left to set at room temperature for at least 30 min. Time lapse movies of ookinete movement (1 frame every 5 s for 10 min) were taken using a Zeiss AxioImager M2 microscope as described above, and their speed was determined (μm min^{-1}).

For mosquito transmission experiments, triplicate sets of 20–50 *Anopheles stephensi* SD500 mosquitoes were allowed to feed for 20 min on anaesthetised infected mice whose asexual parasitaemia had reached 5–7% and were carrying comparable numbers of gametocytes as determined on Giemsa stained blood films. On day 14 post-feeding approximately 20 mosquitoes were dissected and oocysts on their mid-guts counted. Oocyst formation was examined by Hoechst 33342 staining for 10–15 min and guts were washed and mounted under Vaseline-rimmed cover slips. Images were recorded using a Zeiss AxioImager M2 microscope fitted with an AxioCam ICc1 digital camera. Oocyst diameter was measured using AxioVision software. On day 21 post-feeding another 20 mosquitoes were dissected and their guts and salivary glands homogenised separately in a loosely fitting homogeniser to release sporozoites, which were then quantified using a haemocytometer. Due to day-to-day variations in transmission levels, all data were normalised to a matching number of wild type controls analysed on the same day.

Genetic complementation crosses were carried out between different mutant parasite lines as previously described (Guttery et al., 2012; Patzewitz et al., 2013). Briefly, for complementation of ookinete conversion, mature gametocyte-containing blood from mice infected with different parasite lines was mixed and re-suspended in ookinete medium, and ookinete conversion was determined as described above. For complementation of oocyst production, mice were infected with combinations of different parasite strains and 3-6 days old female *A. stephensi* mosquitoes were infected by directly feeding on these mice.

Mosquitoes were dissected 12-14 days post infection and the presence of oocysts was determined as described above.

Purification of schizonts, gametocytes and ookinetes

Blood stage parasites taken from infected mice (day 4 post infection) were placed in culture (40 ml RPMI 1640, 8 ml foetal bovine serum, 0.5 ml penicillin and streptomycin; per 0.5 ml blood) for 24 h at 37 °C (with rotation at 100 rpm). The following day the culture was fractionated on a 60% v/v NycoDenz gradient (NycoDenz stock solution: 27.6% w/v NycoDenz in 5 mM Tris-HCl, pH 7.20, 3 mM KCl, 0.3 mM EDTA; CLB: PBS, 20 mM HEPES, 20 mM Glucose, 4 mM sodium bicarbonate, 1 mM EGTA, 0.1% w/v bovine serum albumin, pH 7.25). The purification of gametocytes was based on a modified protocol from (Beetsma et al., 1998). On day four post-infection mice were treated with sulfadiazine (Sigma, 20 mg/l in drinking water) for two days to eliminate asexual blood stage parasites. On day six post-infection the parasites were harvested, kept on ice to avoid premature activation and separated from uninfected erythrocytes on a 48% (v/v) NycoDenz in coelenterazine loading buffer (CLB) gradient. Gametocytes were harvested from the interface and washed twice in RPMI 1640 before activation of gamete formation in ookinete medium for 30 min at 20 ^{o}C. For ookinete preparation, parasites from day 5 post infection mice were placed in 1 ml ookinete medium for 24 hr at 20 °C for ookinete production. The parasites were then lysed in red blood cell (RBC) buffer for 30 min and purified on a 63% NycoDenz gradient (v/v in CLB).

Isolation of PPM2-GFP and PPM5-GFP proteins, and subcellular fractionation

Immunoprecipitation and subcellular fractionation of GFP-tagged proteins were performed as described previously (Guttery et al., 2012). Briefly, the cell pellets obtained from blood of mice infected with parasites expressing GFP, PPM2-GFP and PPM5-GFP were resuspended in hypotonic lysis buffer (10 mM Tris-HCl pH 8.4, 5 mM EDTA) containing

protease inhibitors (Roche), freeze/thawed twice, incubated for 1 hr at 4°C and centrifuged at 100,000 g for 30 min. The supernatants obtained were collected as the soluble protein fraction (S). The corresponding pellets were then washed, resuspended in carbonate solution (0.1M Na_2CO_3, pH 11.0) containing protease inhibitors (Roche), incubated for 30 min at 4°C and centrifuged again at 100,000 g for 30 min. The resulting supernatants were saved as the peripheral membrane fraction (PM) and the pellets were washed and solubilised in 4% SDS and 0.5% Triton X-100 in PBS, forming the integral membrane fraction (IM). Equal amounts of these three fractions were then analysed by western blot using anti-GFP antibody.

PPM2 and PPM5 *in vivo* phosphorylation

As described previously (Guttery et al., 2012), schizonts and activated gametocytes (purified as described above) were washed in phosphate-free Krebs buffer and metabolically labelled with 3–5 MBq [^{32}P]-orthophosphate (Perkin Elmer) in the same buffer for 30 min at 20 °C or 37 °C for activated gametocytes and schizonts, respectively. Following two washes in phosphate-free Krebs buffer, the labelled parasites were lysed for 30 min at 4 °C in lysis buffer (10 mM Tris-HCl pH 7.5, 150 mM NaCl, 0.5 mM EDTA, 0.5% NP-40) supplemented with protease and phosphatase inhibitors (both Roche). The resulting lysate was centrifuged at 20,000 g for 10 min and the supernatant collected. GFP tagged proteins were then isolated using GFP-TRAP beads (ChromoTek) according to the manufacturer's instructions and the immunoprecipitated proteins were subsequently resuspended in Laemmli sample buffer for separation by SDS-PAGE. [^{32}P]-labelled proteins were visualised using a phosphorimager (Molecular Dynamics) and GFP-tagged proteins analysed by Western blot using anti-GFP antibody.

Phosphatase activity assay

Protein phosphatase activity of the immunoprecipitated PPM2-GFP and PPM5-GFP was assessed using the SensoLyte MFP Protein Phosphatase Assay Kit (AnaSpec) according to manufacturer's instructions. Briefly, blood aliquots from infected mice (with GFP, PPM2-GFP and PPM5-GFP parasites) were processed as described previously (Guttery et al., 2012). The parasite pellets were lysed for 30 min at 4 °C in lysis buffer (ChromoTek) supplemented with protease inhibitors (Roche), and the resulting lysates were then immunoprecipitated using GFP-TRAP beads (ChromoTek) according to manufacturer's instructions. The GFP-TRAP beads were resuspended and diluted in phosphatase assay buffer (100 mM Tris-HCl pH 7.5, 4 mM DTT, 0.2 mM EDTA, 0.5 mM $MnCl_2$, 0.4 mg/ml BSA), incubated for 30 min at 37 °C in the presence or absence of MFP fluorogenic phosphatase substrate, and centrifuged for 2 min at 2700 g. Supernatants were transferred to a 96-well microplate and the fluorescence generated by the dephosphorylation of MFP was measured using a microplate fluorimeter.

Metabolic labelling, purification and detection of *N*-myristoylated proteins

To metabolically label *N*-myristoylated proteins, a recently described procedure was followed (Poulin et al., 2013; Wright et al., 2014). Briefly the blood of one infected mouse was placed in schizont medium containing 50 µM YnMyr and left overnight at 37 °C before purification as described above. Parasite proteins were extracted using 0.1% SDS, 1% Triton X-100 in 10 mM Na_2PO_4, pH 8.2 with protease inhibitors (EDTA-free, Roche). Extracts were pelleted and the concentration of protein in the supernatant determined by DC protein assay (Bio-Rad). Protein lysates were labelled and precipitated as described previously (Heal et al., 2012). Protein was redissolved at 10 mg/ml in 2% SDS, 10 mM EDTA in PBS, and then diluted to 1 mg/ml with PBS. Aliquots were removed for pre-enrichment analysis. Proteins were incubated with Dynabeads MyOne Streptavidin C1 for 2 hr at RT. Following removal of the supernatant, beads were washed with 1% SDS in PBS, and then boiled for 10 min in sample loading buffer to elute bound proteins. For immunoblotting, proteins were transferred

to PVDF membranes, membranes were blocked (5% dried skimmed milk in TBS 0.1% Tween-20), then probed with anti-GFP (rabbit polyclonal, 1:2,000, Invitrogen), followed by anti-rabbit HRP secondary antibody (goat anti-rabbit, 1:10,000, Invitrogen) in blocking solution, and developed with Luminata Crescendo Western HRP substrate (Millipore) according to the manufacturer's instructions on a Fujifilm LAS 3000 imager.

Electron microscopy

Gametocyte and ookinete samples cultured in ookinete medium as described above were fixed in 4% glutaraldehyde in 0.1 M phosphate buffer and processed for routine electron microscopy as previously described (Guttery et al., 2012). Samples were post fixed in osmium tetroxide, treated en bloc with uranyl acetate, dehydrated and embedded in Spurr's epoxy resin. Thin sections were stained with uranyl acetate and lead citrate prior to examination in a JEOL1200EX electron microscope (Jeol UK Ltd).

Quantitative RT-PCR

Total RNA was isolated from purified parasites using an RNeasy purification kit (Qiagen). For qRT-PCR, cDNA was synthesised using an RNA-to-cDNA kit (Applied Biosystems) allowing quantification from 250 ng of total RNA. qRT-PCR reactions consisted of 2 µl cDNA, 5 µl SYBR green fast master mix (Applied Biosystems), 0.5 µl (500 nM) each of the forward and reverse primers, and 2 µl DEPC-treated water. Where possible, one of the primer pairs was placed over an intron, primers had melting temperatures of 60-62 ^{o}C and together amplified a region 70-200 bp long. Analysis was conducted using an Applied Biosystems 7500 fast machine with the following cycling conditions: 95 °C for 20 sec followed by 40 cycles of 95 °C for 3 sec; 60 °C for 30 sec. Wild-type expression was determined using the Pfaffl method (Pfaffl, 2001). Relative quantification in the mutant line was normalised against wild-type expression using the $\Delta\Delta C_t$ method. Both methods used *hsp70* (PBANKA_081890) and *seryl-tRNA synthetase* (PBANKA_061540) as reference genes. Three biological

replicates were used for each stage (each with two technical replicates). See Table S6 for a full list of the primers used for qRT-PCR.

Transcriptome sequencing and RNA-Seq analysis

Parasites were collected from Δ*ppm2*, Δ*ppm5* or GFP-expressing lines at three developmental stages (schizonts, activated gametocytes and ookinetes). Total RNA was isolated from purified parasites using an RNeasy purification kit (Qiagen) as described for qRT-PCR but was also passed through a plasmodipur column to remove host DNA contamination prior to RNA isolation. RNA was vacuum concentrated (SpeedVac) and transported using RNA stable tubes (Biomatrica). Validation used different biological replicates to the RNA-seq samples to validate the data set more robustly (Allison et al., 2006). Strand-specific mRNA sequencing was performed from total RNA using TruSeq Stranded mRNA Sample Prep Kit LT (Illumina) according to manufacturer's instructions. Briefly, polyA+ mRNA was purified from total RNA using oligo-dT dynabead selection. First strand cDNA was synthesised using randomly primed oligos followed by second strand synthesis where dUTPs were incorporated to achieve strand-specificity. The cDNA was adapter-ligated and the libraries amplified by PCR. Libraries were sequenced in Illumina Hiseq with paired-end 100bp read chemistry.

RNA-seq read alignment and differential gene expression analysis were performed using the Tophat-Cufflinks pipeline (Trapnell et al., 2012). Strand-specific RNA-seq paired-end reads were mapped onto the *P. berghei* ANKA genome (PlasmoDB-9.2) using TopHat version 2.0.8 (Trapnell et al., 2009) with options '--library-type=fr-firststranded' and '--no-novel-juncs'. The aligned reads were quantified, normalised andcompared across different samples (2-4 biological replicates per sample) using Cuffdiff version 2.1 (Trapnell et al., 2013). *P. berghei* genome sequence was provided as a FASTA file to Cuffdiff to account for sequence bias in read alignment and a gff file consisting of highly variable tRNA, rRNA and

mitochondrial genes was provided to mask them from further analysis. The Cuffdiff output was visualised using R package CummeRbund (http://compbio.mit.edu/cummeRbund/index.html). The correlation between biological replicates vary from r^2 = 0.8-0.98, therefore were tightly correlated.

P. berghei phosphatome interaction network

The microarray data of P. *falciparum* global transcriptional responses to 20 growth-inhibiting compounds (Hu et al., 2010) was used to build the phosphatases interaction subnetwork (Figure S6B). DNA microarray-based profiling of growth perturbations in *P. falciparum* was previously used to generate a high-resolution transcriptional data set that reflects functional relationships between P. *falciparum* genes (Hu et al., 2010). 21 P. *falciparum* phosphatases corresponding to 21 P. *berghei* orthologoues were included in the growth perturbations dataset. We used a general method (Feizi et al., 2013), called "Network Deconvolution (ND)", which infers direct effects from an observed correlation matrix containing both direct and indirect effects. First, we applied the context likelihood of relatedness (CLR) algorithm (Faith et al., 2007) to the growth perturbations dataset to construct a relevance network. ND was then applied to the CLR-predicted network. The top 10% (15,826 linkages) and 50% (76,366 linkages) of edge predictions were used to construct two independent networks.

From each network, a phosphatase subnetwork was extracted, which we hereafter refer to as top 10% and top 50% phosphatase subnetworks. Both networks were then transformed by orthology into *P. berghei* using the "Transform by Orthology" tool in PlasmoDB http://plasmodb.org/plasmo/. The connectivity of the top 10% phosphatases subnetwork fit a power-law distribution with power (λ) value of 1.009 (R^2 = 0.78). This distribution represents a typical scale-free network, well known for protein-protein interaction networks in eukaryotic cells: a small number of highly connected nodes (hubs) are linked to a large number of less connected nodes. For the top 50% subnetwork, λ was 0.649 (R^2 = 0.523). To distinguish direct targets for PPM2 and PPM5, we tested the lists of significantly affected genes in

Δ*ppm2 and* Δ*ppm5* for overlap with the interaction partners of PPM2 and PPM5, respectively using the "GeneOverlap" package (http://shenlab-sinai.github.io/shenlab-sinai/) and Pearson's Chi-squared test. Results for the numbers of interaction partners for each of the phosphatases (PPM2 and PPM5), numbers of differentially expressed genes (q-value ≤ 0.01) and *p-value* for overlap are listed in Figure S6D. Biolayout (http://www.biolayout.org/, (Enright and Ouzounis, 2001) was used for visualisation. MCL (Markov-Cluster Algorithm http://micans.org/mcl/) was used for interaction subnetworks where PPM2 and PPM5 co-clustered.

Data deposition

RNA-Seq data has been deposited at the European Nucleotide Archive (ENA - http://www.ebi.ac.uk/ena) under accession number PRJEB5218.

Computational modelling

The sequences of all PPs were analysed by computational structural methods. All sequences were submitted to the RaptorX (raptorx.uchicago.edu/), Phyre^2 (www.sbg.bio.ic.ac.uk/phyre2/) and Swiss-Model (swissmodel.expasy.org/) servers. For PPMs with extensive loop regions, sequences with pruned loops were also submitted. Models obtained were further refined using the ModRefiner server (zhanglab.ccmb.med.umich.edu/ModRefiner/). Models were visualised and analysed using Pymol (www.pymol.org).

Gene ontology (GO) terms enrichment analysis

GO IDs were extracted from the *P. berghei* ANKA annotation gff file (available in GeneDB) and the differentially expressed gene lists were tested against a filtered universal list of 2133 *P. berghei* genes with annotated GO terms. Enriched GO terms (p value <0.05) were

identified through conditional hypergeometric testing using GOstats R package (Falcon and Gentleman, 2007) and plotted using ggplot2 R package (http://ggplot2.org/).

SUPPLEMENTAL REFERENCES

Allison, D.B., Cui, X., Page, G.P., and Sabripour, M. (2006). Microarray data analysis: from disarray to consolidation and consensus. Nature reviews Genetics *7*, 55-65.

Anisimova, M., Cannarozzi, G.M., and Liberles, D.A. (2010). Finding the balance between the mathematical and biological optima in multiple sequence alignment. Trends Evol Biol *2*, e7.

Beetsma, A.L., van de Wiel, T.J., Sauerwein, R.W., and Eling, W.M. (1998). Plasmodium berghei ANKA: purification of large numbers of infectious gametocytes. Experimental parasitology *88*, 69-72.

Bologna, G., Yvon, C., Duvaud, S., and Veuthey, A.L. (2004). N-Terminal myristoylation predictions by ensembles of neural networks. Proteomics *4*, 1626-1632.

Eddy, S.R. (2009). A new generation of homology search tools based on probabilistic inference. Genome informatics International Conference on Genome Informatics *23*, 205-211.

Enright, A.J., and Ouzounis, C.A. (2001). BioLayout--an automatic graph layout algorithm for similarity visualization. Bioinformatics *17*, 853-854.

Faith, J.J., Hayete, B., Thaden, J.T., Mogno, I., Wierzbowski, J., Cottarel, G., Kasif, S., Collins, J.J., and Gardner, T.S. (2007). Large-scale mapping and validation of Escherichia coli transcriptional regulation from a compendium of expression profiles. PLoS Biol *5*, e8.

Falcon, S., and Gentleman, R. (2007). Using GOstats to test gene lists for GO term association. Bioinformatics *23*, 257-258.

Feizi, S., Marbach, D., Medard, M., and Kellis, M. (2013). Network deconvolution as a general method to distinguish direct dependencies in networks. Nat Biotechnol *31*, 726-733.

Guindon, S., and Gascuel, O. (2003). A simple, fast, and accurate algorithm to estimate large phylogenies by maximum likelihood. Systematic biology *52*, 696-704.

Guttery, D.S., Poulin, B., Ferguson, D.J., Szoor, B., Wickstead, B., Carroll, P.L., Ramakrishnan, C., Brady, D., Patzewitz, E.M., Straschil, U.*, et al.* (2012). A unique protein phosphatase with kelch-like domains (PPKL) in Plasmodium modulates ookinete differentiation, motility and invasion. PLoS pathogens *8*, e1002948.

Heal, W.P., Wright, M.H., Thinon, E., and Tate, E.W. (2012). Multifunctional protein labeling via enzymatic N-terminal tagging and elaboration by click chemistry. Nature protocols *7*, 105-117.

Hu, G., Cabrera, A., Kono, M., Mok, S., Chaal, B.K., Haase, S., Engelberg, K., Cheemadan, S., Spielmann, T., Preiser, P.R.*, et al.* (2010). Transcriptional profiling of growth perturbations of the human malaria parasite Plasmodium falciparum. Nature biotechnology *28*, 91-98.

Janse, C.J., Franke-Fayard, B., Mair, G.R., Ramesar, J., Thiel, C., Engelmann, S., Matuschewski, K., van Gemert, G.J., Sauerwein, R.W., and Waters, A.P. (2006). High efficiency transfection of Plasmodium berghei facilitates novel selection procedures. Molecular and biochemical parasitology *145*, 60-70.

Katoh, K., Misawa, K., Kuma, K., and Miyata, T. (2002). MAFFT: a novel method for rapid multiple sequence alignment based on fast Fourier transform. Nucleic acids research *30*, 3059-3066.

Patzewitz, E.M., Guttery, D.S., Poulin, B., Ramakrishnan, C., Ferguson, D.J., Wall, R.J., Brady, D., Holder, A.A., Szoor, B., and Tewari, R. (2013). An ancient protein phosphatase, SHLP1, is critical to microneme development in Plasmodium ookinetes and parasite transmission. Cell reports *3*, 622-629.

Pfaffl, M.W. (2001). A new mathematical model for relative quantification in real-time RT-PCR. Nucleic acids research *29*, e45.

Poulin, B., Patzewitz, E.M., Brady, D., Silvie, O., Wright, M.H., Ferguson, D.J., Wall, R.J., Whipple, S., Guttery, D.S., Tate, E.W., *et al.* (2013). Unique apicomplexan IMC sub-compartment proteins are early markers for apical polarity in the malaria parasite. Biology open *2*, 1160-1170.

Tewari, R., Dorin, D., Moon, R., Doerig, C., and Billker, O. (2005). An atypical mitogen-activated protein kinase controls cytokinesis and flagellar motility during male gamete formation in a malaria parasite. Molecular microbiology *58*, 1253-1263.

Tewari, R., Straschil, U., Bateman, A., Bohme, U., Cherevach, I., Gong, P., Pain, A., and Billker, O. (2010). The systematic functional analysis of Plasmodium protein kinases identifies essential regulators of mosquito transmission. Cell host & microbe *8*, 377-387.

Trapnell, C., Hendrickson, D.G., Sauvageau, M., Goff, L., Rinn, J.L., and Pachter, L. (2013). Differential analysis of gene regulation at transcript resolution with RNA-seq. Nature biotechnology *31*, 46-53.

Trapnell, C., Pachter, L., and Salzberg, S.L. (2009). TopHat: discovering splice junctions with RNA-Seq. Bioinformatics *25*, 1105-1111.

Trapnell, C., Roberts, A., Goff, L., Pertea, G., Kim, D., Kelley, D.R., Pimentel, H., Salzberg, S.L., Rinn, J.L., and Pachter, L. (2012). Differential gene and transcript expression analysis of RNA-seq experiments with TopHat and Cufflinks. Nature protocols *7*, 562-578.

Wickstead, B., and Gull, K. (2007). Dyneins across eukaryotes: a comparative genomic analysis. Traffic *8*, 1708-1721.

Wickstead, B., Gull, K., and Richards, T.A. (2010). Patterns of kinesin evolution reveal a complex ancestral eukaryote with a multifunctional cytoskeleton. BMC evolutionary biology *10*, 110.

Wright, M.H., Clough, B., Rackham, M.D., Rangachari, K., Brannigan, J.A., Grainger, M., Moss, D.K., Bottrill, A.R., Heal, W.P., Broncel, M., *et al.* (2014). Validation of N-myristoyltransferase as an antimalarial drug target using an integrated chemical biology approach. Nat Chem *6*, 112-121.
