# Supplementary material for: Genome-wide Functional Analysis of Plasmodium Protein Phosphatases Reveals Key Regulators of Parasite Development and Differentiation
Source: Cell Host Microbe. 2014 Jul 9;16(1):128–40. doi: 10.1016/j.chom.2014.05.020 (PMC4094981; doi:10.1016/j.chom.2014.05.020)
Supplement: Document S2. Article plus Supplemental Information [file mmc8.pdf]

Cell Host & Microbe
Resource

Genome-wide Functional Analysis of *Plasmodium* Protein Phosphatases Reveals Key Regulators of Parasite Development and Differentiation

David S. Guttery,[1,7] Benoit Poulin,[1] Abhinay Ramaprasad,[2] Richard J. Wall,[1] David J.P. Ferguson,[3] Declan Brady,[1] Eva-Maria Patzewitz,[1] Sarah Whipple,[1] Ursula Straschil,[4] Megan H. Wright,[5] Alyaa M.A.H. Mohamed,[2] Anand Radhakrishnan,[2] Stefan T. Arold,[2] Edward W. Tate,[5] Anthony A. Holder,[6,*] Bill Wickstead,[1,8] Arnab Pain,[2,8] and Rita Tewari[1,*]

[1]Centre for Genetics and Genomics, School of Life Sciences, Queens Medical Centre, University of Nottingham, Nottingham NG2 7UH, UK
[2]Computational Bioscience Research Center (CBRC), Biological and Environmental Sciences and Engineering (BESE) Division, King Abdullah University of Science and Technology, Thuwal 23955-6900, Kingdom of Saudi Arabia
[3]Nuffield Department of Clinical Laboratory Science, University of Oxford, John Radcliffe Hospital, Oxford OX3 9DU, UK
[4]Division of Cell and Molecular Biology
[5]Department of Chemistry
Imperial College London, Exhibition Road, London SW7 2AZ, UK
[6]Division of Parasitology, MRC National Institute for Medical Research, Mill Hill, London NW7 1AA, UK
[7]Present address: Department of Cancer Studies and Molecular Medicine, University of Leicester, Robert Kilpatrick Building, P.O. Box 65, Leicester Royal Infirmary, Leicester LE2 7LX, UK
[8]These authors contributed equally to this work
*Correspondence: aholder@nimr.mrc.ac.uk (A.A.H.), rita.tewari@nottingham.ac.uk (R.T.)
http://dx.doi.org/10.1016/j.chom.2014.05.020
This is an open access article under the CC BY license (http://creativecommons.org/licenses/by/3.0/).

SUMMARY

Reversible protein phosphorylation regulated by kinases and phosphatases controls many cellular processes. Although essential functions for the malaria parasite kinome have been reported, the roles of most protein phosphatases (PPs) during *Plasmodium* development are unknown. We report a functional analysis of the *Plasmodium berghei* protein phosphatome, which exhibits high conservation with the *P. falciparum* phosphatome and comprises 30 predicted PPs with differential and distinct expression patterns during various stages of the life cycle. Gene disruption analysis of *P. berghei* PPs reveals that half of the genes are likely essential for asexual blood stage development, whereas six are required for sexual development/sporogony in mosquitoes. Phenotypic screening coupled with transcriptome sequencing unveiled morphological changes and altered gene expression in deletion mutants of two *N*-myristoylated PPs. These findings provide systematic functional analyses of PPs in *Plasmodium*, identify how phosphatases regulate parasite development and differentiation, and can inform the identification of drug targets for malaria.

INTRODUCTION

Malaria, caused by infection with the apicomplexan parasite *Plasmodium*, is transmitted via the female *Anopheles* mosquito and in 2012 resulted in approximately 207 million clinical infections and over 600,000 deaths (WHO, 2013). The *Plasmodium* life cycle progresses through several morphologically distinct developmental stages, including asexual proliferation in hepatocytes, followed by clinically overt intraerythrocytic multiplication in the vertebrate host. Ingestion of developmentally arrested gametocytes initiates sexual development of the parasite in the mosquito, with eventual migration to the salivary glands and transmission during feeding (Bannister and Sherman, 2009). During each stage the parasite utilizes a number of signal transduction mechanisms, including reversible protein phosphorylation catalyzed by protein kinases (PKs) and phosphatases (PPs). This mechanism of signaling is a conserved, ubiquitous regulatory process for many eukaryotic and prokaryotic cellular pathways (Cohen, 2000). However, while PKs are well recognized as important therapeutic targets (Doerig et al., 2010), PPs are only now emerging as targets for clinical intervention (Moorhead et al., 2007).

Sequence analysis of the *Plasmodium falciparum* parasite has revealed approximately 85 putative PK and 27 putative PP catalytic subunits encoded in its genome (the *Plasmodium* protein phosphatome being one of the smallest of the eukaryotic phyla) (Ward et al., 2004; Wilkes and Doerig, 2008). Recent functional analyses of the entire kinome in both the human *P. falciparum* and rodent *Plasmodium berghei* models have shown asexual stage essentiality for over half of their kinases, with a further 14 PKs having a specific function during sexual development (Solyakov et al., 2011; Tewari et al., 2010).

Although it was recently recognized as a putative target for therapeutic intervention, there is lack of systematic functional analyses of the complementary *Plasmodium* phosphatome (previously classified into four major groups: phosphoprotein phosphatases [PPPs], metallo-dependent protein phosphatases

[PPMs], protein tyrosine phosphatases [PTPs], and NLI-interacting factor-like phosphatases [NIFs], as well as a number of smaller classes) (Kutuzov and Andreeva, 2008; Moorhead et al., 2009; Wilkes and Doerig, 2008). Two nonconventional PPs, one containing an N-terminal β-propeller formed by kelch-like motifs (PPKL) and the other a *Shewanella*-like PP (SHLP1), are required during ookinete-to-oocyst transition and subsequent transmission in the in vivo rodent malaria model *P. berghei* (Guttery et al., 2012; Patzewitz et al., 2013). More recently, a second SHLP family member (SHLP2) was implicated in dephosphorylation of the host protein Band 3 during merozoite invasion of erythrocytes (Fernandez-Pol et al., 2013). However, the functional role of no other PP in any of the four major groups is known (Moorhead et al., 2009; Wilkes and Doerig, 2008).

Here, we use *P. berghei* to systematically analyze the entire *Plasmodium* protein phosphatome and, where possible, assign functions for each PP throughout the life cycle in vivo, including during mosquito transmission. Furthermore, we elucidate the expression and subcellular localization of each PP by use of endogenously tagged C-terminal GFP fusion proteins. We show that the *P. berghei* protein phosphatome is highly conserved with that of *P. falciparum*, and that expression of each PP is highly variable throughout its life cycle. In our genome-wide deletion study, we assigned functions to 14 PPs, 6 of which are essential for sexual development and differentiation during gamete-to-ookinete/oocyst transition in vivo. In-depth functional analysis of two unique *N*-myristolated PP mutants identified key roles in sex allocation, zygote differentiation, and sporogony. Further global analysis of transcript levels revealed changes in membrane structure, invasion, and cell-cycle and sporogony gene families, substantiating the phenotypic analysis. This systematic functional analysis provides a genome-wide identification of key signaling networks in both asexual blood stages and mosquito transmission. Overall, it is an important study complementary to that of the kinome toward understanding the complex signaling networks via reversible phosphorylation in *Plasmodium*.

RESULTS AND DISCUSSION

The *Plasmodium* Phosphatome Is Diverse and Largely Conserved Between Species

To define the phosphatome for human and rodent malaria, PPs encoded in the genomes of *P. berghei* and *P. falciparum* were identified by similarity to hidden Markov models of known PP catalytic domains. PFam domains were used to define protein sets with similarity to PPP, PTP, PPM, NIF-like, and PTP-like A families. As previously observed (Wilkes and Doerig, 2008), there are no predicted PPs with good similarity to the low-molecular weight phosphatase (LMWP) or CDC25 families. There are also no good matches to models of SSU72 RNA polymerase II CTD PP or Eyes Absent (EYA) PP. Other PFam domains specific to PP catalytic domains are subclasses of the above families. The 5 identified *Plasmodium* PP families were compared to 4,969 PP-like proteins from 44 diverse eukaryotes (Wickstead et al., 2010), to classify them and eliminate PP-like proteins with confirmed nonprotein PP functions. These data are summarized in Figure 1.

We found that the *P. berghei* and *P. falciparum* phosphatomes consist of 30 and 29 PPs, respectively, encompassing 28 direct orthologs across the 5 PP families described above. Our *P. falciparum* phosphatome includes the 27 previously identified PPs (Wilkes and Doerig, 2008), an additional PPP-type protein (PPP8; PF3D7_1018200), and a PTP-like A homolog (PTPLA; PF3D7_1331600) also annotated as 3-hydroxyacyl-CoA dehydratase (see Table S1, available online, for full list of accession numbers). 3D structural homology modeling supported the presence of a bona fide PP catalytic domain for all included PPs, except for PTPLA and YVH1, for which no suitable template structures are available. However, PfYVH1 has been experimentally proven to be a PP (Kumar et al., 2004). Based on available experimental template structures with substrate, phosphate, and/or metal ions bound, computational structural analysis has further supported the presence and correct position of phosphate and metal-coordinating residues in all PPPs, NIFs, and PPMs, with the exception of PPM8, which shows metal coordinating residues but lacks an obvious phosphate-binding arginine or lysine.

As found with the kinome (Tewari et al., 2010), the phosphatome is highly conserved with only three proteins without direct orthology between *P. falciparum* and *P. berghei* (Figure 1). On the basis of catalytic domain phylogeny and domain architecture, the *Plasmodium* PPP-type PPs can be further classified into subfamilies, with PPP1–PPP7 corresponding to the animal PP1–PP7 types. *Plasmodium* PPPs also include the BSU-like PP PPKL, an EF-hand-containing PP (EFPP), and the two SHLPs, none of which are present in the host (Moorhead et al., 2009).

Plasmodium PPMs and PTPs generally do not fall into well-conserved subclasses. The most broadly conserved is PPM8, which is part of a group containing human and yeast pyruvate dehydrogenase PP (Figure S1). The divergent PPM10 contains a domain type also found in the bacterial membrane-associated PP, SpoIIE, and yeast mitochondrial PTC7. This protein is encoded in the *P. falciparum* but not the *P. berghei* genome. The plasmodial PPMs include three proteins containing predicted sites for N-terminal myristoylation (PPM2, PPM5, and PPM6). PPM5 and PPM6 are similar to a group of plant PPMs of unknown function, whereas PPM2 is similar to human PP1G and yeast PTC2 and PTC3. Several of the similar PPMs in other species also contain sites for possible myristoylation, although to our knowledge none has been experimentally validated.

Expression and Subcellular Localization of the *Plasmodium* PPs Is Highly Diverse

Very little is known about the expression profiles of *Plasmodium* PPs. Therefore, we used single homologous recombination to endogenously tag each of the *P. berghei* PPs with the green fluorescent protein (GFP) at their C terminus and assessed protein expression and localization at five key stages of the parasite life cycle (Figure 2A). A total of 152 individual transfection attempts were performed, resulting in successful tagging of 29 PPs as determined by western blotting and fluorescence microscopy (Figure S2B; Table S1). Despite 18 attempts, we were unable to endogenously tag PPM9, suggesting that modification of this gene is detrimental for asexual blood stage development.

Overall, expression of each PP was highly variable, with localization falling into three major classes—localized/heterogeneous,

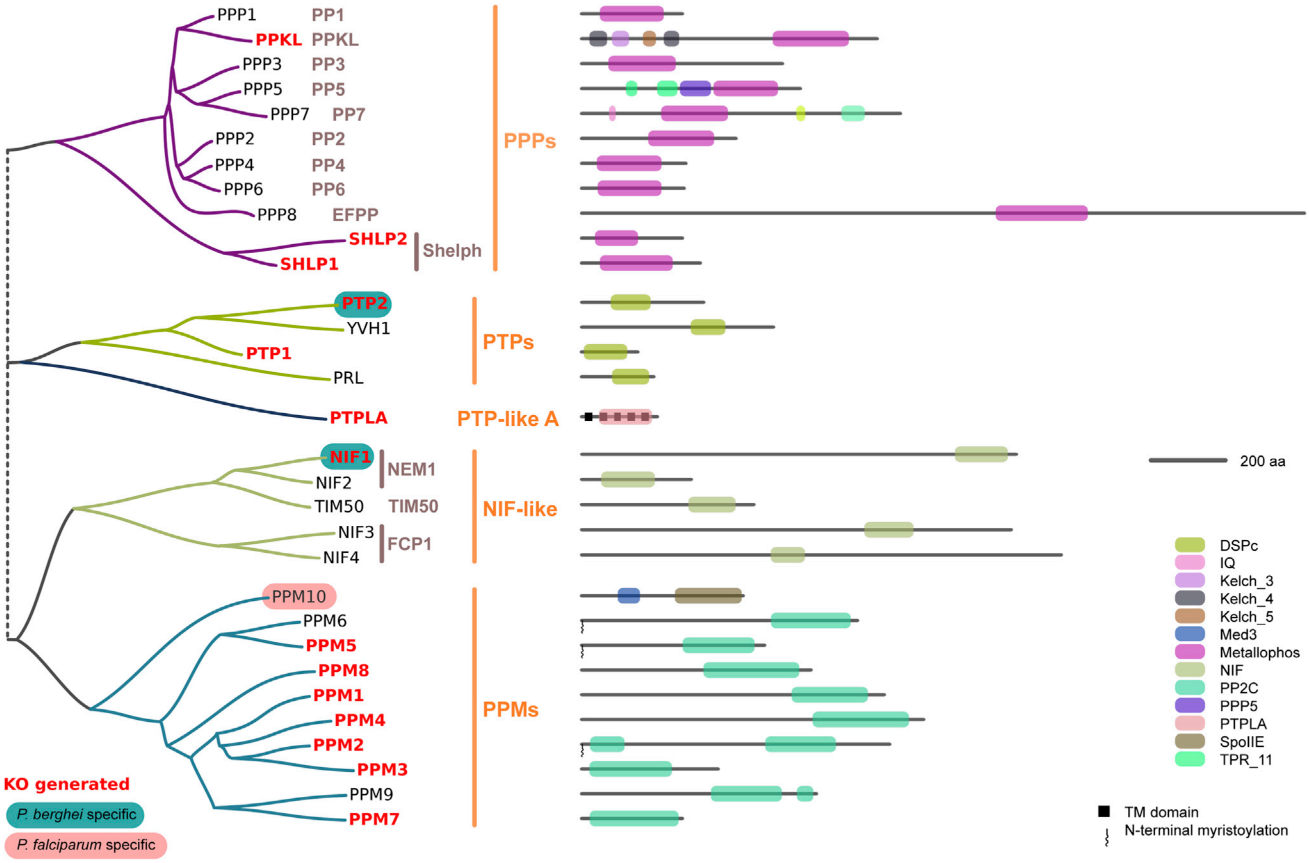

Figure 1. The *Plasmodium* Phosphatome

Schematic phylogenetic tree and domain architectures for the PPs of *P. berghei* ANKA and *P. falciparum* 3D7 showing family and subfamily classification. Proteins encoded in only one species are highlighted. Deletion mutants obtained are shown in bold red text. Domain architecture for *P. falciparum* protein is shown unless no ortholog exists (PTP2, NIF1). See also Figure S1.

nuclear, and cytoplasmic (Figures 2A and 2B), with a number falling into more than one class (nucleocytoplasmic). However, only two PPs were found to be localized to a single region throughout the entire life cycle: PPM4 (the nucleus) and NIF2 (the cytoplasm). Furthermore, a number were expressed only at specific stages. For example, PTP1 was found to be completely absent in male gametocytes but present at all other stages, whereas YVH1 was absent in ookinetes (Figure 2B).

Of the PPs localized to a precise region of the parasite body, PTP1 was found to be localized to the apical tip of mature ookinetes, suggestive of a role in apical polarity, whereas PPM5 was found to show both nuclear and membrane localization in zygotes but was mostly absent from the cytoplasm (Figure 2B).

Previous studies have localized a small number of *P. falciparum* PPs during asexual stages. PfPP2C (PPM2 in our study) has been shown to be highly expressed in rings, trophozoites, and schizonts (Mamoun et al., 1998), whereas PfYVH1 has been found to be active and undergo nucleocytoplasmic shuffling during periods of high transcriptional activity (Kumar et al., 2004), both of which are consistent with our study. Furthermore, PfYVH1 has been shown to interact with the cell-cycle-regulatory protein pescadillo (PES) (Kumar et al., 2004). However, PfPRL was localized to the ER and non-ER punctuate structures in *P. falciparum* asexual stages (Pendyala et al., 2008), whereas we found it exclusively in the zygotic cytoplasm, suggesting species-specific differences in PP localization and, perhaps, activity.

Gene Disruption Analysis Suggests Nearly Half of the *P. berghei* PPs Are Redundant during Erythrocytic Stages

To identify PPs that are functional during *P. berghei* asexual blood stage development, we attempted to delete systematically each of the 30 *P. berghei* PPs using double homologous recombination (Figure S3A). A total of 178 individual gene deletion attempts were performed (at least 4 per gene; Table S1), resulting in successful deletion of 14 *PP*s (47%) as determined by detailed genotype analysis (Figures 2C and S3B; Table S1). Independent clones for each mutant were produced at least twice from independent experiments. Overall, we did not observe a reduced growth phenotype in mice for any of the mutants, suggesting redundancy for each during asexual blood stage development. As a result, we screened each mutant to see whether they are essential during sexual stage development and transmission in *Anopheles stephensi* mosquitoes.

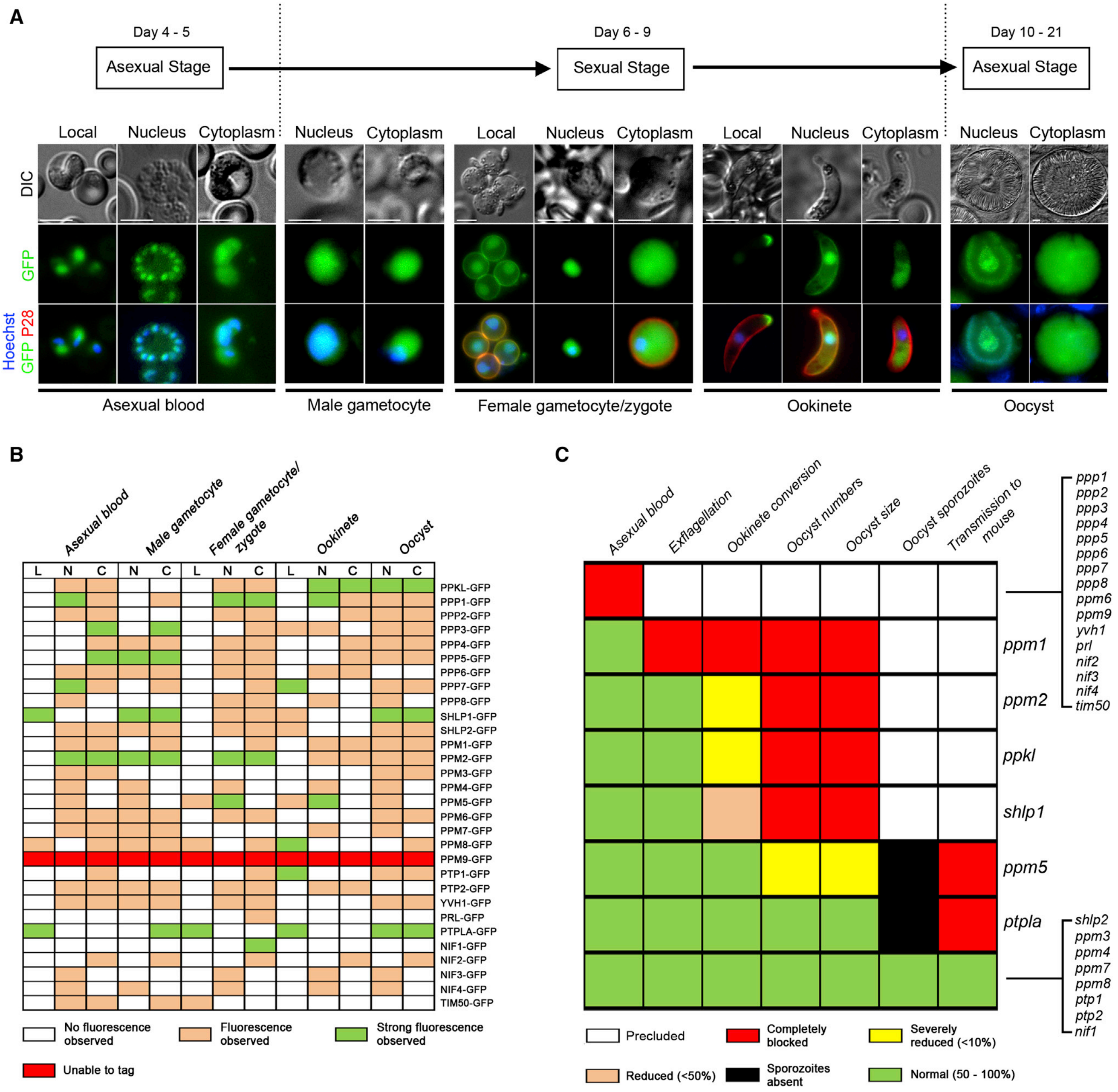

Figure 2. PP Expression and Phenotypic Analysis of 14 PP Mutants

(A) Localization of representative PP-GFP classified into three categories: present only in nucleus (Nucleus), diffuse staining (Cytoplasm), or localized to specific cellular domain (Local). Scale bar, 5 μm. Green, GFP; blue, Hoechst; red, Cy3 P28 staining.

(B) PP-GFP expression in five key developmental stages. N, nucleus; C, cytoplasm; L, local/heterogeneous.

(C) Representation of phenotypic analysis. See also Figures S2 and S3, Table S1, Table S2, and Table S5.

Phenotypic Analysis Suggests Six *P. berghei* PPs Are Required for Parasite Sexual Development/Sporogony and Differentiation in the Mosquito

To assess their role during sexual stage development, we initially screened the ability of the mutants to form viable ookinetes in vitro. A number of PKs are known to have an essential role at this stage, with CDPK4, MAP2, and SRPK being essential for microgamete production; NEK2, NEK4, PK7, and GAK being essential regulators of zygote development; and CDPK1 and CDPK3 required for ookinete gliding motility (Sebastian et al., 2012; Siden-Kiamos et al., 2006; Tewari et al., 2010).

For ten mutants, ookinete conversion showed no overt differences from wild-type parasites (Figure 2C; Table S2), suggesting no role for these PPs up to this stage. However, four mutants were strongly affected in their ability to produce viable ookinetes in vitro. Two of these, Δ*ppkl* and Δ*shlp1*, have been described

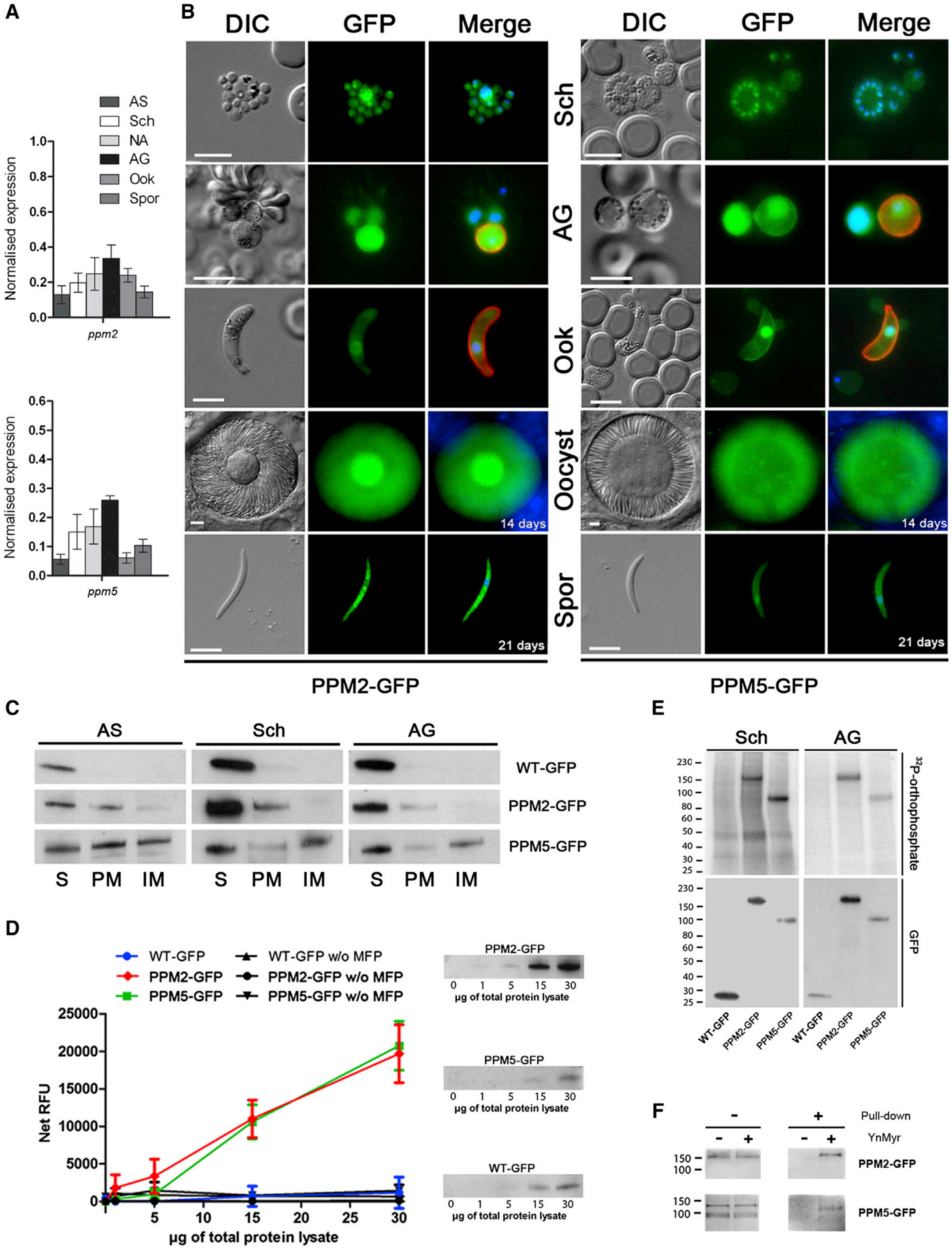

Figure 3. PPM2 and PPM5 Expression, Phosphatase Activity and Phosphorylation, and *N*-Myristoylation Status

(A) Wild-type RNA expression of *ppm2* (upper panel) and *ppm5* (lower panel). Error bar ± SEM, n = 3. AS, asexual blood stages; Sch, schizonts; NA, nonactivated gametocytes; AG, activated gametocytes; Ook, ookinetes; Spor, sporozoites.

(B) Expression of PPM2-GFP (left) and PPM5-GFP (right). Merge is the composite of Hoechst to detect the nuclei, GFP and Cy3 P28 for sexual stages. Scale bar, 5 µm.

(C) Anti-GFP western blot of soluble (S), peripheral membrane (PM), and integral membrane (IM) fractions from parasite lysates.

(D) (Left) Phosphatase activity in parasite lysate immunoprecipitates. Error bar ± SEM, n = 3. (Right) Anti-GFP western blot from corresponding lysates.

(legend continued on next page)

previously and were shown to be essential for ookinete differentiation and oocyst development, respectively (Guttery et al., 2012; Patzewitz et al., 2013). One mutant, Δ*ppm1*, formed morphologically normal gametocytes (data not shown) and produced macrogametes that emerged from their host cells expressing the activation marker P28, but did not produce any ookinetes (Figure 2C; Table S2). Therefore, we assessed whether microgamete formation was affected in this mutant and found that exflagellation was completely blocked (Figure 2C; Table S2). Another mutant, Δ*ppm2*, showed grossly reduced macrogamete numbers (<30% of wild-type controls) and subsequent ookinete conversion (<5% of the population), with the majority of the population being retorts, suggesting this PP is essential for ookinete differentiation similar to PPKL (Figure 2C; Table S2). This was surprising, as the *P. falciparum* ortholog of PPM2 (PfPP2C) is refractory to deletion (Mamoun and Goldberg, 2001). One other mutant, Δ*ppm5*, produced viable ookinetes similar to wild-type controls, but was markedly reduced in its ability to produce fully formed oocysts (reduced in number and size with no sporozoite development) 14 days postinfection. Finally, Δ*ptpla* parasites produced equivalent numbers of oocysts compared to wild-type; however, no sporozoites were produced. The remaining eight mutants showed no observable defects throughout sexual development, sporogony, and transmission to mice (as assessed by blood smears 4–10 days postinfection) (Figure 2C; Table S2), suggesting functional redundancy for these PPs during these stages of the life cycle. However, the role of any PP during liver-stage development cannot be discounted, as we did not directly assess this stage of the life cycle in this study. In this study, all eight mutants gave rise to blood stage infection with a 4–5 day prepatent period in mosquito biteback experiments, suggesting little effect during liver stage development.

Systematic functional analysis of the *P. berghei* kinome revealed a number of PKs to be essential for parasite development in the mosquito (Tewari et al., 2010). In contrast to these kinases, which have diverged through numerous variations of the catalytic subunits, the PPs contain a comparatively small number of highly conserved catalytic subunits with supposedly nondiscriminatory substrate specificity in vitro. However, more recent studies have given the PPs far greater specificity due to their assembly with hundreds of regulatory subunits, giving them the ability to recognize environmental cues and coordinate highly complex and specific chains of events. Therefore, the refractory nature of the PPPs in this study is not surprising. They are known to form multimeric holoenzyme complexes with a wide variety of regulatory subunits that bestow substrate selectivity and direct subcellular localization of the catalytic subunit, which are highly regulated and specific (Virshup and Shenolikar, 2009). Similarly, the inability to delete three *FCP*s (NIF2, NIF3, and NIF4) is not surprising, as the common substrate of FCPs is the C-terminal domain of RNA polymerase II (Yeo et al., 2003). Finally, *yvh1* is essential in fungi (Sakumoto et al., 2001); therefore it is not surprising to find it refractory to deletion here. In contrast, the PPMs are generally thought to exist as monomers whose catalytic domains are highly conserved, and as we were able to successfully delete seven of nine (78%) of the *PPM*s, this suggests a mechanism of functional compensation by other PPMs.

We have shown here that, as expected, a number of PPs are essential for parasite sexual development/sporogony at similar stages to a number of PKs (Tewari et al., 2010). Whether they have similar substrates (or indeed target each other) is yet to be determined.

Two Unique Plasmodial PPs Are Differentially Localized, Show Phosphatase Activity, and Are Phosphorylated and Myristoylated

Protein phosphorylation and *N*-myristoylation are important posttranslational mechanisms regulating protein function. PPM2 and PPM5 are suggested to be regulated by phosphorylation (Treeck et al., 2011), were recently identified as *N*-myristoylated in *P. falciparum* (Wright et al., 2014), and are shown here to have essential functions during sexual development. Therefore, we examined their transcription and expression profile, and *N*-myristoylation and phosphorylation status.

qRT-PCR and C-terminal GFP tagging together demonstrated that both PPs are present throughout the life cycle, confirming previous studies (Hall et al., 2005; Le Roch et al., 2003) (Figures 3A and 3B). Subcellular fractionation of blood stage parasites confirmed a cytoplasmic and peripheral membrane localization for PPM2-GFP, whereas PPM5-GFP was present in all three fractions analyzed (Figure 3C), consistent with its diffuse localization in microscopic analyses (Figure 3B). PP activity assays using 3-O-methylfluorescein phosphate (MFP) as a substrate (Patzewitz et al., 2013) confirmed PPM2 and PPM5 to be active PPs (Figure 3D). Computational homology modeling of PPM2 and PPM5 confirms the presence of metal coordinating residues and showed the presence of long Asn-rich loops within the PP domain (Figure S4). Such Asn-rich low-complexity regions are often seen in *Plasmodium* proteins, and their function remains obscure. Although most *P. berghei* PPs do not have extensive Asn-rich regions, they occur in some other PPs, for example PPP8. The loops in PPM2 and PPM5 are atypical in that they are >100 residues and are located within the catalytic domain. For PPM2 and PPM5, the loop regions are located at different positions in the catalytic domain. However, PPM2 and PPM5 have one loop in common that protrudes close to the active site (Figure S4), and this loop is likely to affect interactions with substrates, and might be involved in substrate selection.

Analysis of PPM2-GFP and PPM5-GFP using ^{32}P-orthophosphate labeling in vivo (Guttery et al., 2012) confirmed them to be phosphorylated in schizonts (as in *P. falciparum* [Treeck et al., 2011]) and activated gametocytes (Figure 3E), further implicating phosphorylation as a key regulator of PP activity (Mochida and Hunt, 2012). PPM2 and PPM5 proteins from schizonts were also labeled by the myristic acid analog tetradec-13-ynoic acid (YnMyr) (Heal et al., 2012) (Figure 3F), confirming previous *P. falciparum*

(E) In vivo phosphorylation. (Upper panel) [^{32}P]-phosphorylation of immunoprecipitated GFP-proteins from parasite lysates. (Lower panel) Corresponding western blot. Protein markers are to the left.
(F) Parasite lysates labeled with YnMyr (+) and controls without labeling (–) were run directly (– pull-down) or following affinity purification (+) and detected with anti-GFP antibody. Protein markers are shown to the left. See also Figure S4 and Table S6.

myristome studies and highlighting these PPs as substrates of *N*-myristoyl transferase, which is currently being investigated as a potential drug target in malaria (Wright et al., 2014).

A myristoyl group is only a weak membrane anchor, allowing membrane association in a reversible and regulated manner, in agreement with the presence of PPM2 and PPM5 in soluble and membrane-associated fractions. PPM5 has a cluster of positive charges close to the N terminus (underlined residues in Figure S4F), and in other proteins, such as Src family kinases, these clusters are known to stabilize membrane anchoring of the protein. PPM2 lacks such a cluster, or other hydrophobic groups, and therefore membrane anchoring of PPM2 might be promoted through additional factors.

PPM2 Is Essential for Gametocyte Sex Allocation and Ookinete Differentiation, whereas PPM5 Modulates Oocyst Development

As PPM2 and PPM5 were identified as key regulators of sexual/sporogonic development, we performed an in-depth analysis to examine at what stage the proteins are essential. Although exflagellation in Δ*ppm2* was comparable to wild-type controls and Δ*ppm5* parasites (Figure 4A), gametocytaemia and subsequent female gamete production were severely reduced (Figure 4A). This reduced female gamete count was reflected in an altered female:male gametocyte ratio of approximately 1:1 in Δ*ppm2* parasites, compared to 3:1 observed in wild-type controls (Figure 4A). Sex allocation in *Plasmodium* is inherently female biased (~3.6 female gametocytes to 1 male; Robert et al., 1996) in order to balance the greater number of male gametes per gametocyte, hence maximizing fertilization and transmission success. Furthermore, male gametocytes have been shown to have higher longevity in the blood meal (Reece et al., 2003) and are able to recognize genetically identical kin (Reece et al., 2008), both of which may affect sex ratios. Therefore, the reduction of macrogametes in vitro, altered sex allocation, and decreased infectivity in Δ*ppm2* parasites could suggest that the protein is vital for the response of *Plasmodium* parasites to environmental factors, or that longevity of the mutant female gametocytes is affected, resulting in lower numbers. Alternatively, PPM2 could play a role in recognition of genetically identical kin, with its absence resulting in a switch to a 1:1 ratio.

Zygote (ookinete) maturation in vitro was severely affected in Δ*ppm2* lines compared to wild-type, with the majority of the mutant population stalled by stage II of ookinete development, and oocyst development was completely ablated (Figures 4B and 4C). In contrast, Δ*ppm5* ookinetes developed normally and were motile (Figures 4B and S5A). However, oocyst development was severely affected, with reduced size (<40%) and numbers (<5%) compared to wild-type controls (Figures 4C and S5B) despite the fact that Δ*ppm5* ookinetes contained a normal DNA content (4N) similar to wild-type (Figure S5C). Analysis of Δ*ppm2* parasites revealed a reduced but highly variable DNA content (Figure S5C), suggesting meiotic DNA replication was initiated but aborted prior to completion, as observed previously in Δ*misfit* parasites (Bushell et al., 2009) and to some extent in *nek4* mutant parasites (Reininger et al., 2005). No sporozoites were present 14 days postinfection, and no transmission via biteback was observed (Table S2), confirming that this PP is essential during early oocyst development. Detailed analysis of ookinete differentiation showed that most of the Δ*ppm2* population did not progress to stage I, a few were arrested at stage II (Janse et al., 1985), and after 24 hr only a fraction of the population (<0.5%) had developed to stage VI, compared to >40% of wild-type controls (Figure 4D). This suggests multiple stages of essentiality for PPM2, i.e., sex allocation in developing gametocytes, and regulation of maturation, differentiation, and morphological development from stage II to III of ookinete development. The importance of this protein is strengthened by the fact that PfPP2C dephosphorylates translation elongation factor 1β (EF-1β) (Mamoun and Goldberg, 2001), with mutants of EF-1β showing severe growth defects and sensitivities to elongation inhibitors in yeast (Carr-Schmid et al., 1999).

Δ*ppm2* and Δ*ppm5* Are Defective along the Female and Male Lineages, Respectively

In genetic crosses with lines deficient in either male (Δ*map2*) or female (Δ*nek2*/Δ*nek4*) gametes (Reininger et al., 2005, 2009; Tewari et al., 2005), Δ*map2* (but not Δ*nek2* gametes) formed mature ookinetes when crossed with Δ*ppm2* mutants (Figure 4E), indicating that PPM2 is necessary for female gamete formation, as seen for PPKL (Guttery et al., 2012). Crossing Δ*ppm5* with Δ*nek4* lines rescued the phenotype, with mature sporozoite-containing oocysts 14 days postinfection (Figure 4F), indicating the requirement for a functional *ppm5* gene in the male line. The essential requirement for a functional male lineage during oocyst development is similar to properties of the *misfit* gene, as is its stage of essentiality (Bushell et al., 2009). Whether or not MISFIT is a target of PPM5 requires further study.

Ultrastructure Analysis Shows Defects in Ookinete Formation and Differentiation in Δ*ppm2* Mutants, whereas Δ*ppm5* Ookinetes Showed Partial Defects in Microneme Formation

We analyzed Δ*ppm2* gametocytes and stage-arrested ookinetes, and Δ*ppm5* ookinetes, using transmission electron microscopy (TEM). The Δ*ppm2* gametocyte samples contained fewer macrogametes, but the gametocyte (male and female) morphology was similar to that of wild-type (Figure S5D). In contrast to crescent-shaped wild-type ookinetes (Figure 4Gi), the vast majority of Δ*ppm2* ookinetes were arrested at the early retort stage II, with a bulbous shaped body and cytoskeletal abnormalities (Figure 4Gii). In the few ookinetes that did mature, the apical membrane complex appeared normal, and late-stage features such as the crystalline body were present, but there were few or no micronemes (Figure 4Gii), as seen in SHLP1 mutants (Patzewitz et al., 2013). Δ*ppm5* ookinetes were crescent shaped with the apical membrane complex seen in wild-type parasites (Figures 4Giii and 4Giv), but there was marked variability in the number of micronemes, ranging from normal to none (Figures 4Giii and 4Giv). This defect could explain the formation of only a small number of abnormal oocysts that are unable to produce sporozoites.

Global Transcript Analysis of Δ*ppm2 and* Δ*ppm5* Reveals Dysregulation of Genes Involved in Reversible Phosphorylation, Membrane Structure, Motility, and Invasion

Transcription was analyzed by strand-specific RNA-sequencing (RNA-Seq) across relevant life cycle stages, and identified

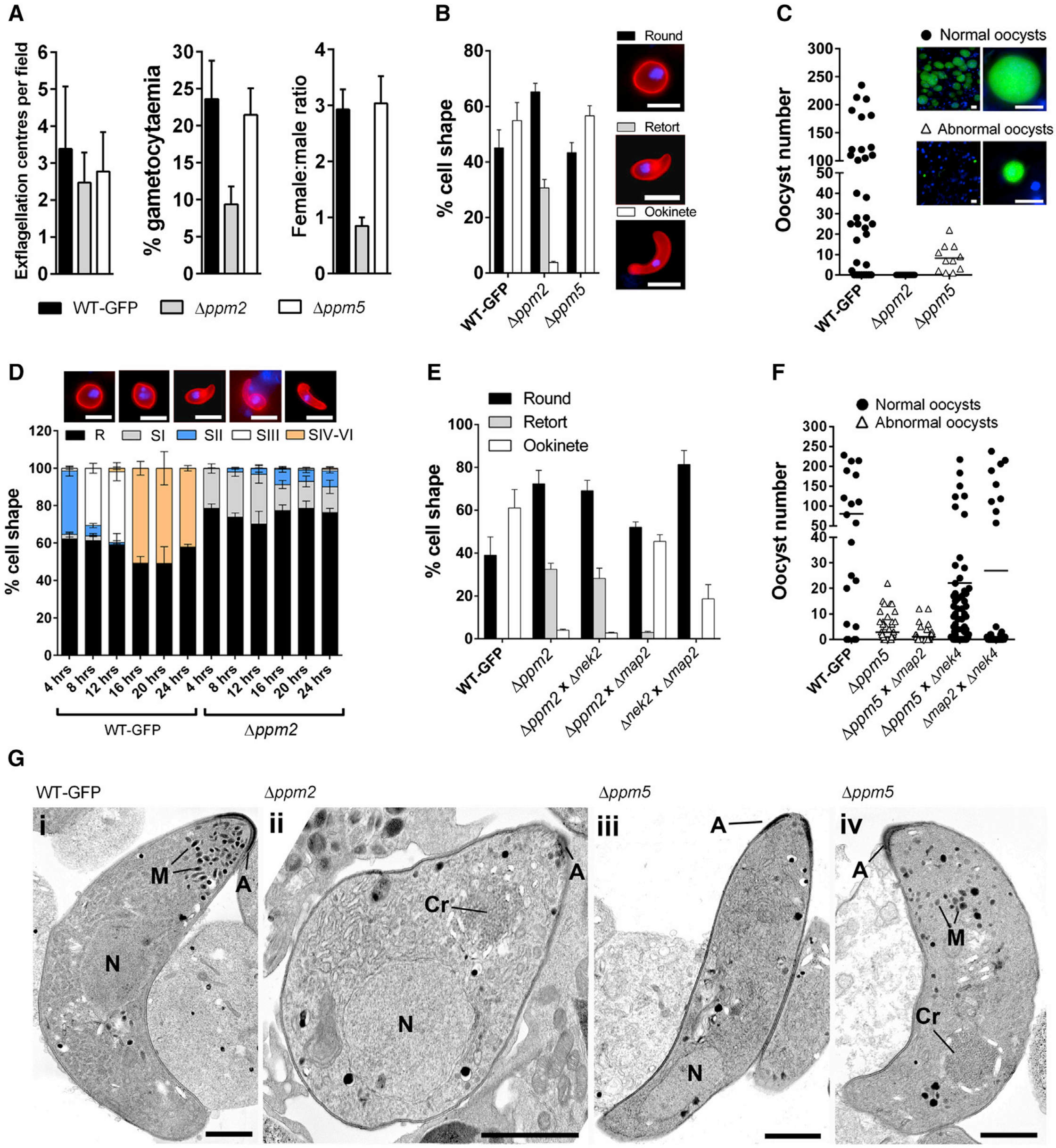

Figure 4. Phenotypic and Ultrastructure Analysis of Δ*ppm2* and Δ*ppm5*

(A) Exflagellation (left), gametocytaemia (middle), and gametocyte sex allocation (right) of WT-GFP, Δ*ppm2*, and Δ*ppm5* parasites. Error bar ± SD; n = 3.

(B) Ookinete conversion in WT-GFP, Δ*ppm2*, and Δ*ppm5* parasites. Error bar ± SD; n = 3.

(C) Average number of oocysts per mosquito gut. Scale bar, arithmetic mean; n = 60. Infection prevalence was 81% for wild-type, 0% for Δ*ppm2*, and 88% for Δ*ppm5*. Scale bar, 50 μm.

(D) Ookinete differentiation. Morphologies used for scoring are given above the graph and are previously described (Janse et al., 1985). Error bar ± SD, n = 3. Scale bar, 5 μm.

(E) Ookinete conversion after genetic crossing. Error bar ± SD; n = 3.

(F) Genetic complementation. Scale bar, arithmetic mean; n = 60.

(G) (Gi) TEM of a longitudinal section through a wild-type crescent shaped ookinete. (Gii) Section through a Δ*ppm2* retort showing the bulbous shape of the parasite but with normal structures in the cytoplasm. Note the very few micronemes. (Giii) Example of a Δ*ppm5* ookinete showing the crescent shape but no micronemes. (Giv) Δ*ppm5* ookinete containing few micronemes. For all panels, A, apical membrane complex; N, nucleus; Cr, crystalline body; M, micronemes. Scale bar, 1 μm. See also Figure S5.

changes in key genes involved in zygote/ookinete development and transmission when the Δ*ppm2* and Δ*ppm5* lines were compared to wild-type (Figures 5A and 5B; Table S3 and Table S4).

We analyzed transcription in two Δ*ppm2* life stages, with particular emphasis on genes related to zygote/ookinete structure, motility, and invasion. Several gene clusters significantly affected in Δ*ppm2* schizonts and activated gametocytes were

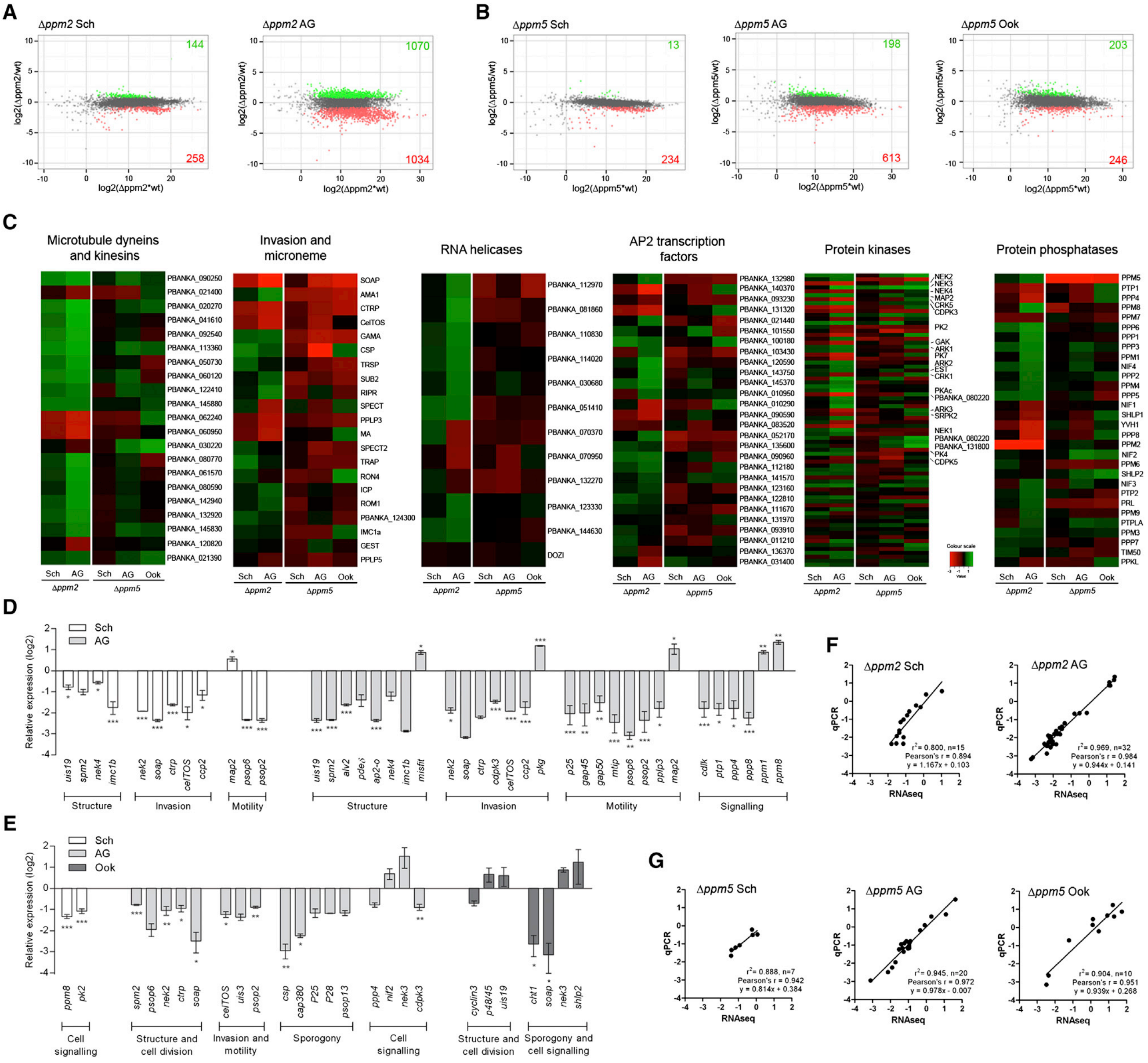

Figure 5. Global Transcriptional Analysis of Δ*ppm2* and Δ*ppm5* by RNA-Seq

(A and B) Ratio-intensity scatterplots of normalized FPKM values for each stage of mutant development. Log_2 fold change between wild-type and mutant (y axis) and the average FPKM value (x axis).

(C) Log_2 fold change in Δ*ppm2* and Δ*ppm5* at different life cycle stages. Functional groups were inferred from annotations available in GeneDB (http://www.genedb.org/Homepage). Genes are arranged in order of significance (in relation to regulation) in each sample and in the total data set. Full gene list and heatmap order are shown in Table S4.

(D and E) qRT-PCR of a variety of genes (based on data from RNA-Seq) in (D) Δ*ppm2* and (E) Δ*ppm5* parasites compared to wild-type controls. Error bar ± SEM, n = 3 biological replicates. Schizonts, Sch; activated gametocytes, AG; ookinetes, Ook. Student's t test, *p < 0.1, **p < 0.05, ***p < 0.001.

(F and G) qRT-PCR validation of the RNA-Seq data using Log_2 values. See also Figure S6, Table S3, Table S4, and Table S6.

identified (Figure 5C; Table S4), which may be responsible for the pleiotropic nature of the Δ*ppm2* phenotype. Changes included downregulation of genes important for membrane structure (*kinesins*, *dyneins* [Fowler et al., 2001], *imc1b* [Tremp et al., 2008]), cell-cycle regulation (*pdeδ* [Moon et al., 2009] and *nek4* [Reininger et al., 2005]), ookinete motility (*gap45* and *gap50* [Baum et al., 2006]), and invasion (*soap*, *ctrp*, and *cdpk3* [Angrisano et al., 2012]) (Figures 5C and 5D; Table S3 and Table S4). Members the AP2 transcription factor family implicated in gametocyte and zygote formation (including *ap2-o*) (Painter et al., 2011; Yuda et al., 2009) were also differentially regulated (Figures 5C and 5D; Table S3 and Table S4). We also observed a general increase of RNA helicases in activated gametocytes. Furthermore, the few mature ookinetes that did form had

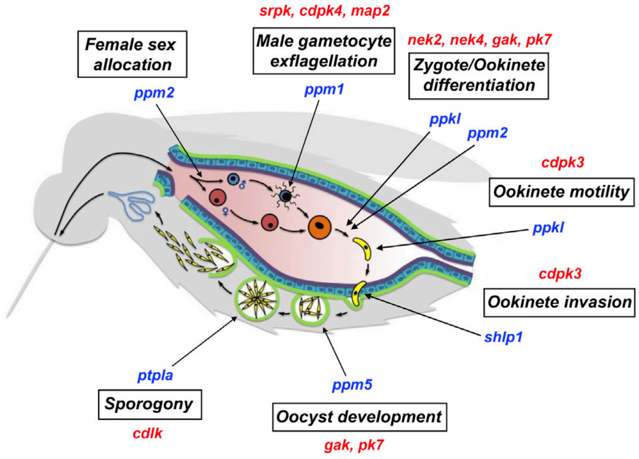

Figure 6. Summary of PP Function throughout the *P. berghei* Life Cycle

PPs with essential functions in the mosquito are highlighted (blue). Protein kinases essential at similar stages (Tewari et al., 2010) are highlighted in red. See also Table S2.

reduced *gap50* and *soap* expression, potentially limiting motility, invasion, and oocyst formation (Figure 5D; Table S3). Global Gene Ontology (GO) analysis revealed enrichment of multiple categories such as "microtubule cytoskeleton" and "microtubule-based movement" in Δ*ppm2* lines (Figures 5C, 5D, and S6A; Table S3 and Table S4).

We analyzed transcription in three developmental stages (schizonts, activated gametocytes, and ookinetes) of Δ*ppm5* parasites compared to wild-type. Although the Δ*ppm5* phenotype was first observed in oocysts (Figure 4C), genetic crossing indicated that the mutation affects the male gametocyte (Figure 4F). This is supported by the RNA-Seq analysis, since several gene clusters related to the Δ*ppm5* phenotype were differentially expressed (Figure 5C; Table S4). Genes associated with microneme development (*soap* and *ctrp*), oocyst development and sporogony (*cap380* [Srinivasan et al., 2008] and *csp* [Ménard et al., 1997]) were downregulated in activated Δ*ppm5* gametocytes (Figures 5C and 5E; Table S3). GO analysis revealed enrichment of "microneme," "cytoplasm," and "apical part of cell" in all stages of Δ*ppm5* analyzed (Figure S6A), corresponding well with the phenotype.

In activated Δ*ppm2* gametocytes, 39 PKs, including ones essential for zygote development (*nek2*, *nek4*, *pk7*, and *gak*) and ookinete motility (*cdpk3*) (Tewari et al., 2010), were differentially expressed, whereas 17 PPs, including those associated with ookinete development such as *shlp1*, were differentially expressed (Figures 5C and 5D; Table S4), suggesting PPM2 has a signaling role in female development. In contrast, only a few PKs (such as *nek2* and *nek3*) and PPs (*ppm6* and *ppm8*) were differentially regulated in activated Δ*ppm5* gametocytes.

For both mutants, the RNA-Seq data were validated using qRT-PCR for selected genes (Figures 5D and 5E) and showed a good correlation (Figures 5F and 5G). It should be noted that transcriptional changes in the mutants probably reflect phenotypic changes in the cell state at a given life cycle stage, and cannot be used to infer a direct function of the PP, owing to the likely complexity of the signaling pathways.

To provide additional support for the differential gene expression patterns observed in Δ*ppm2* and Δ*ppm5* PP mutants, we compared protein-protein interaction partners of PPM2 and PPM5 derived from growth perturbation data sets of *P. falciparum* (Hu et al., 2010) and the differentially expressed genes seen in Δ*ppm2* and Δ*ppm5* across the studied life stages (Table S3). We provide evidence for significant numbers of differentially expressed genes in Δ*ppm2* and Δ*ppm5* mutants directly interacting with PPM2 and PPM5, respectively (Figure S6C). Approximately 30% (top 50% network, p value = 0.0002) of the differentially expressed genes in Δ*ppm2* schizonts and activated gametocytes were also found to directly interact with PPM2 (Figures S6C and S6D; Table S3). Several ookinete motility proteins and PKs (CDPK3 and PK7) were all found to interact directly with PPM2 (Figure S6).

In Δ*ppm5*, approximately 28% (top 50% network, p value = 0.002) of the differentially expressed genes in the three developmental stages of Δ*ppm5* were found to directly interact with PPM5 (Figures S6B–S6D; Table S3). For example, the data suggest that CDPK3, CDPK5, and SHLP2 interact directly with PPM5. Finally, it is important to note that while our network analysis indicates to an extent that the transcriptional changes we observe are related to the knocked out PP, the precise nature of these interactions remains to be elucidated experimentally.

Conclusions

This systematic functional analysis of the *P. berghei* phosphatome shows that PPs have many essential functions during the *Plasmodium* life cycle (particularly during sexual and sporogonic development), as found with the plasmodial kinome (Tewari et al., 2010) and summarized in Figure 6. In contrast to the PKs, which have diverged through evolution of the catalytic subunits, PPs comprise a small number of highly conserved catalytic subunit families. Specificity is conferred in part by association with many regulatory subunits, allowing PPs to respond to environmental cues and coordinate complex and specific chains of events (Virshup and Shenolikar, 2009). It is well known that signal transduction pathways are not simply "switched on" by kinases and "switched off" by PPs; rather, it is often a dynamic balance between the two, with multiple kinases and PPs contributing to certain stage-specific pathways. A number of PPs were shown here to be essential at similar stages of the life cycle to certain kinases (e.g., MAP2 and PPM1; PK7 and PPM5; see summary, Figure 6), with transcriptome sequencing further demonstrating that reversible protein phosphorylation is likely to be a complex and highly coordinated process that regulates sexual and sporogonic development. Whether these kinases and PPs truly act in a complementary manner and whether or not changes in transcription profiles are well downstream of the point at which these enzymes act will be determined in future studies.

As nearly half of the PP genes are nonessential for *P. berghei* blood stages, a large part of the phosphatome can be deprioritised as targets for drugs directed against this life cycle stage. Most drug discovery studies in mammalian systems have focused on PTPs with varying success (De Munter et al., 2013), although there are studies (Moorhead et al., 2007) proposing small molecule inhibitors of PPs to target diabetes, obesity, and other human diseases (De Munter et al., 2013; Zhang et al., 2013). There is also great interest in understanding the

role of PPs as regulators of the cell cycle and mitosis, and hence as targets for cancer therapy (Barr et al., 2011; Mochida and Hunt, 2012). Overall, protein PPs with unique roles in disease may be promising targets for therapeutic intervention, and, following this study, various multifunctional approaches can be used in the future to identify the potential of unique malarial PPs as part of a drug discovery program.

EXPERIMENTAL PROCEDURES

Ethics Statement

All animal work has passed an ethical review process and was approved by the United Kingdom Home Office. The project license number is 40/3344.

Animals

Six- to eight-week-old female Tuck-Ordinary (TO) outbred mice (Harlan) were used for all experiments.

Comparative Bioinformatics

PPs from a variety of families were identified in the predicted proteomes of *P. berghei*, *P. falciparum*, and 44 other diverse eukaryotes using HMMER3. For classification, full-length proteins were clustered and large-scale maximum-likelihood phylogenies built from alignments of PP domains (MAFFT6.24) with statistical support from the approximate Likelihood Ratio Test as implemented by PhyML3.0. Possible myristoylation was predicted using *N*-Myristoyltransferase (NMT) Myristoylator (Bologna et al., 2004).

C-Terminal GFP Fusion and Gene Deletion

To C-terminally tag PPs with GFP, a single homologous recombination strategy at the endogenous locus was used (Guttery et al., 2012). For systematic gene deletion analyses, a traditional double homologous recombination approach was used (Tewari et al., 2010). Successful integration of target sequences was confirmed using genotype analysis. For each mutant, at least two clones were produced from independent transfections (see Table S5 for primer sequences).

Phenotypic Analysis

Phenotypic analyses were performed as previously described (Guttery et al., 2012; Tewari et al., 2010). Asexual proliferation and gametocytogenesis were analyzed using blood smears. Gametocyte activation, zygote formation, and ookinete conversion rates were monitored using in vitro cultures and the surface antigen P28. For mosquito transmission, triplicate sets of 20–60 *Anopheles stephensi* were used.

PPM2 and PPM5 Phosphatase Assay, Subcellular Fractionation and In Vivo Phosphorylation

Blood aliquots from parasite-infected mice were processed for protein purification and PP assay. Soluble (hypotonic lysis), peripheral membrane (carbonate soluble), and integral membrane (carbonate insoluble) fractions were analyzed by western blotting for subcellular fractionation. For in vivo phosphorylation, purified activated gametocytes and purified schizonts were metabolically labeled with 3–5 MBq ^{32}P-orthophosphate for 30 min and processed for phosphorylation assay (Patzewitz et al., 2013).

Metabolic Labeling and Purification of *N*-Myristoylated Proteins

In vivo myristoylation of PPM2 and PPM5 was assessed using the alkyne-tagged myristic-acid analog YnMyr and bio-orthogonal ligation to label all proteins that had incorporated the YnMyr probe with a biotinylated capture reagent ligated to the alkyne tag, which then allowed the selective affinity pull-down of tagged proteins with a streptavidin-conjugated resin (Wright et al., 2014).

Transcriptome Sequencing, qRT-PCR, and Interaction with Phosphatase Network Analysis

qRT-PCR was performed from 250 ng of total RNA using *hsp70* and *seryl-tRNA synthetase* as references (see Table S6 for primer sequences). Strand-specific mRNA sequencing was performed from two to four biological replicates of total RNA using TruSeq Stranded mRNA Sample Prep Kit LT (Illumina). Libraries were sequenced in Illumina Hiseq with paired-end 100 bp read chemistry. Strand-specific RNA-Seq paired-end reads were mapped onto the *P. berghei* ANKA genome (PlasmoDB-9.2) using TopHat version 2.0.8 (Trapnell et al., 2009), then quantified and compared across different samples using Cuffdiff version 2.1 (Trapnell et al., 2013). Differentially expressed genes from Δ*ppm2* and Δ*ppm5* were overlapped with interaction partners for both PP networks derived previously in *P. falciparum* (Hu et al., 2010).

Statistical Analyses

Statistical analyses were performed using GraphPad Prism (GraphPad Software). For relative gene expression, a Student's t test was used.

A complete description of the materials and methods used in this study is provided in the Supplemental Experimental Procedures.

ACCESSION NUMBERS

Transgenic parasites lines generated in the study are deposited to the Rodent malaria database at http://www.pberghei.eu/.

SUPPLEMENTAL INFORMATION

Supplemental Information includes six figures, six tables, and Supplemental Experimental Procedures and can be found with this article at http://dx.doi.org/10.1016/j.chom.2014.05.020.

AUTHOR CONTRIBUTIONS

R.T. and A.A.H. conceived of and designed the study. R.T., D.S.G., B.P., D.B., R.J.W., S.W., E.-M.P., and U.S. performed the functional analysis. B.W. performed the phylogenetic analyses. D.J.P.F. performed the TEM experiments. A. Ramaprasad, A.M.A.H.M., and A.P. provided the RNA-Seq and protein interactome data analysis. A. Radhakrishnan and S.T.A. performed the computational structural analyses. M.H.W. and E.W.T. performed the myristoylation assays. D.S.G., B.P., D.J.P.F., A. Ramaprasad, A.P., A.M.A.H.M., B.W., R.J.W., A.A.H., and R.T. analyzed the data. D.S.G., A.A.H., and R.T. wrote the manuscript, and all others corrected and contributed to it.

ACKNOWLEDGMENTS

The project is funded by MRC Investigator Award and MRC project grants to R.T. (G0900109, G0900278, and MR/K011782/1); A.A.H. is funded by the MRC (U117532067 and G0900278) and the EU, FP7 grant agreement 242095 (EviMalar); D.J.P.F. is funded by a Wellcome Trust Equipment Grant; E.W.T. is funded by the MRC (G0900278); B.W. is funded by a BBSRC new investigator research grant (BB/J01477X/1). A.P., A. Ramaprasad, A.M.A.H.M., A. Radhakrishnan, and S.T.A. are funded by KAUST; M.H.W. is funded by the EPSRC. We would like to thank Nigel Halliday, Balázs Szöőr, Sara Sandrini, Peng Gong, and Julie Rodgers for their discussions and assistance.

Received: March 5, 2014
Revised: April 17, 2014
Accepted: May 27, 2014
Published: July 9, 2014

REFERENCES

Angrisano, F., Tan, Y.H., Sturm, A., McFadden, G.I., and Baum, J. (2012). Malaria parasite colonisation of the mosquito midgut—placing the Plasmodium ookinete centre stage. Int. J. Parasitol. *42*, 519–527.

Bannister, L.H., and Sherman, I.W. (2009). Plasmodium. (Chichester: John Wiley and Sons, Ltd).

Barr, F.A., Elliott, P.R., and Gruneberg, U. (2011). Protein phosphatases and the regulation of mitosis. J. Cell Sci. *124*, 2323–2334.

Baum, J., Richard, D., Healer, J., Rug, M., Krnajski, Z., Gilberger, T.W., Green, J.L., Holder, A.A., and Cowman, A.F. (2006). A conserved molecular motor drives cell invasion and gliding motility across malaria life cycle stages and other apicomplexan parasites. J. Biol. Chem. *281*, 5197–5208.

Bologna, G., Yvon, C., Duvaud, S., and Veuthey, A.L. (2004). N-terminal myristoylation predictions by ensembles of neural networks. Proteomics *4*, 1626–1632.

Bushell, E.S., Ecker, A., Schlegelmilch, T., Goulding, D., Dougan, G., Sinden, R.E., Christophides, G.K., Kafatos, F.C., and Vlachou, D. (2009). Paternal effect of the nuclear formin-like protein MISFIT on Plasmodium development in the mosquito vector. PLoS Pathog. *5*, e1000539.

Carr-Schmid, A., Valente, L., Loik, V.I., Williams, T., Starita, L.M., and Kinzy, T.G. (1999). Mutations in elongation factor 1beta, a guanine nucleotide exchange factor, enhance translational fidelity. Mol. Cell. Biol. *19*, 5257–5266.

Cohen, P. (2000). The regulation of protein function by multisite phosphorylation—a 25 year update. Trends Biochem. Sci. *25*, 596–601.

De Munter, S., Köhn, M., and Bollen, M. (2013). Challenges and opportunities in the development of protein phosphatase-directed therapeutics. ACS Chem. Biol. *8*, 36–45.

Doerig, C., Abdi, A., Bland, N., Eschenlauer, S., Dorin-Semblat, D., Fennell, C., Halbert, J., Holland, Z., Nivez, M.P., Semblat, J.P., et al. (2010). Malaria: targeting parasite and host cell kinomes. Biochim. Biophys. Acta *1804*, 604–612.

Fernandez-Pol, S., Slouka, Z., Bhattacharjee, S., Fedotova, Y., Freed, S., An, X., Holder, A.A., Campanella, E., Low, P.S., Mohandas, N., et al. (2013). A bacterial-like phosphatase of malaria possesses tyrosine phosphatase activity and is implicated in the regulation of Band 3 dynamics during parasite invasion. Eukaryot. Cell *12*, 1179–1191.

Fowler, R.E., Smith, A.M., Whitehorn, J., Williams, I.T., Bannister, L.H., and Mitchell, G.H. (2001). Microtubule associated motor proteins of Plasmodium falciparum merozoites. Mol. Biochem. Parasitol. *117*, 187–200.

Guttery, D.S., Poulin, B., Ferguson, D.J., Szőőr, B., Wickstead, B., Carroll, P.L., Ramakrishnan, C., Brady, D., Patzewitz, E.M., Straschil, U., et al. (2012). A unique protein phosphatase with kelch-like domains (PPKL) in Plasmodium modulates ookinete differentiation, motility and invasion. PLoS Pathog. *8*, e1002948.

Hall, N., Karras, M., Raine, J.D., Carlton, J.M., Kooij, T.W., Berriman, M., Florens, L., Janssen, C.S., Pain, A., Christophides, G.K., et al. (2005). A comprehensive survey of the Plasmodium life cycle by genomic, transcriptomic, and proteomic analyses. Science *307*, 82–86.

Heal, W.P., Wright, M.H., Thinon, E., and Tate, E.W. (2012). Multifunctional protein labeling via enzymatic N-terminal tagging and elaboration by click chemistry. Nat. Protoc. *7*, 105–117.

Hu, G., Cabrera, A., Kono, M., Mok, S., Chaal, B.K., Haase, S., Engelberg, K., Cheemadan, S., Spielmann, T., Preiser, P.R., et al. (2010). Transcriptional profiling of growth perturbations of the human malaria parasite Plasmodium falciparum. Nat. Biotechnol. *28*, 91–98.

Janse, C.J., Mons, B., Rouwenhorst, R.J., Van der Klooster, P.F., Overdulve, J.P., and Van der Kaay, H.J. (1985). In vitro formation of ookinetes and functional maturity of Plasmodium berghei gametocytes. Parasitology *91*, 19–29.

Kumar, R., Musiyenko, A., Cioffi, E., Oldenburg, A., Adams, B., Bitko, V., Krishna, S.S., and Barik, S. (2004). A zinc-binding dual-specificity YVH1 phosphatase in the malaria parasite, Plasmodium falciparum, and its interaction with the nuclear protein, pescadillo. Mol. Biochem. Parasitol. *133*, 297–310.

Kutuzov, M.A., and Andreeva, A.V. (2008). Protein Ser/Thr phosphatases of parasitic protozoa. Mol. Biochem. Parasitol. *161*, 81–90.

Le Roch, K.G., Zhou, Y., Blair, P.L., Grainger, M., Moch, J.K., Haynes, J.D., De La Vega, P., Holder, A.A., Batalov, S., Carucci, D.J., and Winzeler, E.A. (2003). Discovery of gene function by expression profiling of the malaria parasite life cycle. Science *301*, 1503–1508.

Mamoun, C.B., and Goldberg, D.E. (2001). Plasmodium protein phosphatase 2C dephosphorylates translation elongation factor 1beta and inhibits its PKC-mediated nucleotide exchange activity in vitro. Mol. Microbiol. *39*, 973–981.

Mamoun, C.B., Sullivan, D.J., Jr., Banerjee, R., and Goldberg, D.E. (1998). Identification and characterization of an unusual double serine/threonine protein phosphatase 2C in the malaria parasite Plasmodium falciparum. J. Biol. Chem. *273*, 11241–11247.

Ménard, R., Sultan, A.A., Cortes, C., Altszuler, R., van Dijk, M.R., Janse, C.J., Waters, A.P., Nussenzweig, R.S., and Nussenzweig, V. (1997). Circumsporozoite protein is required for development of malaria sporozoites in mosquitoes. Nature *385*, 336–340.

Mochida, S., and Hunt, T. (2012). Protein phosphatases and their regulation in the control of mitosis. EMBO Rep. *13*, 197–203.

Moon, R.W., Taylor, C.J., Bex, C., Schepers, R., Goulding, D., Janse, C.J., Waters, A.P., Baker, D.A., and Billker, O. (2009). A cyclic GMP signalling module that regulates gliding motility in a malaria parasite. PLoS Pathog. *5*, e1000599.

Moorhead, G.B., Trinkle-Mulcahy, L., and Ulke-Lemée, A. (2007). Emerging roles of nuclear protein phosphatases. Nat. Rev. Mol. Cell Biol. *8*, 234–244.

Moorhead, G.B., De Wever, V., Templeton, G., and Kerk, D. (2009). Evolution of protein phosphatases in plants and animals. Biochem. J. *417*, 401–409.

Painter, H.J., Campbell, T.L., and Llinás, M. (2011). The Apicomplexan AP2 family: integral factors regulating Plasmodium development. Mol. Biochem. Parasitol. *176*, 1–7.

Patzewitz, E.M., Guttery, D.S., Poulin, B., Ramakrishnan, C., Ferguson, D.J., Wall, R.J., Brady, D., Holder, A.A., Szoor, B., and Tewari, R. (2013). An ancient protein phosphatase, SHLP1, is critical to microneme development in Plasmodium ookinetes and parasite transmission. Cell Rep. *3*, 622–629.

Pendyala, P.R., Ayong, L., Eatrides, J., Schreiber, M., Pham, C., Chakrabarti, R., Fidock, D.A., Allen, C.M., and Chakrabarti, D. (2008). Characterization of a PRL protein tyrosine phosphatase from Plasmodium falciparum. Mol. Biochem. Parasitol. *158*, 1–10.

Reece, S.E., Duncan, A.B., West, S.A., and Read, A.F. (2003). Sex ratios in the rodent malaria parasite, Plasmodium chabaudi. Parasitology *127*, 419–425.

Reece, S.E., Drew, D.R., and Gardner, A. (2008). Sex ratio adjustment and kin discrimination in malaria parasites. Nature *453*, 609–614.

Reininger, L., Billker, O., Tewari, R., Mukhopadhyay, A., Fennell, C., Dorin-Semblat, D., Doerig, C., Goldring, D., Harmse, L., Ranford-Cartwright, L., et al. (2005). A NIMA-related protein kinase is essential for completion of the sexual cycle of malaria parasites. J. Biol. Chem. *280*, 31957–31964.

Reininger, L., Tewari, R., Fennell, C., Holland, Z., Goldring, D., Ranford-Cartwright, L., Billker, O., and Doerig, C. (2009). An essential role for the Plasmodium Nek-2 Nima-related protein kinase in the sexual development of malaria parasites. J. Biol. Chem. *284*, 20858–20868.

Robert, V., Read, A.F., Essong, J., Tchuinkam, T., Mulder, B., Verhave, J.P., and Carnevale, P. (1996). Effect of gametocyte sex ratio on infectivity of Plasmodium falciparum to Anopheles gambiae. Trans. R. Soc. Trop. Med. Hyg. *90*, 621–624.

Sakumoto, N., Yamashita, H., Mukai, Y., Kaneko, Y., and Harashima, S. (2001). Dual-specificity protein phosphatase Yvh1p, which is required for vegetative growth and sporulation, interacts with yeast pescadillo homolog in Saccharomyces cerevisiae. Biochem. Biophys. Res. Commun. *289*, 608–615.

Sebastian, S., Brochet, M., Collins, M.O., Schwach, F., Jones, M.L., Goulding, D., Rayner, J.C., Choudhary, J.S., and Billker, O. (2012). A Plasmodium calcium-dependent protein kinase controls zygote development and transmission by translationally activating repressed mRNAs. Cell Host Microbe *12*, 9–19.

Siden-Kiamos, I., Ecker, A., Nybäck, S., Louis, C., Sinden, R.E., and Billker, O. (2006). Plasmodium berghei calcium-dependent protein kinase 3 is required for ookinete gliding motility and mosquito midgut invasion. Mol. Microbiol. *60*, 1355–1363.

Solyakov, L., Halbert, J., Alam, M.M., Semblat, J.P., Dorin-Semblat, D., Reininger, L., Bottrill, A.R., Mistry, S., Abdi, A., Fennell, C., et al. (2011). Global kinomic and phospho-proteomic analyses of the human malaria parasite Plasmodium falciparum. Nat. Commun. *2*, 565.

Srinivasan, P., Fujioka, H., and Jacobs-Lorena, M. (2008). PbCap380, a novel oocyst capsule protein, is essential for malaria parasite survival in the mosquito. Cell. Microbiol. *10*, 1304–1312.

Tewari, R., Dorin, D., Moon, R., Doerig, C., and Billker, O. (2005). An atypical mitogen-activated protein kinase controls cytokinesis and flagellar motility during male gamete formation in a malaria parasite. Mol. Microbiol. *58*, 1253–1263.

Tewari, R., Straschil, U., Bateman, A., Böhme, U., Cherevach, I., Gong, P., Pain, A., and Billker, O. (2010). The systematic functional analysis of *Plasmodium* protein kinases identifies essential regulators of mosquito transmission. Cell Host Microbe *8*, 377–387.

Trapnell, C., Pachter, L., and Salzberg, S.L. (2009). TopHat: discovering splice junctions with RNA-Seq. Bioinformatics *25*, 1105–1111.

Trapnell, C., Hendrickson, D.G., Sauvageau, M., Goff, L., Rinn, J.L., and Pachter, L. (2013). Differential analysis of gene regulation at transcript resolution with RNA-seq. Nat. Biotechnol. *31*, 46–53.

Treeck, M., Sanders, J.L., Elias, J.E., and Boothroyd, J.C. (2011). The phosphoproteomes of Plasmodium falciparum and Toxoplasma gondii reveal unusual adaptations within and beyond the parasites' boundaries. Cell Host Microbe *10*, 410–419.

Tremp, A.Z., Khater, E.I., and Dessens, J.T. (2008). IMC1b is a putative membrane skeleton protein involved in cell shape, mechanical strength, motility, and infectivity of malaria ookinetes. J. Biol. Chem. *283*, 27604–27611.

Virshup, D.M., and Shenolikar, S. (2009). From promiscuity to precision: protein phosphatases get a makeover. Mol. Cell *33*, 537–545.

Ward, P., Equinet, L., Packer, J., and Doerig, C. (2004). Protein kinases of the human malaria parasite Plasmodium falciparum: the kinome of a divergent eukaryote. BMC Genomics *5*, 79.

WHO (2013). World Malaria Report, 2013. (Geneva: World Health Organization).

Wickstead, B., Gull, K., and Richards, T.A. (2010). Patterns of kinesin evolution reveal a complex ancestral eukaryote with a multifunctional cytoskeleton. BMC Evol. Biol. *10*, 110.

Wilkes, J.M., and Doerig, C. (2008). The protein-phosphatome of the human malaria parasite Plasmodium falciparum. BMC Genomics *9*, 412.

Wright, M.H., Clough, B., Rackham, M.D., Rangachari, K., Brannigan, J.A., Grainger, M., Moss, D.K., Bottrill, A.R., Heal, W.P., Broncel, M., et al. (2014). Validation of N-myristoyltransferase as an antimalarial drug target using an integrated chemical biology approach. Nat. Chem. *6*, 112–121.

Yeo, M., Lin, P.S., Dahmus, M.E., and Gill, G.N. (2003). A novel RNA polymerase II C-terminal domain phosphatase that preferentially dephosphorylates serine 5. J. Biol. Chem. *278*, 26078–26085.

Yuda, M., Iwanaga, S., Shigenobu, S., Mair, G.R., Janse, C.J., Waters, A.P., Kato, T., and Kaneko, I. (2009). Identification of a transcription factor in the mosquito-invasive stage of malaria parasites. Mol. Microbiol. *71*, 1402–1414.

Zhang, M., Yogesha, S.D., Mayfield, J.E., Gill, G.N., and Zhang, Y. (2013). Viewing serine/threonine protein phosphatases through the eyes of drug designers. FEBS J. *280*, 4739–4760.

Cell Host & Microbe, Volume *16*

Supplemental Information

Genome-wide Functional Analysis of *Plasmodium* Protein Phosphatases Reveals Key Regulators of Parasite Development and Differentiation

David S. Guttery, Benoit Poulin, Abhinay Ramaprasad, Richard J. Wall, David J.P. Ferguson, Declan Brady, Eva-Maria Patzewitz, Sarah Whipple, Ursula Straschil, Megan H. Wright, Alyaa M.A.H. Mohamed, Anand Radhakrishnan, Stefan T. Arold, Edward W. Tate, Anthony A. Holder, Bill Wickstead, Arnab Pain, and Rita Tewari

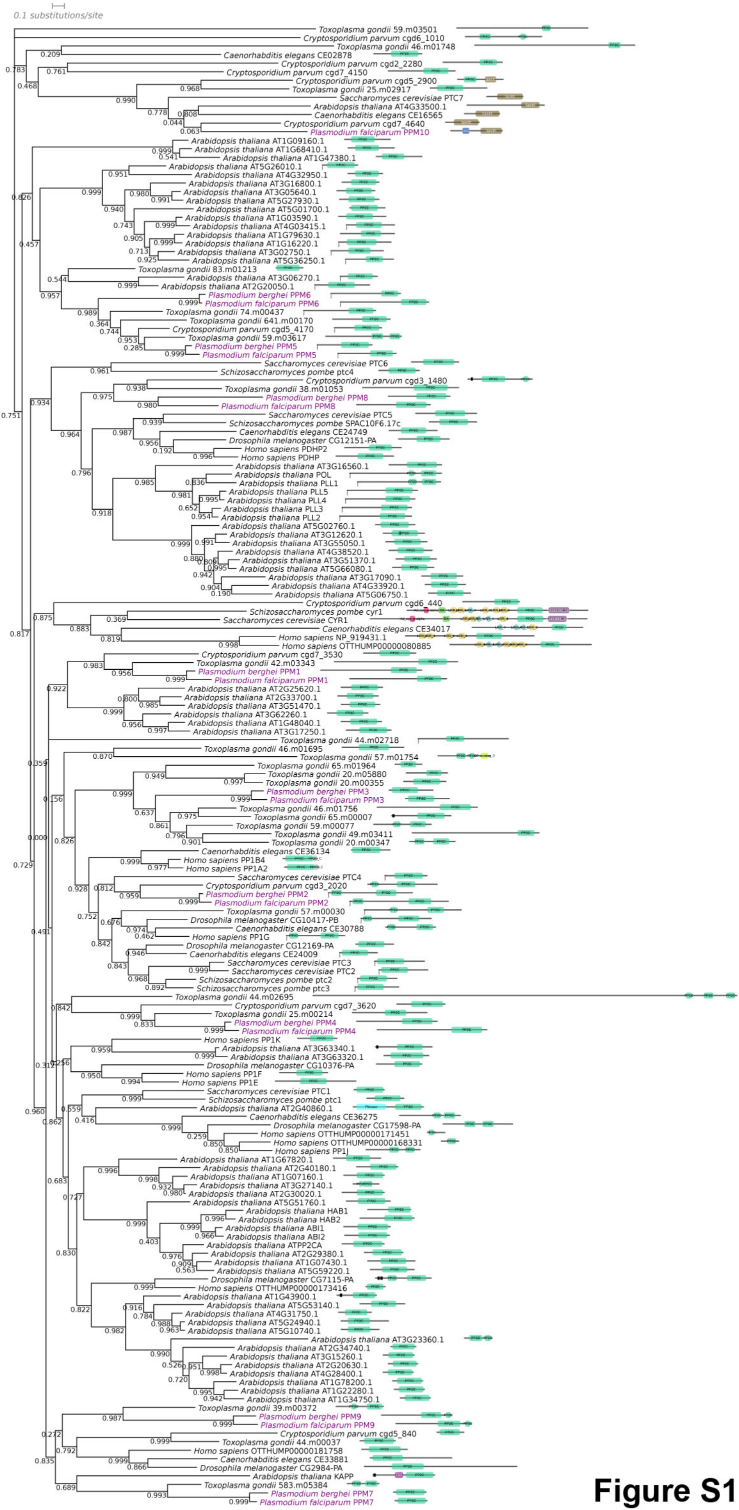
0.1 substitutions/site
Toxoplasma gondii 59.m03501
Cryptosporidium parvum cgd6_1010
Toxoplasma gondii 46.m01748
Caenorhabditis elegans CE02878
Cryptosporidium parvum cgd2_2280
Cryptosporidium parvum cgd7_4150
Cryptosporidium parvum cgd5_2900
Toxoplasma gondii 25.m02917
Saccharomyces cerevisiae PTC7
Arabidopsis thaliana AT4G33500.1
Caenorhabditis elegans CE16565
Cryptosporidium parvum cgd7_4640
Plasmodium falciparum PPM10
Arabidopsis thaliana AT1G09160.1
Arabidopsis thaliana AT1G68410.1
Arabidopsis thaliana AT1G47380.1
Arabidopsis thaliana AT5G26010.1
Arabidopsis thaliana AT4G32950.1
Arabidopsis thaliana AT3G16800.1
Arabidopsis thaliana AT3G05640.1
Arabidopsis thaliana AT5G27930.1
Arabidopsis thaliana AT5G01700.1
Arabidopsis thaliana AT1G03590.1
Arabidopsis thaliana AT4G03415.1
Arabidopsis thaliana AT1G79630.1
Arabidopsis thaliana AT1G16220.1
Arabidopsis thaliana AT3G02750.1
Arabidopsis thaliana AT5G36250.1
Toxoplasma gondii 83.m01213
Arabidopsis thaliana AT3G06270.1
Arabidopsis thaliana AT2G20050.1
Plasmodium berghei PPM6
Plasmodium falciparum PPM6
Toxoplasma gondii 74.m00437
Toxoplasma gondii 641.m00170
Cryptosporidium parvum cgd5_4170
Toxoplasma gondii 59.m03617
Plasmodium berghei PPM5
Plasmodium falciparum PPM5
Saccharomyces cerevisiae PTC6
Schizosaccharomyces pombe ptc4
Cryptosporidium parvum cgd3_1480
Toxoplasma gondii 38.m01053
Plasmodium berghei PPM8
Plasmodium falciparum PPM8
Saccharomyces cerevisiae PTC5
Schizosaccharomyces pombe SPAC10F6.17c
Caenorhabditis elegans CE24749
Drosophila melanogaster CG12151-PA
Homo sapiens PDHP2
Homo sapiens PDHP
Arabidopsis thaliana AT3G16560.1
Arabidopsis thaliana POL
Arabidopsis thaliana PLL1
Arabidopsis thaliana PLL5
Arabidopsis thaliana PLL4
Arabidopsis thaliana PLL3
Arabidopsis thaliana PLL2
Arabidopsis thaliana AT5G02760.1
Arabidopsis thaliana AT3G12620.1
Arabidopsis thaliana AT3G55050.1
Arabidopsis thaliana AT4G38520.1
Arabidopsis thaliana AT3G51370.1
Arabidopsis thaliana AT5G66080.1
Arabidopsis thaliana AT3G17090.1
Arabidopsis thaliana AT4G33920.1
Arabidopsis thaliana AT5G06750.1
Cryptosporidium parvum cgd6_440
Schizosaccharomyces pombe cyr1
Saccharomyces cerevisiae CYR1
Caenorhabditis elegans CE34017
Homo sapiens NP_919431.1
Homo sapiens OTTHUMP00000080885
Cryptosporidium parvum cgd7_3530
Toxoplasma gondii 42.m03343
Plasmodium berghei PPM1
Plasmodium falciparum PPM1
Arabidopsis thaliana AT2G25620.1
Arabidopsis thaliana AT2G33700.1
Arabidopsis thaliana AT3G51470.1
Arabidopsis thaliana AT3G62260.1
Arabidopsis thaliana AT1G48040.1
Arabidopsis thaliana AT3G17250.1
Toxoplasma gondii 44.m02718
Toxoplasma gondii 46.m01695
Toxoplasma gondii 57.m01754
Toxoplasma gondii 65.m01964
Toxoplasma gondii 20.m05880
Toxoplasma gondii 20.m00355
Plasmodium berghei PPM3
Plasmodium falciparum PPM3
Toxoplasma gondii 46.m01756
Toxoplasma gondii 65.m00007
Toxoplasma gondii 59.m00077
Toxoplasma gondii 49.m03411
Toxoplasma gondii 20.m00347
Caenorhabditis elegans CE36134
Homo sapiens PP1B4
Homo sapiens PP1A2
Saccharomyces cerevisiae PTC4
Cryptosporidium parvum cgd3_2020
Plasmodium berghei PPM2
Plasmodium falciparum PPM2
Toxoplasma gondii 57.m00030
Drosophila melanogaster CG10417-PB
Caenorhabditis elegans CE30788
Homo sapiens PP1G
Drosophila melanogaster CG12169-PA
Caenorhabditis elegans CE24009
Saccharomyces cerevisiae PTC3
Saccharomyces cerevisiae PTC2
Schizosaccharomyces pombe ptc2
Schizosaccharomyces pombe ptc3
Toxoplasma gondii 44.m02695
Cryptosporidium parvum cgd7_3620
Toxoplasma gondii 25.m00214
Plasmodium berghei PPM4
Plasmodium falciparum PPM4
Homo sapiens PP1K
Arabidopsis thaliana AT3G63340.1
Arabidopsis thaliana AT3G63320.1
Drosophila melanogaster CG10376-PA
Homo sapiens PP1F
Homo sapiens PP1E
Saccharomyces cerevisiae PTC1
Schizosaccharomyces pombe ptc1
Arabidopsis thaliana AT2G40860.1
Caenorhabditis elegans CE36275
Drosophila melanogaster CG17598-PA
Homo sapiens OTTHUMP00000171451
Homo sapiens OTTHUMP00000168331
Homo sapiens PP1J
Arabidopsis thaliana AT1G67820.1
Arabidopsis thaliana AT2G40180.1
Arabidopsis thaliana AT1G07160.1
Arabidopsis thaliana AT3G27140.1
Arabidopsis thaliana AT2G30020.1
Arabidopsis thaliana AT5G51760.1
Arabidopsis thaliana HAB1
Arabidopsis thaliana HAB2
Arabidopsis thaliana ABI1
Arabidopsis thaliana ABI2
Arabidopsis thaliana ATPP2CA
Arabidopsis thaliana AT2G29380.1
Arabidopsis thaliana AT1G07430.1
Arabidopsis thaliana AT5G59220.1
Drosophila melanogaster CG7115-PA
Homo sapiens OTTHUMP00000173416
Arabidopsis thaliana AT1G43900.1
Arabidopsis thaliana AT5G53140.1
Arabidopsis thaliana AT4G31750.1
Arabidopsis thaliana AT5G24940.1
Arabidopsis thaliana AT5G10740.1
Arabidopsis thaliana AT3G23360.1
Arabidopsis thaliana AT2G34740.1
Arabidopsis thaliana AT3G15260.1
Arabidopsis thaliana AT2G20630.1
Arabidopsis thaliana AT4G28400.1
Arabidopsis thaliana AT1G78200.1
Arabidopsis thaliana AT1G22280.1
Arabidopsis thaliana AT1G34750.1
Toxoplasma gondii 39.m00372
Plasmodium berghei PPM9
Plasmodium falciparum PPM9
Cryptosporidium parvum cgd5_840
Toxoplasma gondii 44.m00037
Homo sapiens OTTHUMP00000181758
Caenorhabditis elegans CE33881
Drosophila melanogaster CG2984-PA
Arabidopsis thaliana KAPP
Toxoplasma gondii 583.m05384
Plasmodium berghei PPM7
Plasmodium falciparum PPM7

Figure S1

Figure S1, related to Figure 1: Phylogenetic analysis of the PPMs

A maximum likelihood tree inferred from 288 alignable residues is shown with topology support from the minimum of parametric χ^2-based or Shimodaira-Hasegawa-like tests. All PPM phosphatases identified in the predicted proteomes of *Plasmodium berghei*, *Plasmodium falciparum* and also *Arabidopsis thaliana*, *Caenorhabditis elegans*, *Cryptosporidium parvum*, *Drosophila melanogaster*, *Homo sapiens*, *Saccharomyces cerevisiae*, *Schizosaccharomyces pombe* and *Toxoplasma gondii*, were trimmed to the conserved phosphatase region, aligned and highly similar sequences were removed. Tree and topology support were inferred using PhyML-aLRT (http://www.atgc-montpellier.fr/phyml/alrt/) with the WAG substitution matrix and a gamma-distributed variation in substitution rate approximated to 4 discrete categories (shape parameter estimated from the data). Protein domain architectures were predicted as described in Supplemental Experimental Procedures.

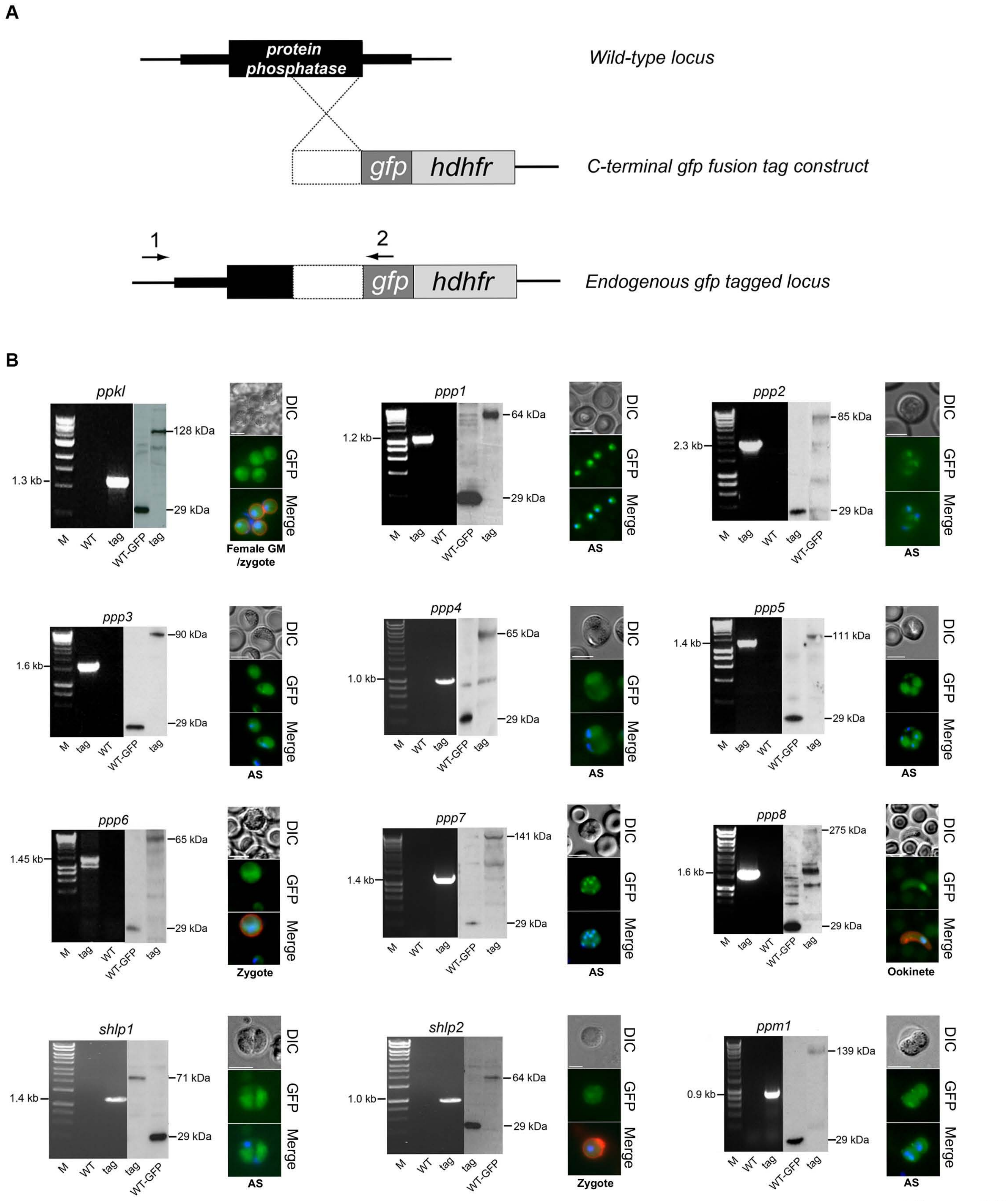
A
protein phosphatase
Wild-type locus
gfp
hdhfr
C-terminal gfp fusion tag construct
1
2
Endogenous gfp tagged locus
B
ppkl
ppp1
ppp2
ppp3
ppp4
ppp5
ppp6
ppp7
ppp8
shlp1
shlp2
ppm1
M
WT
tag
WT-GFP
DIC
GFP
Merge
Female GM /zygote
AS
Zygote
Ookinete
1.3 kb
128 kDa
29 kDa
1.2 kb
64 kDa
2.3 kb
85 kDa
1.6 kb
90 kDa
1.0 kb
65 kDa
1.4 kb
111 kDa
1.45 kb
141 kDa
275 kDa
71 kDa
0.9 kb
139 kDa

Figure S2

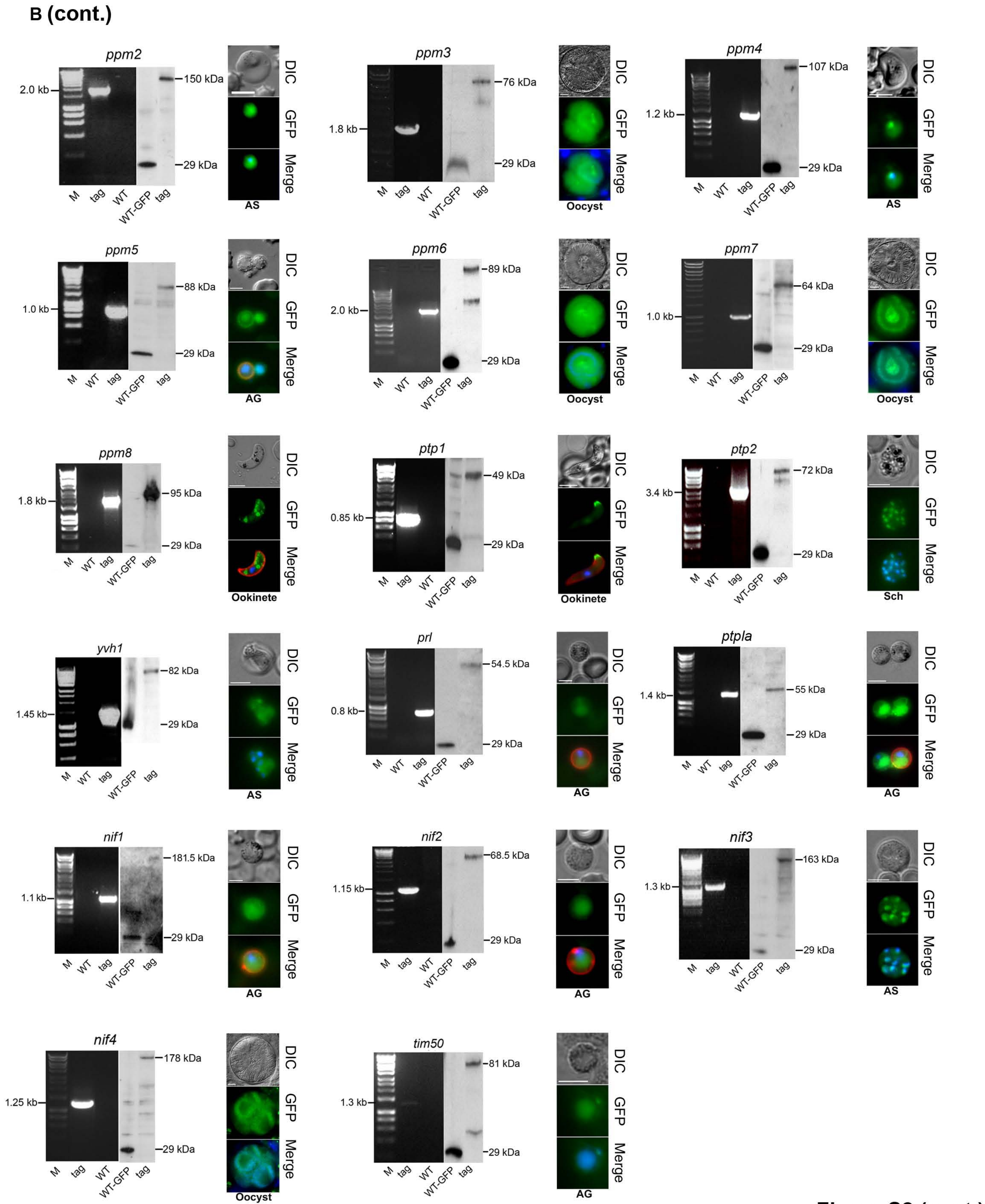

Figure S2 (cont.)

Figure S2, related to Figure 2: *gfp* tagging of the PPs and representative expression

(A) Schematic representation for 3'-tagging of each endogenous *P. berghei* PP gene with *gfp* via single homologous recombination. Primers 1+2 used for diagnostic PCR are indicated. (B) For each PP are shown: (left) diagnostic integration PCR showing band at the expected size, confirming successful integration of the tagging construct; (middle) Western blot analysis using an anti-GFP antibody against control GFP (WT-GFP) and transgenic (tag) parasite protein showing bands of 29 kDa for GFP and of the expected size for the corresponding PP-GFP; (right) images showing expression of the GFP tagged PP in one of the representative stages of the life cycle: (asexual blood stages (AS), schizonts (Sch), activated gametocytes (AG), ookinete (Ook) and oocyst). A Cy3-conjugated antibody recognising P28 on the surface of activated female gametocytes, zygotes, and ookinetes was used for the sexual stages, and the cells were displayed by differential interference contrast (DIC). Merge is the composite image of Hoechst dye to detect the nuclei, GFP, and P28 for the sexual stages. Bar = 5 µm.

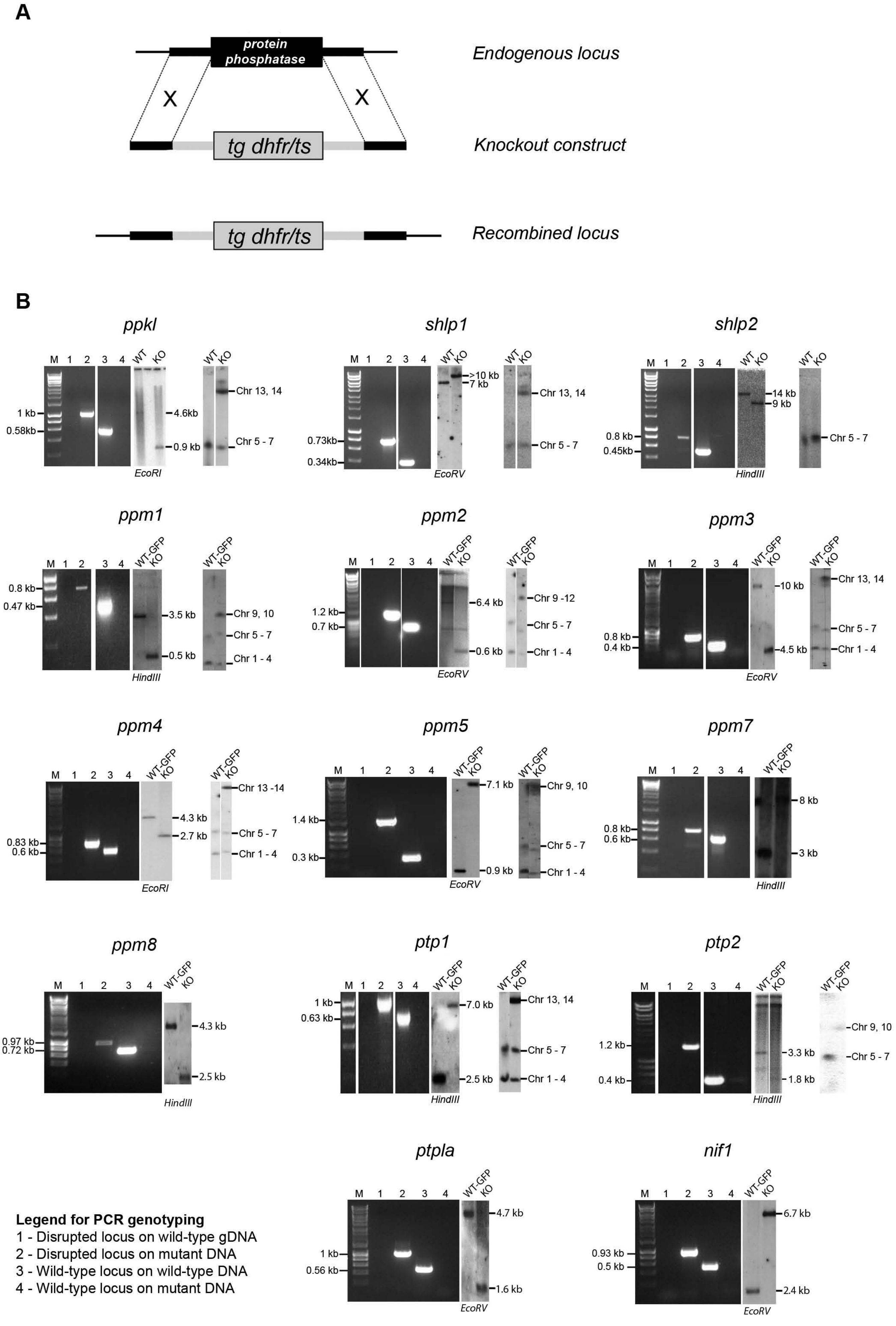

A
protein phosphatase
Endogenous locus
Knockout construct
tg dhfr/ts
Recombined locus
tg dhfr/ts
B
ppkl
shlp1
shlp2
ppm1
ppm2
ppm3
ppm4
ppm5
ppm7
ppm8
ptp1
ptp2
ptpla
nif1
M 1 2 3 4
WT KO
WT-GFP KO
EcoRI
EcoRV
HindIII
Chr 13, 14
Chr 5 - 7
Chr 9, 10
Chr 9 -12
Chr 1 - 4
Chr 13 -14
1 kb
0.58kb
4.6kb
0.9 kb
0.73kb
0.34kb
>10 kb
7 kb
0.8 kb
0.45kb
14 kb
9 kb
0.47 kb
3.5 kb
0.5 kb
1.2 kb
0.7 kb
6.4 kb
0.6 kb
0.4 kb
10 kb
4.5 kb
0.83 kb
4.3 kb
2.7 kb
1.4 kb
0.3 kb
7.1 kb
8 kb
3 kb
0.97 kb
0.72 kb
2.5 kb
0.63 kb
7.0 kb
3.3 kb
1.8 kb
4.7 kb
0.56 kb
1.6 kb
0.93 kb
6.7 kb
2.4 kb
Legend for PCR genotyping
1 - Disrupted locus on wild-type gDNA
2 - Disrupted locus on mutant DNA
3 - Wild-type locus on wild-type DNA
4 - Wild-type locus on mutant DNA

Figure S3

Figure S3, related to Figure 2: Generation of PP mutants and genotypic analyses

(A) Schematic representation of the endogenous protein phosphatase gene locus, the knockout construct and the recombined protein phosphatase gene locus following double cross-over recombination. The knockout construct contains a *Toxoplasma gondii* dihydrofolate reductase/thymidylate synthase (*tgdhfr/ts*) cassette with a *Pbdhfr* 3'UTR for selection of transgenic parasites with pyrimethamine. (B) Diagnostic PCR, Southern blot and pulse-field gel electrophoresis for each of the phosphatase knock out lines generated. (left) Integration PCR (lanes 1 and 2) showing the presence of a band at the indicated size using integration specific primers on gDNA of the deletion mutant (lane 2) indicating correct integration of the knockout construct, and knock out PCR (lanes 3 and 4) showing the absence of the wild-type specific band amplified by knock out specific primers demonstrating the loss of the wild-type locus in the deletion mutant (lane 4). WT gDNA was used as control. (middle) Southern blot analysis of GFP parasites gDNA (WT-GFP) and phosphatase mutant (KO) gDNA showing the bands recognised by the probe for the endogenous locus and for the recombined locus. The enzyme used for the digestion is indicated at the bottom of the blot. (right) Pulse-field gel electrophoresis (PFGE) analysis of WT-GFP (WT-GFP) and phosphatase mutant (KO) gDNA. Chromosomes on which the probe hybridises to the endogenous *dhfr* locus and the disrupted locus are indicated. Only one representative clone for each of the phosphatase deletion mutants (out of at least 2 independent clones obtained for each phosphatase deletion mutant) is presented in the phenotypic analyses.

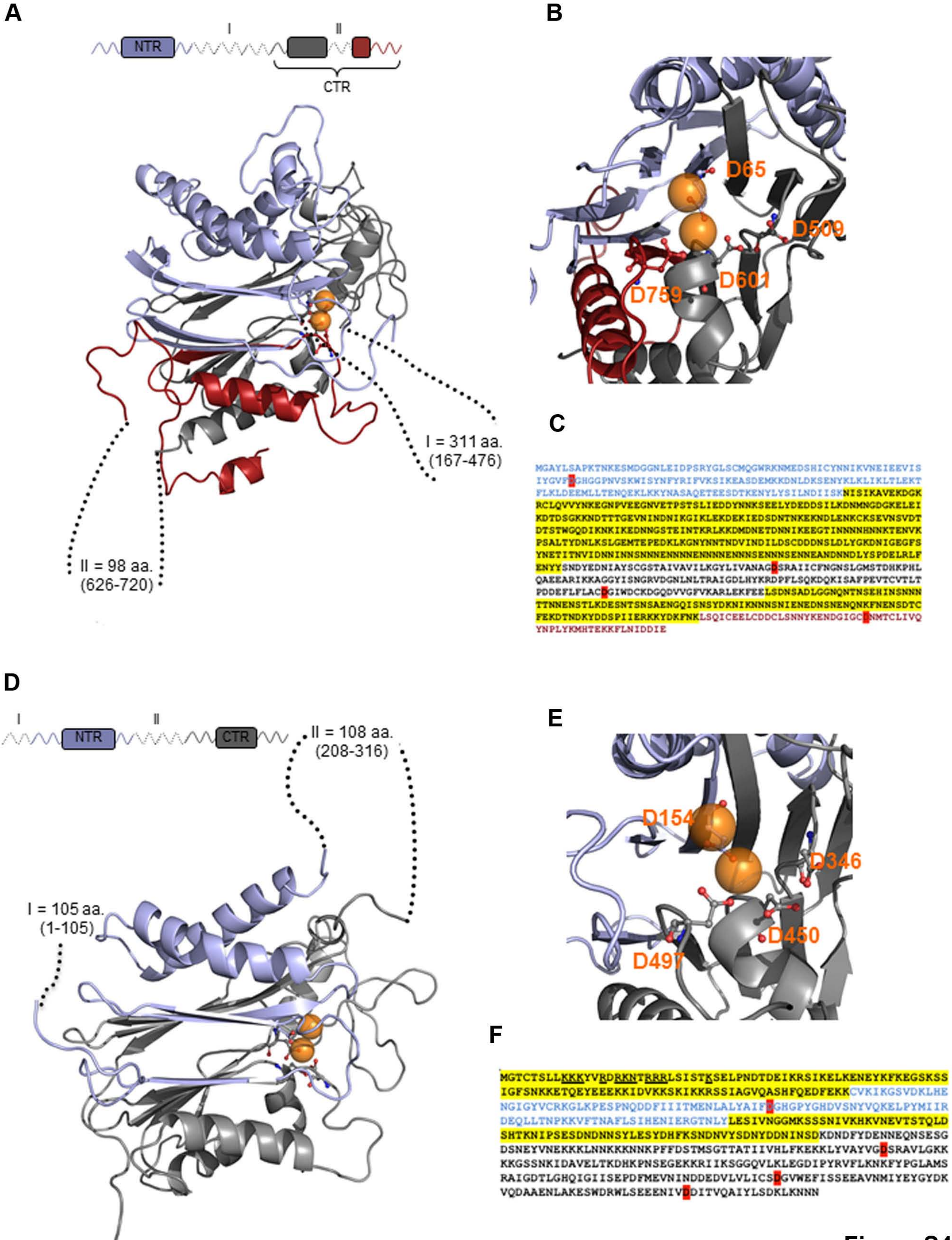
A
NTR
I
II
CTR
I = 311 aa.
(167-476)
II = 98 aa.
(626-720)
B
D65
D509
D601
D759
C
MGAYLSAPKTNKESMDGGNLEIDPSRYGLSCMQGWRKNMEDSHICYNNIKVNEIEEVIS
IYGVFPDGHGGPNVSKWISYNFYRIFVKSIKEASDEMKKDNLDKSENYKLKLIKLTLEKT
FLKLDEEMLLTENQEKLKKYNASAQETEESDTKENYLYSILNDIISKNISIKAVEKDGK
RCLQVVYNKEGNPVEEGNVETPSTSLIEDDYNNKSEELYDEDDSILKDNMNGDGKELEI
KDTDSGKKNDTTTGEVNINDNIKGIKLEKDEKIEDSDNTNKEKNDLENKCKSEVNSVDT
DTSTWGQDIKNKIKEDNNGSTEINTKRLKKDMDNETDNNIKEEGTINNNNHNNKTENVK
PSALTYDNLKSLGEMTEPEDKLKGNYNNTNDVINDILDSCDDDNSLDLYGKDNIGEGFS
YNETITNVIDNNINNSNNNENNNNENNNNENNNSENNNSENNEANDNNDLYSPDELRLF
ENYYSNDYEDNIAYSCGSTAIVAVILKGYLIVANAGDSRAIICFNGNSLGMSTDHKPHL
QAEEARIKKAGGYISNGRVDGNLNLTRAIGDLHYKRDPFLSQKDQKISAFPEVTCVTLT
PDDEFLFLACDGIWDCKDGQDVVGFVKARLEKFEELSDNSADLGGNQNTNSEHINSNNN
TTNNENSTLKDESNTSNSAENGQISNSYDKNIKNNNSNIENEDNSNENQNKFNENSDTC
FEKDTNDKYDDSPIIERKKYDKFNKLSQICEELCDDCLSNNYKENDGIGCDNMTCLIVQ
YNPLYKMHTEKKFLNIDDIE
D
I
NTR
II
CTR
II = 108 aa.
(208-316)
I = 105 aa.
(1-105)
E
D154
D346
D450
D497
F
MGTCTSLLKKKYVRDRKNTRRRLSISTKSELPNDTDEIKRSIKELKENEYKFKEGSKSS
IGFSNKKETQEYEEEKKIDVKKSKIKKRSSIAGVQASHFQEDFEKKCVKIKGSVDKLHE
NGIGYVCRKGLKPESPNQDDFIIITMENLALYAIFDGHGPYGHDVSNYVQKELPYMIIR
DEQLLTNPKKVFTNAFLSIHENIERGTNLYLESIVNGGMKSSSNIVKHKVNEVTSTQLD
SHTKNIPSESDNDNNSYLESYDHFKSNDNVYSDNYDDNINSDKDNDFYDENNEQNSESG
DSNEYVNEKKKLNNKKKNNKPFFDSTMSGTTATIIVHLFKEKKLYVAYVGDSRAVLGKK
KKGSSNKIDAVELTKDHKPNSEGEKKRIIKSGGQVLKLEGDIPYRVFLKNKFYPGLAMS
RAIGDTLGHQIGIISEPDFMEVNINDDEDVLVLICSDGVWEFISSEEAVNMIYEYGYDK
VQDAAENLAKESWDRWLSEEENIVDDITVQAIYLSDKLKNNN

Figure S4

Figure S4, related to Figure 3: Structural analysis of the PPMs

Computational homology models for (A-C) PPM2 and (D-F) PPM5. (A) PPM2 is shown in a schematic representation (top) colour-matched to the molecular model (below). The extensive loop regions are indicated by dotted lines, with the loop length shown. Metal ions are shown as orange spheres. (B) Zoom into the active site, showing the aspartic acid residues that coordinate the metal ions. (C) The active site is composed of residues far apart in sequence, belonging to structural modules intercepted by long unstructured loops. The N-rich loops of PPM2 are highlighted in yellow background, the N-terminal phosphatase domain (NTR) in blue text and the C-terminal domain (CTR) is shown in black and red text. Loop I separates the NTR from the CTR and loop II bisects the CTR. The metal coordinating residues are highlighted in red background. (D-F) show PPM5 using the same representations.

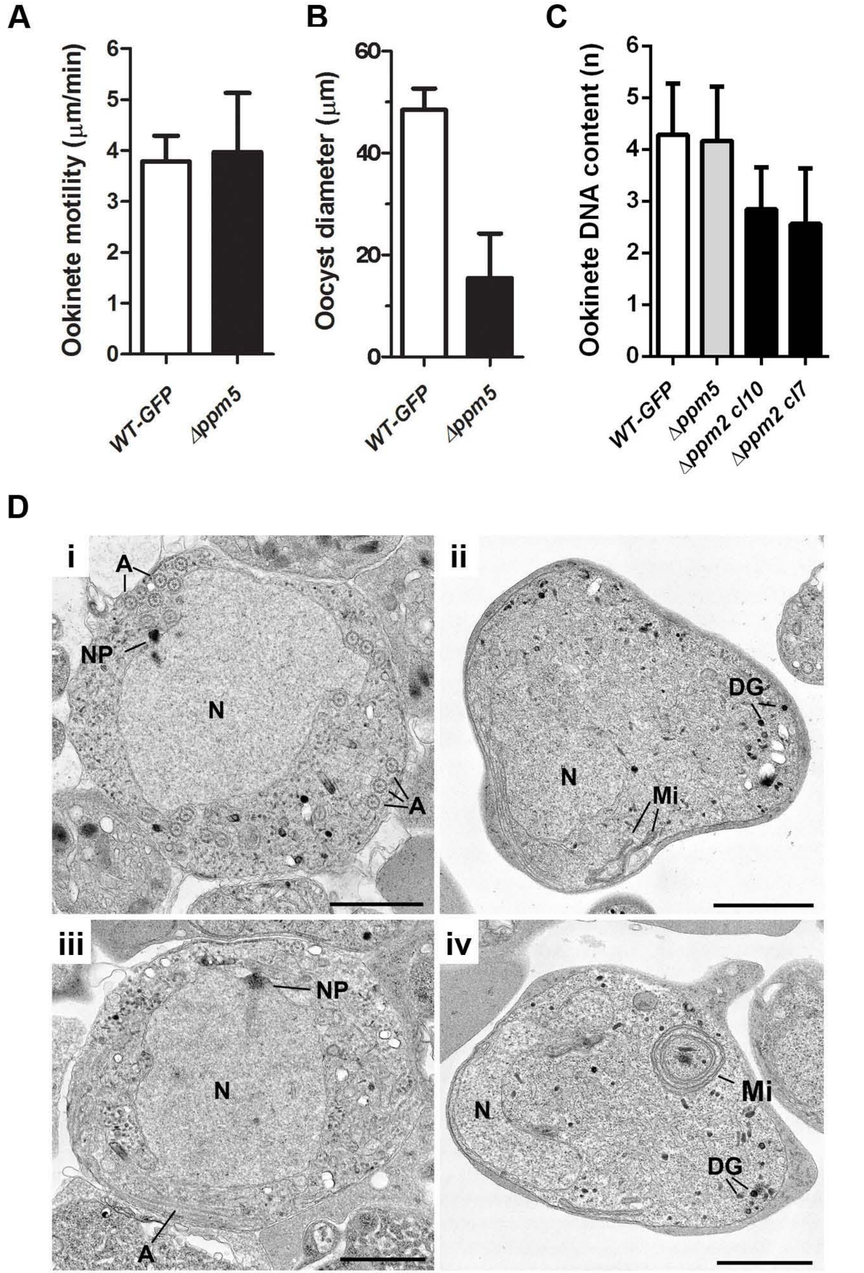
A
B
C
Ookinete motility (μm/min)
6
5
4
3
2
1
0
WT-GFP
Δppm5
Oocyst diameter (μm)
60
40
20
0
WT-GFP
Δppm5
Ookinete DNA content (n)
6
5
4
3
2
1
0
WT-GFP
Δppm5
Δppm2 cl10
Δppm2 cl7
D
i
A
A
NP
N
A
ii
DG
N
Mi
iii
NP
N
A
iv
Mi
N
DG

Figure S5

Figure S5, related to Figure 4: Phenotypic analysis of Δ*ppm5* and ultrastructure of Δ*ppm2* activated gametocytes

(A) Motility of Δ*ppm5* ookinetes. Velocity (μm/min) of individual WT-GFP or Δ*ppm5* ookinetes from 24 hr cultures was measured over 10 min (Error bar ±SD; n = 10). (B) Size of Δ*ppm5* oocysts. Diameter of WT-GFP or Δ*ppm5* oocysts day 14 post-infection was measured using AxioVision software (error bar ±SD; n = 45). (C) Fluorometric DNA content (n) analysis of WT-GFP and Δ*ppm5* ookinetes, and two independent clones of Δ*ppm2* (cl7 and cl10) after Hoechst nuclear staining. Nuclear fluorescence intensity of WT-GFP or mutant parasites from 24 hr cultures was measured using ImageJ software. Values are expressed relative to the average fluorescence intensity of haploid ring-stage parasites from the same slide and corrected for background fluorescence (Error bar ±SD; n = 15 for WT-GFP and Δ*ppm5* ookinetes, and n = 38 for each clone of Δ*ppm2*) (D) Electron micrographs of the activated gametocytes of wild type (i and ii) and Δ*ppm2* (iii and iv) parasites. Bar = 1 μm. (i) Section through a developing wild type microgametocyte showing the central nucleus (N) with a nuclear pole (NP) and a number of axonemes (A) within the cytoplasm. (ii) Section through a wild-type macrogametocyte showing the more peripherally located nucleus (N), with flattened mitochondria (Mi) and a few dense bodies (DG) in the cytoplasm. (iii) Section through a developing Δ*ppm2* microgametocyte showing the central nucleus (N) with a nuclear pole (NP) plus a number of axonemes (A) forming within the cytoplasm as seen in the wild type. (iv) Section through a Δ*ppm2* macrogametocyte showing the more peripherally located nucleus (N), mitochondrion (Mi) and dense bodies (DG) as seen in wild-type.

A

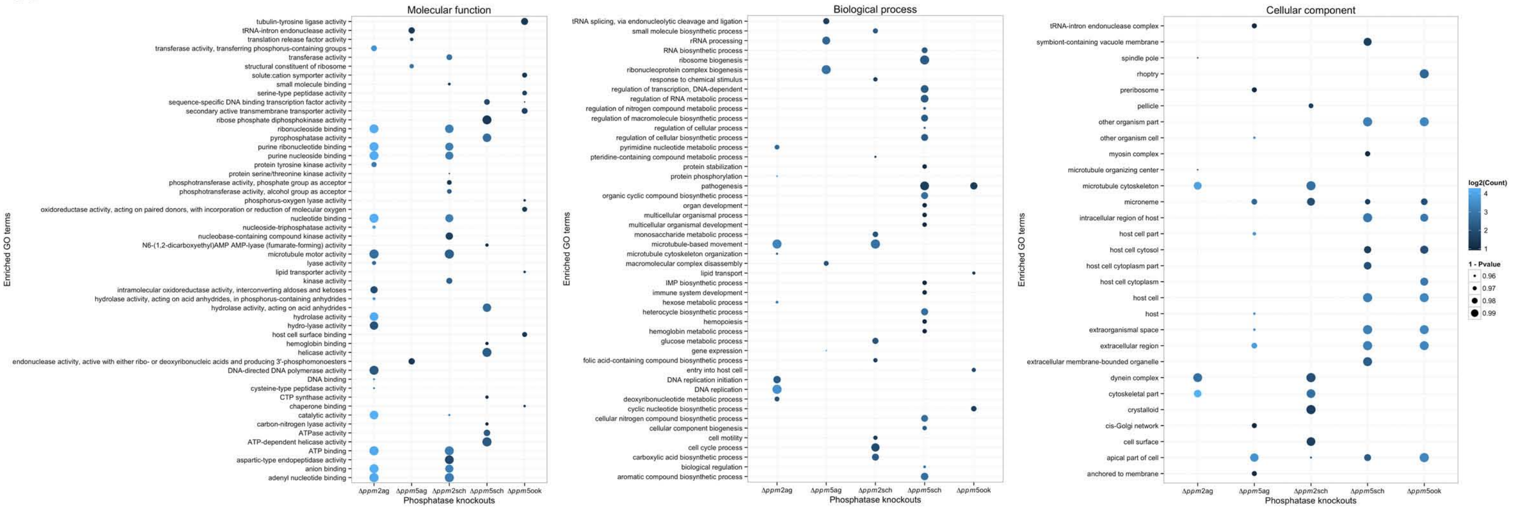

B

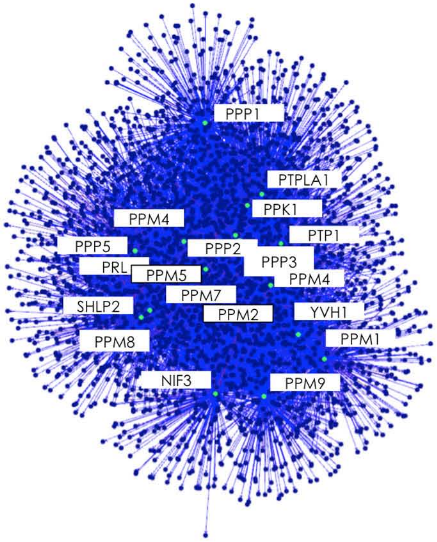

C

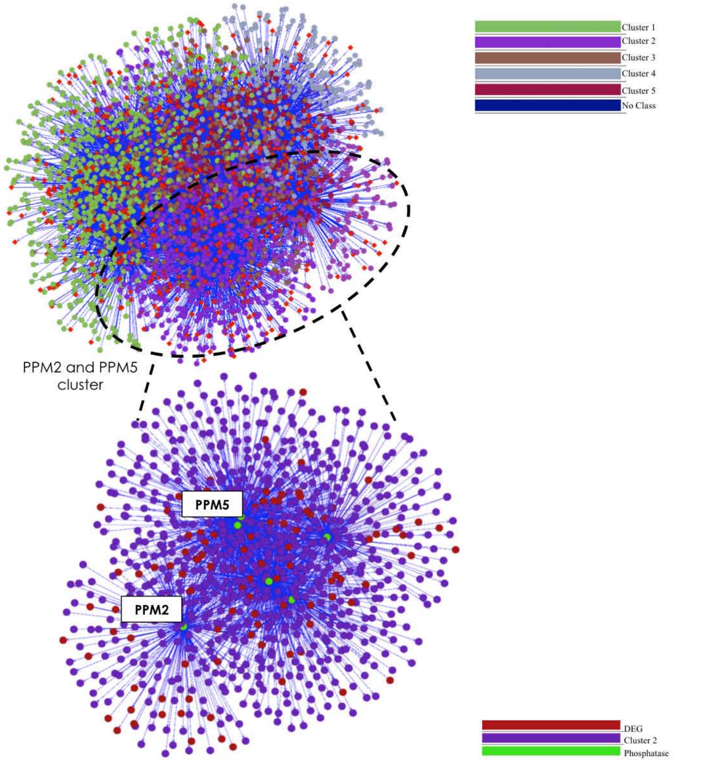

D

PBANKA_091020 (PPM2)				
network	number of interaction partners	number of differentially expressed genes	intersection size (%)	overlap p value
top 10%	312	1486	77 (5.1%)	0.06986
top 50%	1740	1486	454 (30.5%)	0.0002414

PBANKA_142720 (PPM5)				
network	number of interaction partners	number of differentially expressed genes	intersection size (%)	overlap p value
top 10%	391	563	29 (5.1%)	0.01904
top 50%	1734	563	160 (28.4%)	0.00225

Figure S6

Figure S6, related to Figure 5: Gene ontology analysis and phosphatase interaction subnetworks

(A) Gene ontology (GO) enrichment analysis of significantly differentially expressed genes in Δ*ppm2* and Δ*ppm5* in schizonts, activated gametocytes and ookinetes (only in Δ*ppm5*). GO terms obtained from the GeneDB database. Size of the bubble indicates the level of significance (1-*p* value) of the enriched GO term and colour density indicates the number of differentially expressed genes ($\log_2$ of gene count) associated with that GO term. (B) Interaction subnetwork of *P. berghei* phosphatome derived from growth perturbation transcriptome of *P. falciparum.* (C) top: 5 MCL (Markov-Cluster Algorithm http://micans.org/mcl/) clusters of the phosphatases subnetwork (refer to methods for details) bottom: zoomed view of cluster 2 where PPM2 and PPM5 co-cluster. Differentially expressed genes (coloured in red) in the Δ*ppm2 and* Δ*ppm5* PP mutants were significantly overlapping with PPM2 and PPM5 interaction partners with *p-values* 0.00024 and 0.0022, respectively. (D) Number of interaction partners for PPM2 and PPM5 in the top 10% and top 50% phosphatases subnetworks, and the significance of overlap with differentially expressed genes.

SUPPLEMENTAL TABLE LEGENDS

Table S1, related to Figure 2: Proposed protein phosphatase gene names, C-terminal GFP fusion of endogenous PPs and summary of deletion attempts

Total number of C-terminal GFP fusion transfection and gene knock-out attempts and how many led to successful tagging or successful deletion of the target as confirmed by genotypic analysis. We were unable to successfully tag PPM9. *P* value gives an estimate for the likelihood of error for "possibly essential" genes; it is calculated from the average technical failure rate (0.547 per attempt for redundant genes in this study) to the power of *n*, where *n* is the total number of deletion attempts for the gene.

Table S2, related to Figure 2: Phenotypic analysis of deletion mutants

Raw data from phenotyping analysis of 14 mutants at different development stages. All data are given as a percentage of wild-type controls studied in parallel. SD = standard deviation. *n* = number of replicate experiments.

Table S3, related to Figure 5: Significantly altered gene expression in Δ*ppm2* and Δ*ppm5* mutants compared to wild-type and putative interactions in phosphatase networks

Raw differential expression, $\log_2$ fold change values and putative interactions in phosphatise networks of significantly altered genes in Δ*ppm2* and Δ*ppm5* mutants. Gene names were obtained from GeneDB.

Table S4, related to Figure 5: Heatmap clusters and $\log_2$ ratios of gene expression

Differential expression ($\log_2$ fold change; Table S3) values used to produce the heatmaps in Figure 5C of protein phosphatases, protein kinases, RNA helicases, AP2 transcription factors, host invasion- and microneme- related proteins, microtubules/axonemes related dyneins and kinesins and enzymes involved in glycolysis (BIR proteins; not included in

Figure 5C), in Δ*ppm2* and Δ*ppm5* at schizont, activated gametocyte and ookinete life stages. Cells highlighted in green were upregulated and red were downregulated. Sch = schizonts; AG = activated gametocytes; Ook = ookinetes.

Table S5, related to Figure 2: Primers used for generation of C-terminal GFP fusion, gene deletion constructs and genotype analysis

Generic sequences for *Kpn*I and *Apa*I restriction sites; used for GFP fusion cloning purposes. ol492 sequence is given in (Guttery et al., 2012). Generic sequences for *Apa*I/*HindIII* and *EcoR*I and *Xba*I restriction sites; used for gene deletion cloning purposes. ol248 and ol539 sequences are given in (Tewari et al., 2010).

Table S6, related to Figure 3: Primers used for qRT-PCR

Sequences shown are written 5' – 3'.

SUPPLEMENTAL EXPERIMENTAL PROCEDURES

Ethics statement

All animal work has passed an ethical review process and was approved by the United Kingdom Home Office. Work was carried out in accordance with the United Kingdom 'Animals (Scientific Procedures) Act 1986' and in compliance with 'European Directive 86/609/EEC' for the protection of animals used for experimental purposes. The project licence number is 40/3344.

Animals

Six to eight week old female Tuck-Ordinary (TO) outbred mice (Harlan) were used for all experiments.

Bioinformatic analysis

PPs from a variety of families were identified in the predicted proteomes of *P. berghei*, *P. falciparum* and 44 other diverse eukaryotes for which complete genome sequence is available (see (Wickstead et al., 2010) for full list). HMMER3 (Eddy, 2009) was used to search these proteomes for matches to the PFam profiles: DSPc (PF00782.15), LMWPc (PF01451.16), Metallophos (PF00149.23), NIF (PF03031.13), PP2C (PF00481.16), PTPLA (PF04387.9) and Y-phosphatase domains (PF03162.8, PF13350.1 and PF00102.22), all with a threshold of e-value ≤ 10^{-5}. For classification, full-length proteins were clustered using a neighbour-joining approach based on all-versus-all BLASTp scores (Wickstead and Gull, 2007) and large-scale maximum-likelihood phylogenies were built from trimmed alignments of phosphatase domains (MAFFT6.24 (Katoh et al., 2002)) with statistical support for nodes from the approximate Likelihood Ratio Test (Anisimova et al., 2010) as implemented by PhyML3.0 (Guindon and Gascuel, 2003). Protein domain architectures were predicted from the models in Pfam26 with e-value ≤ 0.001. Possible myristoylation was predicted using *N*-Myristoyltransferase (NMT) Myristoylator (Bologna et al., 2004).

Generation and genotyping of transgenic parasites

For C-terminal tagging of each PP with GFP by single homologous recombination, a targeting vector was generated using the p277 cassette containing the human *dhfr* sequence (Guttery et al., 2012) (Figure S2). For gene deletion by double homologous recombination, a targeting vector was generated using the pBS-DHFR cassette (Tewari et al., 2010) (Figure S3). Both vectors confer resistance to pyrimethamine. Linear targeted sequences were released by restriction digest and transfected via electroporation (Janse et al., 2006) into either the *P. berghei ANKA* 2.34 strain or the *P. berghei ANKA* 507 cl1 line (Janse et al., 2006). For initial genotyping of parasite pools PCR and Western blotting were used for GFP-tagged lines and PCR alone for deletion mutants. After dilution cloning of mutants, deletion of the targeted region of the genome was confirmed by PCR, Southern

blotting and pulse-field electrophoresis (PFGE). See Table S5 for all oligonucleotides used for cloning in this study.

Phenotypic screening of mutants and GFP localisation

Phenotypic screening of mutants and localisation of GFP-tagged lines was performed as previously described (Guttery et al., 2012; Tewari et al., 2010). Briefly, asexual proliferation and gametocytogenesis and gametocyte sex ratios were analysed using Giemsa-stained blood smears. Exflagellation was examined on day 4–5 post infection. 10 µl of gametocyte-infected blood were obtained from the tail with a heparinised pipette tip and mixed immediately with 40 µl of ookinete culture medium (RPMI 1640 containing 25 mM HEPES, 20% foetal bovine serum, 10 mM sodium bicarbonate, and 50 mM xanthurenic acid at pH 7.6). The mixture was placed under a Vaseline-coated cover slip and 15 min later exflagellation centres were counted by phase contrast microscopy in 12–15 fields. Ookinete formation was assessed the next day; 10 µl of infected tail blood were obtained as above, mixed immediately with 40 µl ookinete culture medium, and incubated for 2 hr at 20 °C to allow completion of gametogenesis and fertilisation. Each culture was then diluted with 0.45 ml of ookinete medium and incubated at 20 °C for a further 21–24 hr to allow ookinete differentiation. Cultures were pelleted for 2 min at 5000 rpm and then incubated with 10 µl of ookinete medium containing Hoechst 33342 DNA dye and Cy3-conjugated mouse monoclonal antibody 13.1 (Tewari et al., 2005) recognising the P28 protein on the surface of ookinetes and any undifferentiated macrogametes or zygotes. P28-positive cells were counted with a Zeiss AxioImager M2 microscope (Carl Zeiss, Inc) fitted with an AxioCam ICc1 digital camera. Ookinete conversion was expressed as the percentage of P28 positive parasites that had differentiated into ookinetes. To measure nuclear DNA content of ookinetes by direct immunofluorescence, images of parasites stained as above were analysed using ImageJ software (National Institutes of Health) as previously described (Tewari et al., 2005). Motility assays were performed as described previously (Patzewitz et

al., 2013). Briefly, ookinete cultures were mixed with an equal volume of Matrigel on ice, mounted onto a slide, covered and sealed with nail varnish and left to set at room temperature for at least 30 min. Time lapse movies of ookinete movement (1 frame every 5 s for 10 min) were taken using a Zeiss AxioImager M2 microscope as described above, and their speed was determined (µm min^{-1}).

For mosquito transmission experiments, triplicate sets of 20–50 *Anopheles stephensi* SD500 mosquitoes were allowed to feed for 20 min on anaesthetised infected mice whose asexual parasitaemia had reached 5–7% and were carrying comparable numbers of gametocytes as determined on Giemsa stained blood films. On day 14 post-feeding approximately 20 mosquitoes were dissected and oocysts on their mid-guts counted. Oocyst formation was examined by Hoechst 33342 staining for 10–15 min and guts were washed and mounted under Vaseline-rimmed cover slips. Images were recorded using a Zeiss AxioImager M2 microscope fitted with an AxioCam ICc1 digital camera. Oocyst diameter was measured using AxioVision software. On day 21 post-feeding another 20 mosquitoes were dissected and their guts and salivary glands homogenised separately in a loosely fitting homogeniser to release sporozoites, which were then quantified using a haemocytometer. Due to day-to-day variations in transmission levels, all data were normalised to a matching number of wild type controls analysed on the same day.

Genetic complementation crosses were carried out between different mutant parasite lines as previously described (Guttery et al., 2012; Patzewitz et al., 2013). Briefly, for complementation of ookinete conversion, mature gametocyte-containing blood from mice infected with different parasite lines was mixed and re-suspended in ookinete medium, and ookinete conversion was determined as described above. For complementation of oocyst production, mice were infected with combinations of different parasite strains and 3-6 days old female *A. stephensi* mosquitoes were infected by directly feeding on these mice.

Mosquitoes were dissected 12-14 days post infection and the presence of oocysts was determined as described above.

Purification of schizonts, gametocytes and ookinetes

Blood stage parasites taken from infected mice (day 4 post infection) were placed in culture (40 ml RPMI 1640, 8 ml foetal bovine serum, 0.5 ml penicillin and streptomycin; per 0.5 ml blood) for 24 h at 37 °C (with rotation at 100 rpm). The following day the culture was fractionated on a 60% v/v NycoDenz gradient (NycoDenz stock solution: 27.6% w/v NycoDenz in 5 mM Tris-HCl, pH 7.20, 3 mM KCl, 0.3 mM EDTA; CLB: PBS, 20 mM HEPES, 20 mM Glucose, 4 mM sodium bicarbonate, 1 mM EGTA, 0.1% w/v bovine serum albumin, pH 7.25). The purification of gametocytes was based on a modified protocol from (Beetsma et al., 1998). On day four post-infection mice were treated with sulfadiazine (Sigma, 20 mg/l in drinking water) for two days to eliminate asexual blood stage parasites. On day six post-infection the parasites were harvested, kept on ice to avoid premature activation and separated from uninfected erythrocytes on a 48% (v/v) NycoDenz in coelenterazine loading buffer (CLB) gradient. Gametocytes were harvested from the interface and washed twice in RPMI 1640 before activation of gamete formation in ookinete medium for 30 min at 20 ^{o}C. For ookinete preparation, parasites from day 5 post infection mice were placed in 1 ml ookinete medium for 24 hr at 20 °C for ookinete production. The parasites were then lysed in red blood cell (RBC) buffer for 30 min and purified on a 63% NycoDenz gradient (v/v in CLB).

Isolation of PPM2-GFP and PPM5-GFP proteins, and subcellular fractionation

Immunoprecipitation and subcellular fractionation of GFP-tagged proteins were performed as described previously (Guttery et al., 2012). Briefly, the cell pellets obtained from blood of mice infected with parasites expressing GFP, PPM2-GFP and PPM5-GFP were resuspended in hypotonic lysis buffer (10 mM Tris-HCl pH 8.4, 5 mM EDTA) containing

protease inhibitors (Roche), freeze/thawed twice, incubated for 1 hr at 4°C and centrifuged at 100,000 g for 30 min. The supernatants obtained were collected as the soluble protein fraction (S). The corresponding pellets were then washed, resuspended in carbonate solution (0.1M Na_2CO_3, pH 11.0) containing protease inhibitors (Roche), incubated for 30 min at 4°C and centrifuged again at 100,000 g for 30 min. The resulting supernatants were saved as the peripheral membrane fraction (PM) and the pellets were washed and solubilised in 4% SDS and 0.5% Triton X-100 in PBS, forming the integral membrane fraction (IM). Equal amounts of these three fractions were then analysed by western blot using anti-GFP antibody.

PPM2 and PPM5 *in vivo* phosphorylation

As described previously (Guttery et al., 2012), schizonts and activated gametocytes (purified as described above) were washed in phosphate-free Krebs buffer and metabolically labelled with 3–5 MBq [^{32}P]-orthophosphate (Perkin Elmer) in the same buffer for 30 min at 20 °C or 37 °C for activated gametocytes and schizonts, respectively. Following two washes in phosphate-free Krebs buffer, the labelled parasites were lysed for 30 min at 4 °C in lysis buffer (10 mM Tris-HCl pH 7.5, 150 mM NaCl, 0.5 mM EDTA, 0.5% NP-40) supplemented with protease and phosphatase inhibitors (both Roche). The resulting lysate was centrifuged at 20,000 g for 10 min and the supernatant collected. GFP tagged proteins were then isolated using GFP-TRAP beads (ChromoTek) according to the manufacturer's instructions and the immunoprecipitated proteins were subsequently resuspended in Laemmli sample buffer for separation by SDS-PAGE. [^{32}P]-labelled proteins were visualised using a phosphorimager (Molecular Dynamics) and GFP-tagged proteins analysed by Western blot using anti-GFP antibody.

Phosphatase activity assay

Protein phosphatase activity of the immunoprecipitated PPM2-GFP and PPM5-GFP was assessed using the SensoLyte MFP Protein Phosphatase Assay Kit (AnaSpec) according to manufacturer's instructions. Briefly, blood aliquots from infected mice (with GFP, PPM2-GFP and PPM5-GFP parasites) were processed as described previously (Guttery et al., 2012). The parasite pellets were lysed for 30 min at 4 °C in lysis buffer (ChromoTek) supplemented with protease inhibitors (Roche), and the resulting lysates were then immunoprecipitated using GFP-TRAP beads (ChromoTek) according to manufacturer's instructions. The GFP-TRAP beads were resuspended and diluted in phosphatase assay buffer (100 mM Tris-HCl pH 7.5, 4 mM DTT, 0.2 mM EDTA, 0.5 mM $MnCl_2$, 0.4 mg/ml BSA), incubated for 30 min at 37 °C in the presence or absence of MFP fluorogenic phosphatase substrate, and centrifuged for 2 min at 2700 g. Supernatants were transferred to a 96-well microplate and the fluorescence generated by the dephosphorylation of MFP was measured using a microplate fluorimeter.

Metabolic labelling, purification and detection of *N*-myristoylated proteins

To metabolically label *N*-myristoylated proteins, a recently described procedure was followed (Poulin et al., 2013; Wright et al., 2014). Briefly the blood of one infected mouse was placed in schizont medium containing 50 µM YnMyr and left overnight at 37 °C before purification as described above. Parasite proteins were extracted using 0.1% SDS, 1% Triton X-100 in 10 mM Na_2PO_4, pH 8.2 with protease inhibitors (EDTA-free, Roche). Extracts were pelleted and the concentration of protein in the supernatant determined by DC protein assay (Bio-Rad). Protein lysates were labelled and precipitated as described previously (Heal et al., 2012). Protein was redissolved at 10 mg/ml in 2% SDS, 10 mM EDTA in PBS, and then diluted to 1 mg/ml with PBS. Aliquots were removed for pre-enrichment analysis. Proteins were incubated with Dynabeads MyOne Streptavidin C1 for 2 hr at RT. Following removal of the supernatant, beads were washed with 1% SDS in PBS, and then boiled for 10 min in sample loading buffer to elute bound proteins. For immunoblotting, proteins were transferred

to PVDF membranes, membranes were blocked (5% dried skimmed milk in TBS 0.1% Tween-20), then probed with anti-GFP (rabbit polyclonal, 1:2,000, Invitrogen), followed by anti-rabbit HRP secondary antibody (goat anti-rabbit, 1:10,000, Invitrogen) in blocking solution, and developed with Luminata Crescendo Western HRP substrate (Millipore) according to the manufacturer's instructions on a Fujifilm LAS 3000 imager.

Electron microscopy

Gametocyte and ookinete samples cultured in ookinete medium as described above were fixed in 4% glutaraldehyde in 0.1 M phosphate buffer and processed for routine electron microscopy as previously described (Guttery et al., 2012). Samples were post fixed in osmium tetroxide, treated en bloc with uranyl acetate, dehydrated and embedded in Spurr's epoxy resin. Thin sections were stained with uranyl acetate and lead citrate prior to examination in a JEOL1200EX electron microscope (Jeol UK Ltd).

Quantitative RT-PCR

Total RNA was isolated from purified parasites using an RNeasy purification kit (Qiagen). For qRT-PCR, cDNA was synthesised using an RNA-to-cDNA kit (Applied Biosystems) allowing quantification from 250 ng of total RNA. qRT-PCR reactions consisted of 2 µl cDNA, 5 µl SYBR green fast master mix (Applied Biosystems), 0.5 µl (500 nM) each of the forward and reverse primers, and 2 µl DEPC-treated water. Where possible, one of the primer pairs was placed over an intron, primers had melting temperatures of 60-62 ^{o}C and together amplified a region 70-200 bp long. Analysis was conducted using an Applied Biosystems 7500 fast machine with the following cycling conditions: 95 °C for 20 sec followed by 40 cycles of 95 °C for 3 sec; 60 °C for 30 sec. Wild-type expression was determined using the Pfaffl method (Pfaffl, 2001). Relative quantification in the mutant line was normalised against wild-type expression using the $\Delta\Delta C_t$ method. Both methods used *hsp70* (PBANKA_081890) and *seryl-tRNA synthetase* (PBANKA_061540) as reference genes. Three biological

replicates were used for each stage (each with two technical replicates). See Table S6 for a full list of the primers used for qRT-PCR.

Transcriptome sequencing and RNA-Seq analysis

Parasites were collected from Δ*ppm2*, Δ*ppm5* or GFP-expressing lines at three developmental stages (schizonts, activated gametocytes and ookinetes). Total RNA was isolated from purified parasites using an RNeasy purification kit (Qiagen) as described for qRT-PCR but was also passed through a plasmodipur column to remove host DNA contamination prior to RNA isolation. RNA was vacuum concentrated (SpeedVac) and transported using RNA stable tubes (Biomatrica). Validation used different biological replicates to the RNA-seq samples to validate the data set more robustly (Allison et al., 2006). Strand-specific mRNA sequencing was performed from total RNA using TruSeq Stranded mRNA Sample Prep Kit LT (Illumina) according to manufacturer's instructions. Briefly, polyA+ mRNA was purified from total RNA using oligo-dT dynabead selection. First strand cDNA was synthesised using randomly primed oligos followed by second strand synthesis where dUTPs were incorporated to achieve strand-specificity. The cDNA was adapter-ligated and the libraries amplified by PCR. Libraries were sequenced in Illumina Hiseq with paired-end 100bp read chemistry.

RNA-seq read alignment and differential gene expression analysis were performed using the Tophat-Cufflinks pipeline (Trapnell et al., 2012). Strand-specific RNA-seq paired-end reads were mapped onto the *P. berghei* ANKA genome (PlasmoDB-9.2) using TopHat version 2.0.8 (Trapnell et al., 2009) with options '--library-type=fr-firststranded' and '--no-novel-juncs'. The aligned reads were quantified, normalised andcompared across different samples (2-4 biological replicates per sample) using Cuffdiff version 2.1 (Trapnell et al., 2013). *P. berghei* genome sequence was provided as a FASTA file to Cuffdiff to account for sequence bias in read alignment and a gff file consisting of highly variable tRNA, rRNA and

mitochondrial genes was provided to mask them from further analysis. The Cuffdiff output was visualised using R package CummeRbund (http://compbio.mit.edu/cummeRbund/index.html). The correlation between biological replicates vary from r^2 = 0.8-0.98, therefore were tightly correlated.

P. berghei phosphatome interaction network

The microarray data of P. *falciparum* global transcriptional responses to 20 growth-inhibiting compounds (Hu et al., 2010) was used to build the phosphatases interaction subnetwork (Figure S6B). DNA microarray-based profiling of growth perturbations in *P. falciparum* was previously used to generate a high-resolution transcriptional data set that reflects functional relationships between P. *falciparum* genes (Hu et al., 2010). 21 P. *falciparum* phosphatases corresponding to 21 P. *berghei* orthologoues were included in the growth perturbations dataset. We used a general method (Feizi et al., 2013), called "Network Deconvolution (ND)", which infers direct effects from an observed correlation matrix containing both direct and indirect effects. First, we applied the context likelihood of relatedness (CLR) algorithm (Faith et al., 2007) to the growth perturbations dataset to construct a relevance network. ND was then applied to the CLR-predicted network. The top 10% (15,826 linkages) and 50% (76,366 linkages) of edge predictions were used to construct two independent networks.

From each network, a phosphatase subnetwork was extracted, which we hereafter refer to as top 10% and top 50% phosphatase subnetworks. Both networks were then transformed by orthology into *P. berghei* using the "Transform by Orthology" tool in PlasmoDB http://plasmodb.org/plasmo/. The connectivity of the top 10% phosphatases subnetwork fit a power-law distribution with power (λ) value of 1.009 (R^2 = 0.78). This distribution represents a typical scale-free network, well known for protein-protein interaction networks in eukaryotic cells: a small number of highly connected nodes (hubs) are linked to a large number of less connected nodes. For the top 50% subnetwork, λ was 0.649 (R^2 = 0.523). To distinguish direct targets for PPM2 and PPM5, we tested the lists of significantly affected genes in

Δ*ppm2 and* Δ*ppm5* for overlap with the interaction partners of PPM2 and PPM5, respectively using the "GeneOverlap" package (http://shenlab-sinai.github.io/shenlab-sinai/) and Pearson's Chi-squared test. Results for the numbers of interaction partners for each of the phosphatases (PPM2 and PPM5), numbers of differentially expressed genes (q-value ≤ 0.01) and *p-value* for overlap are listed in Figure S6D. Biolayout (http://www.biolayout.org/, (Enright and Ouzounis, 2001) was used for visualisation. MCL (Markov-Cluster Algorithm http://micans.org/mcl/) was used for interaction subnetworks where PPM2 and PPM5 co-clustered.

Data deposition

RNA-Seq data has been deposited at the European Nucleotide Archive (ENA - http://www.ebi.ac.uk/ena) under accession number PRJEB5218.

Computational modelling

The sequences of all PPs were analysed by computational structural methods. All sequences were submitted to the RaptorX (raptorx.uchicago.edu/), Phyre^2 (www.sbg.bio.ic.ac.uk/phyre2/) and Swiss-Model (swissmodel.expasy.org/) servers. For PPMs with extensive loop regions, sequences with pruned loops were also submitted. Models obtained were further refined using the ModRefiner server (zhanglab.ccmb.med.umich.edu/ModRefiner/). Models were visualised and analysed using Pymol (www.pymol.org).

Gene ontology (GO) terms enrichment analysis

GO IDs were extracted from the *P. berghei* ANKA annotation gff file (available in GeneDB) and the differentially expressed gene lists were tested against a filtered universal list of 2133 *P. berghei* genes with annotated GO terms. Enriched GO terms (p value <0.05) were

identified through conditional hypergeometric testing using GOstats R package (Falcon and Gentleman, 2007) and plotted using ggplot2 R package (http://ggplot2.org/).

SUPPLEMENTAL REFERENCES

Allison, D.B., Cui, X., Page, G.P., and Sabripour, M. (2006). Microarray data analysis: from disarray to consolidation and consensus. Nature reviews Genetics *7*, 55-65.

Anisimova, M., Cannarozzi, G.M., and Liberles, D.A. (2010). Finding the balance between the mathematical and biological optima in multiple sequence alignment. Trends Evol Biol *2*, e7.

Beetsma, A.L., van de Wiel, T.J., Sauerwein, R.W., and Eling, W.M. (1998). Plasmodium berghei ANKA: purification of large numbers of infectious gametocytes. Experimental parasitology *88*, 69-72.

Bologna, G., Yvon, C., Duvaud, S., and Veuthey, A.L. (2004). N-Terminal myristoylation predictions by ensembles of neural networks. Proteomics *4*, 1626-1632.

Eddy, S.R. (2009). A new generation of homology search tools based on probabilistic inference. Genome informatics International Conference on Genome Informatics *23*, 205-211.

Enright, A.J., and Ouzounis, C.A. (2001). BioLayout--an automatic graph layout algorithm for similarity visualization. Bioinformatics *17*, 853-854.

Faith, J.J., Hayete, B., Thaden, J.T., Mogno, I., Wierzbowski, J., Cottarel, G., Kasif, S., Collins, J.J., and Gardner, T.S. (2007). Large-scale mapping and validation of Escherichia coli transcriptional regulation from a compendium of expression profiles. PLoS Biol *5*, e8.

Falcon, S., and Gentleman, R. (2007). Using GOstats to test gene lists for GO term association. Bioinformatics *23*, 257-258.

Feizi, S., Marbach, D., Medard, M., and Kellis, M. (2013). Network deconvolution as a general method to distinguish direct dependencies in networks. Nat Biotechnol *31*, 726-733.

Guindon, S., and Gascuel, O. (2003). A simple, fast, and accurate algorithm to estimate large phylogenies by maximum likelihood. Systematic biology *52*, 696-704.

Guttery, D.S., Poulin, B., Ferguson, D.J., Szoor, B., Wickstead, B., Carroll, P.L., Ramakrishnan, C., Brady, D., Patzewitz, E.M., Straschil, U.*, et al.* (2012). A unique protein phosphatase with kelch-like domains (PPKL) in Plasmodium modulates ookinete differentiation, motility and invasion. PLoS pathogens *8*, e1002948.

Heal, W.P., Wright, M.H., Thinon, E., and Tate, E.W. (2012). Multifunctional protein labeling via enzymatic N-terminal tagging and elaboration by click chemistry. Nature protocols *7*, 105-117.

Hu, G., Cabrera, A., Kono, M., Mok, S., Chaal, B.K., Haase, S., Engelberg, K., Cheemadan, S., Spielmann, T., Preiser, P.R.*, et al.* (2010). Transcriptional profiling of growth perturbations of the human malaria parasite Plasmodium falciparum. Nature biotechnology *28*, 91-98.

Janse, C.J., Franke-Fayard, B., Mair, G.R., Ramesar, J., Thiel, C., Engelmann, S., Matuschewski, K., van Gemert, G.J., Sauerwein, R.W., and Waters, A.P. (2006). High efficiency transfection of Plasmodium berghei facilitates novel selection procedures. Molecular and biochemical parasitology *145*, 60-70.

Katoh, K., Misawa, K., Kuma, K., and Miyata, T. (2002). MAFFT: a novel method for rapid multiple sequence alignment based on fast Fourier transform. Nucleic acids research *30*, 3059-3066.

Patzewitz, E.M., Guttery, D.S., Poulin, B., Ramakrishnan, C., Ferguson, D.J., Wall, R.J., Brady, D., Holder, A.A., Szoor, B., and Tewari, R. (2013). An ancient protein phosphatase, SHLP1, is critical to microneme development in Plasmodium ookinetes and parasite transmission. Cell reports *3*, 622-629.

Pfaffl, M.W. (2001). A new mathematical model for relative quantification in real-time RT-PCR. Nucleic acids research *29*, e45.

Poulin, B., Patzewitz, E.M., Brady, D., Silvie, O., Wright, M.H., Ferguson, D.J., Wall, R.J., Whipple, S., Guttery, D.S., Tate, E.W., *et al.* (2013). Unique apicomplexan IMC sub-compartment proteins are early markers for apical polarity in the malaria parasite. Biology open *2*, 1160-1170.

Tewari, R., Dorin, D., Moon, R., Doerig, C., and Billker, O. (2005). An atypical mitogen-activated protein kinase controls cytokinesis and flagellar motility during male gamete formation in a malaria parasite. Molecular microbiology *58*, 1253-1263.

Tewari, R., Straschil, U., Bateman, A., Bohme, U., Cherevach, I., Gong, P., Pain, A., and Billker, O. (2010). The systematic functional analysis of Plasmodium protein kinases identifies essential regulators of mosquito transmission. Cell host & microbe *8*, 377-387.

Trapnell, C., Hendrickson, D.G., Sauvageau, M., Goff, L., Rinn, J.L., and Pachter, L. (2013). Differential analysis of gene regulation at transcript resolution with RNA-seq. Nature biotechnology *31*, 46-53.

Trapnell, C., Pachter, L., and Salzberg, S.L. (2009). TopHat: discovering splice junctions with RNA-Seq. Bioinformatics *25*, 1105-1111.

Trapnell, C., Roberts, A., Goff, L., Pertea, G., Kim, D., Kelley, D.R., Pimentel, H., Salzberg, S.L., Rinn, J.L., and Pachter, L. (2012). Differential gene and transcript expression analysis of RNA-seq experiments with TopHat and Cufflinks. Nature protocols *7*, 562-578.

Wickstead, B., and Gull, K. (2007). Dyneins across eukaryotes: a comparative genomic analysis. Traffic *8*, 1708-1721.

Wickstead, B., Gull, K., and Richards, T.A. (2010). Patterns of kinesin evolution reveal a complex ancestral eukaryote with a multifunctional cytoskeleton. BMC evolutionary biology *10*, 110.

Wright, M.H., Clough, B., Rackham, M.D., Rangachari, K., Brannigan, J.A., Grainger, M., Moss, D.K., Bottrill, A.R., Heal, W.P., Broncel, M., *et al.* (2014). Validation of N-myristoyltransferase as an antimalarial drug target using an integrated chemical biology approach. Nat Chem *6*, 112-121.
